# Safety, acceptability, and pharmacokinetics of a monoclonal antibody-based vaginal multipurpose prevention film (MB66): A Phase I randomized trial

**DOI:** 10.1371/journal.pmed.1003495

**Published:** 2021-02-03

**Authors:** Joseph A. Politch, Susan Cu-Uvin, Thomas R. Moench, Karen T. Tashima, Jai G. Marathe, Kate M. Guthrie, Howard Cabral, Tara Nyhuis, Miles Brennan, Larry Zeitlin, Hans M. L. Spiegel, Kenneth H. Mayer, Kevin J. Whaley, Deborah J. Anderson

**Affiliations:** 1 Boston University School of Medicine, Department of Medicine, Boston, Massachusetts, United States of America; 2 Alpert Medical School of Brown University, Department of Obstetrics and Gynecology and Medicine, Providence, Rhode Island, United States of America; 3 Mapp Biopharmaceutical Inc., San Diego, California, United States of America; 4 Division of Infectious Diseases, Alpert Medical School of Brown University, Providence, Rhode Island, United States of America; 5 Department of Psychiatry and Human Behavior, Alpert Medical School of Brown University, Providence, Rhode Island, United States of America; 6 Department of Biostatistics, Boston University School of Public Health, Boston, Massachusetts, United States of America; 7 Kelly Government Solutions, Contractor to National Institute of Allergy and Infectious Diseases, National Institutes of Health, Department of Health and Human Services, Rockville, Maryland, United States of America; 8 Harvard Medical School, Department of Medicine, Boston, Massachusetts, United States of America; HIV Vaccine Trials Network, UNITED STATES

## Abstract

**Background:**

MB66 film is a multipurpose prevention technology (MPT) product with monoclonal antibodies (mAbs) against HIV-1 (VRC01-N) and HSV-1 and 2 (HSV8-N). The mAbs were produced by transient expression in *Nicotiana benthamiana* (N). We conducted a Phase I clinical trial to assess the safety, pharmacokinetics (PK), and ex vivo efficacy of single and repeated doses of MB66 when used intravaginally.

**Methods and findings:**

The clinical trial enrolled healthy reproductive-aged, sexually abstinent women. In Segment A, 9 women received a single MB66 film which was inserted into the vaginal posterior fornix by a clinician. In Segment B, 29 women were randomly assigned to MB66 (Active) or Placebo film groups and were instructed to insert 1 film vaginally for 7 consecutive days. Visits and clinical sampling occurred predose and at various time points after single and repeated film doses. The primary endpoint was number of adverse events (AEs) Grade 2 or higher related to product use. Secondary endpoints included film dissolution rate, Nugent score (a Gram stain scoring system to diagnose bacterial vaginosis), vaginal pH, post-use survey results, cytokine concentrations in cervicovaginal lavage (CVL) specimens (assessed by Luminex assay), mAb concentrations in vaginal fluid collected from 4 sites (assessed by ELISA), and HIV and HSV neutralization activity of CVL samples ex vivo (assessed by TZM-bl and plaque reduction assay, respectively).

The product was generally safe and well tolerated, with no serious AEs recorded in either segment. The AEs in this study were primarily genitourinary in nature with the most commonly reported AE being asymptomatic microscopic hematuria. There were no differences in vaginal pH or Nugent scores or significant increases in levels of proinflammatory cytokines for up to 7 days after film insertion in either segment or between Active and Placebo groups. Acceptability and willingness to use the product were judged to be high by post-use surveys.

Concentrations of VRC01-N and HSV8-N in vaginal secretions were assessed over time to generate pharmacokinetic curves. Antibody levels peaked 1 hour postdosing with Active film (median: 35 μg/mL) and remained significantly elevated at 24 hours post first and seventh film (median: 1.8 μg/mL). Correcting for sample dilution (1:20), VRC01-N concentrations ranged from 36 to 700 μg/mL at the 24-hour time point, greater than 100-fold the IC_50_ for VRC01 (0.32 μg/mL); HSV8-N concentrations ranged from 80 to 601 μg/mL, well above the IC_50_ of 0.1 μg/m. CVL samples collected 24 hours after MB66 insertion significantly neutralized both HIV-1 and HSV-2 ex vivo. Study limitations include the small size of the study cohort, and the fact that no samples were collected between 24 hours and 7 days for pharmacokinetic evaluation.

**Conclusions:**

Single and repeated intravaginal applications of MB66 film were safe, well tolerated, and acceptable. Concentrations and ex vivo bioactivity of both mAbs in vaginal secretions were significantly elevated and thus could provide protection for at least 24 hours postdose. However, further research is needed to evaluate the efficacy of MB66 film in women at risk for HIV and HSV infection. Additional antibodies could be added to this platform to provide protection against other sexually transmitted infections (STIs) and contraception.

**Trial registration:**

ClinicalTrials.gov NCT02579083.

## Abstract 2

Joseph Politch and co-workers report on a first-in-human evaluation of a vaginal film deploying monoclonal antibodies to target viral infections.

## Author summary

**Why was this study done?:**

- Human immunodeficiency virus-type 1 (HIV-1) and herpes simplex virus-type 2 (HSV-2) are 2 relatively common sexually transmitted pathogens associated with significant morbidity and mortality.
- Antiviral drugs have been introduced to suppress viral concentrations and ameliorate some of the worst effects of these viruses, but the infections are incurable. Therefore, considerable effort is being directed toward prevention strategies.
- Many women have a preference for multipurpose prevention technology (MPT) products, effective short-term pericoital methods and natural products (i.e., antibodies).

**What did the researchers do and find?:**

- We conducted a Phase I clinical trial to assess the safety, acceptability, pharmacokinetics (PK), and ex vivo efficacy of single and repeated doses of MB66, a vaginal film product containing monoclonal antibodies (mAbs) against HIV-1 (VRC01-N) and HSV-1 and 2 (HSV8-N).
- No serious adverse events (AEs) associated with product use were observed.
- Antibody levels peaked in vaginal secretions 1 hour postdosing with Active film and remained significantly elevated through 24 hours.
- Vaginal samples collected 24 hours after MB66 insertion significantly neutralized both HIV-1 and HSV-2 ex vivo.

**What do these findings mean?:**

- Single and repeated intravaginal applications of MB66 film were safe, well tolerated, and acceptable.
- Concentrations and ex vivo bioactivity of both mAbs in vaginal secretions were consistent with protection for at least 24 hours after film use.
- Phase II/III clinical trials are needed to further evaluate safety and efficacy of MB66 film in women at risk for HIV and HSV infection.

## Introduction

Human immunodeficiency virus-type 1 (HIV-1) and herpes simplex virus-type 2 (HSV-2) are 2 relatively common and serious sexually transmitted pathogens. Over 35 million people are currently infected with HIV-1 which can cause severe immunodeficiency leading to death [1]. An estimated 1-in-4 adults has been infected with HSV-2, a pathogen that can cause genital ulcerations, severe pathology in newborns, and is associated with an increased risk of HIV-1 acquisition and transmission in men and women [2,3]. Antiviral drugs have been introduced to suppress viral concentrations and ameliorate some of the worst effects of these viruses, but the infections are incurable. Therefore, considerable effort is being directed toward prevention strategies. To date, there are no effective vaccines to prevent HIV or HSV transmission. Condoms are safe and effective when used consistently and correctly but are generally perceived as a barrier to intimacy and sexual pleasure and have relatively low acceptability among both men and women [4]. Antiviral drugs, when used consistently, also can significantly reduce HIV and HSV levels in genital secretions and viral transmission or acquisition. However, many HIV/HSV uninfected individuals are not motivated to use these drugs daily to prevent viral infection.

A number of topical microbicides, agents designed to reduce the incidence of sexually transmitted infections, have been tested with largely disappointing results. The over-the-counter vaginal spermicide nonoxynol-9 (N-9) displayed strong antiviral activity in laboratory tests due to its detergent properties but caused damage to the vaginal epithelium and failed to show efficacy in preventing HIV-1 infection in clinical trials [5]. Other microbicide candidates with broad, nonspecific mechanisms of action including C31G (Savvy; a detergent), BufferGel (an acidifier), PRO2000 (a polyanion), cellulose sulfate (a polyanion), and Carraguard (a polyanion) also failed to protect against HIV-1 infection in clinical trials [5].

More specific anti-HIV-1 drugs have been the focus of recent vaginal microbicide development. Studies evaluating the efficacy of the antiretroviral drug tenofovir in a hydroxyethyl cellulose (HEC) gel have had the most success. Tenofovir gel (1%) reduced HIV infections by 39% in high-risk African women (CAPRISA 004 trial), when the gel was administered within 12 hours precoitus and again within 12 hours postcoitus [5,6], and the efficacy increased if participants were at least 80% adherent. However, 1% Tenofovir gel applied once daily independent of intercourse (VOICE trial) did not protect against HIV-1 transmission [5,7]. Likewise, the FACTS 001 study using the CAPRISA 004 dosing regimen failed to demonstrate efficacy, primarily due to low levels of objectively measured adherence [5,8]. Phase III trials of an intravaginal ring (IVR) containing dapivirine (the ASPIRE, RING, HOPE, and DREAM studies) demonstrated significant protection overall, with increased protection in groups with higher adherence [9,10]. Recent surveys have shown that many women have a preference for an effective short-term pericoital method and would rather not use antiretroviral drugs to prevent HIV infection [11]. In addition, they have shown a strong preference for multipurpose prevention technology (MPT) products that can provide protection against several sexually transmitted infection (STI) pathogens with or without contraception [12–14].

Antibodies have been administered to people for the prevention and treatment of diseases for over a century [15]. Recently, human monoclonal antibodies (mAbs) have become an accepted form of treatment for a number of diseases; over 90 mAbs have been licensed for clinical use in the United States and European Union [16,17]. Potent broadly neutralizing HIV-specific mAbs are currently being administered intravenously or subcutaneously in clinical trials for HIV prevention and therapy [18]. MAbs also show promise as topical microbicides because of their specificity, flexibility, and overall favorable safety profile. Human vaginal secretions contain various natural antibodies [19]; therefore, addition of human mAbs can potentially enhance natural defense mechanisms. Studies in nonhuman primates demonstrated that anti-HIV mAbs protect against SHIV vaginal transmission when delivered either systemically or vaginally immediately before vaginal challenge [15]. Two pilot Phase I clinical trials conducted in Europe of vaginally applied HIV antibodies [20,21] provided initial data that this approach is safe in women. We have developed a microbicide film, MB66, which contains a combination of 2 human mAbs: VRC01 [22], which is directed against HIV-1, and HSV8 [23], which is directed against HSV-1 and 2. VRC01 is a potent neutralizer of HIV, with an EC_50_ of <1 μg/mL for the majority of a panel of diverse HIV isolates [24]. HSV8 binds to glycoprotein D of both HSV-1 and 2 and is a potent neutralizer of both viruses, with an EC_50_ of <1 μg/mL [25,26]. HSV8 has been shown to provide in vivo protection in the HSV-2 mouse vaginal challenge model, with EC_50_ and EC_90_ of 0.1 μg/mL and 1 μg/mL, respectively [23]. There have been no previous studies of HSV8 in humans. The mAbs were produced using a rapid, cost-effective *Nicotiana benthamiana* platform [27]. We report here the first-in-human Phase I clinical trial of an antibody-based MPT vaginal film with a number of safety and ex vivo efficacy endpoints.

## Materials and methods

### Ethics statement

The study was approved by the NIAID Division of AIDS (DAIDS) and the Institutional Review Boards of both The Miriam Hospital (Human Subjects Protocol #763714) and Boston University Medical Campus (Human Subjects Protocol #H-34209) and was registered at ClinicalTrials.gov (NCT No. NCT02579083). See S1 CONSORT Checklist for randomized clinical trial details.

### Study design and participants

MB66-01 is a Phase I study to assess the safety of MB66, a vaginal microbicide film containing a combination of anti-HIV (VRC01-N) and anti-HSV (HSV8-N) mAbs. The study was coordinated by Boston University School of Medicine (BUSM) under an IND sponsored by Mapp Biopharmaceutical (San Diego, California, USA), and conducted at The Miriam Hospital, Providence, Rhode Island, from January 18, 2016, to July 23, 2018, with data compiled and analyzed at BUSM.

MB66-01 had 2 sequential segments: Segment A, a single-arm, single-dose study followed by a pause for safety assessment, and Segment B, a two-arm, repeated-dose, randomized, single-blind, placebo-controlled trial. Segment B utilized a parallel group design with a 1:1 allocation ratio. Random assignment of enrolled Segment B participants to Active group (MB66 film) or Placebo film group (film without antibodies) was performed in blocks of 6 via a list of ID numbers previously created by the study statistician utilizing SAS statistical software (Version 9.4; SAS Institute, Cary, North Carolina, USA). Random assignment was performed by the study pharmacist via 2 coded envelopes containing Active and Placebo films. The group identity remained coded throughout the trial and was not revealed to study staff until after all the data were compiled and the statistical analyses were complete.

Recruitment, written informed consent, and enrollment were conducted by study clinicians at The Miriam Hospital per the protocol approved by DAIDS (See S1 Study Protocol). Target enrollment was 8 participants for Segment A and 30 participants (15 per study arm) for Segment B. Women were eligible if they were 18 to 45 years of age, healthy, HIV-uninfected, at low risk of HIV/STI acquisition, had normal menstrual cycles, were not pregnant and were using a reliable method of contraception, and were premenopausal women with an intact uterus. It should be noted that prescreening for HSV-1 and HSV-2 serostatus was not performed since it was expected that only a minority of potential participants would be seronegative for both viruses. Preexisting antibodies against HSV-1 and HSV-2 constitute a background in HSV8 antibody tests. However, it was judged to be impractical to recruit for the study if double-HSV-seronegativity was required in addition to the other inclusion and exclusion criteria. Complete lists of inclusion and exclusion criteria are presented in S1 and S2 Tables, respectively.

**Fig 1:**
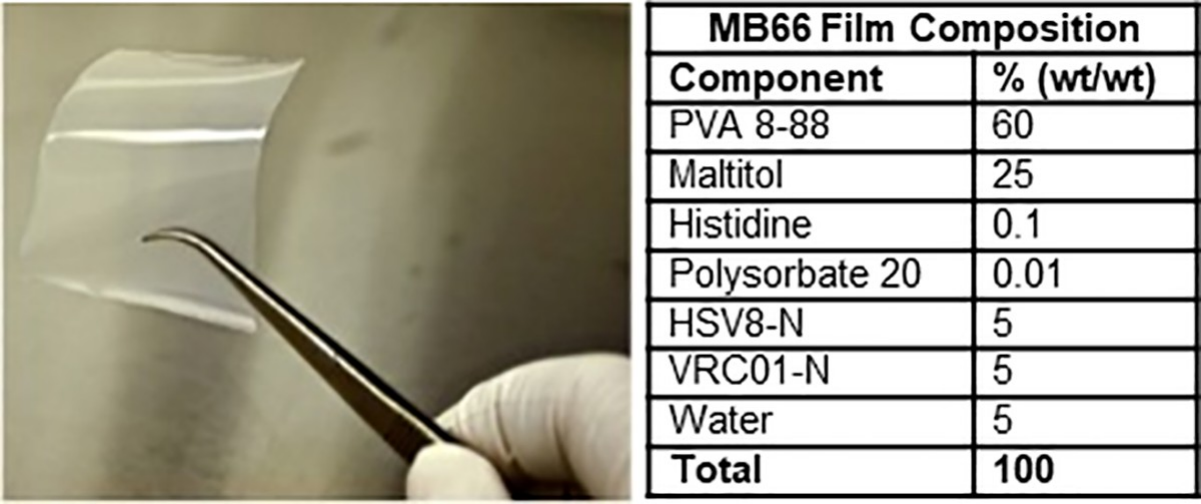
MB66 film example and composition.

The MB66 product contains 10 mg each of VRC01-N (anti-HIV-1 mAb) and HSV8-N (anti-HSV-2 mAb) in a 2-inch × 2-inch polyvinyl alcohol (PVA) film (Fig 1). In Segment A, the single exposure dose was 1 film. In Segment B, 7 daily doses were given of either the Active MB66 film or a Placebo control film containing the same excipients as the Active film.

**Fig 2:**
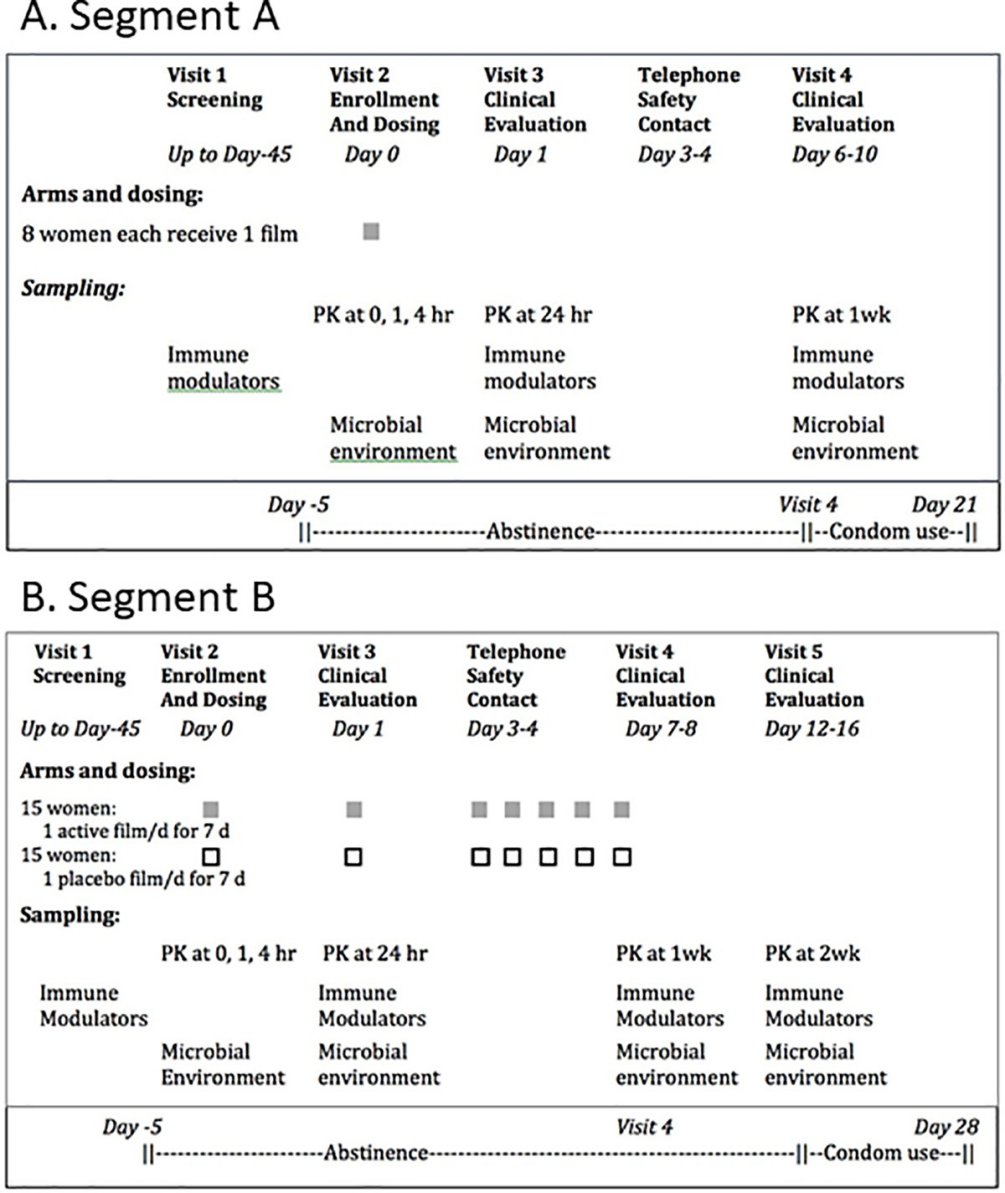
MB66-01 study Segments A and B schemata with target enrollment.

Segment A consisted of 4 visits, and Segment B consisted of 5 visits, both with a Telephone Safety Contact between Visits 3 and 4 (summarized in Fig 2). Visit 1 was the Screening Visit for each Segment. Women were administered a questionnaire covering a variety of variables related to study participation, including demographics, medical history, and eligibility. Prospective participants were also evaluated via laboratory tests (see S3 Table) and physical and pelvic examinations. A cervicovaginal lavage sample (CVL) was collected with speculum in place, using 5 mL of Dulbecco’s phosphate buffered saline dispensed 3 times through a syringe to wash the vaginal walls and ectocervix, then collected from the posterior fornix. This CVL served as the baseline sample for secondary and exploratory endpoints. During the Screening Visit, women agreed to sexual abstinence for 5 days before the Enrollment Visit through 1 week after enrollment and to use condoms for vaginal intercourse for an additional 2 weeks after the Exit Visit (Visit 4).

Participants who met the inclusion and exclusion criteria were scheduled for an Enrollment Visit (Visit 2). The Enrollment Visit was timed to occur within 2 weeks after the last menstrual period to ensure that menstruation would not occur during any of the study visits. The participant underwent a brief physical exam and a pelvic examination, vaginal pH measurement, pregnancy test, and urinalysis. If no bleeding or other exclusionary findings were evident, the participant was enrolled. In Segment A, the study clinician digitally inserted the study film into the vagina and recorded the time of insertion. In Segment B, the participant was randomized to study product, underwent a standardized training on study product application, and inserted her first dose of study film vaginally in the presence of the study clinician. At 1, 4, and 24 hours after initial film insertion, the study clinician inserted a speculum and scored the degree of film hydration (conversion from solid film to softened gel-like consistency) and subsequent complete disappearance (dissolution). Estimates were made of the degree of gel dissolution utilizing a scale ranging from 100% (total dissolution) to 0% (no evident dissolution).

Visit 3 (Follow-up Visit—Day 1) occurred on the next day. Segment B participants were given 8 doses (including 2 extra doses) of the study product in MEMS Cap (adherence monitoring) vials (AARDEX Group, Liège, Belgium) to be administered at home.

From Visit 3 to the end of the study, safety endpoints and adverse events (AEs) were assessed in participants of both segments. In addition, urinalyses, film dissolution estimates and vaginal pH measurements, CVLs, and other laboratory evaluations were obtained (see S1–S3 Tables). Blood and vaginal filter paper strip absorbed samples (TearFlo, Beaver-Visitec International, Waltham, Massachusetts, USA) were obtained to assess mAb levels and their distribution within the vaginal fluid over time; sampling at the cervical os, ectocervix, and mid and distal vagina. Bacterial morphotypes were assessed at all visits by Nugent scoring of Gram-stained vaginal smears [28]. In addition, Segment B participants completed a behavioral assessment capturing film acceptability, willingness-to-use (WTU), and sensory perception data following final film use (Visit 4).

The primary objective of the clinical trial was to evaluate the safety of the MB66 film by recording the incidence of Grade 2 or higher AEs deemed related to study product (Current Versions of the DAIDS Female Genital Grading Table for Use in Microbicide Studies and Table for Grading the Severity of Adult and Pediatric Adverse Events [29,30]). Safety was assessed throughout the study by monitoring AEs, vital signs and clinical laboratory values, performance of physical examinations, and review of concomitant medications and procedures. All AEs were reviewed by the Protocol Safety Review Team and analyzed regardless of severity or relationship to study products.

Secondary objectives included: rate of MB66 film dissolution, vaginal concentrations of MB66 antibodies, and blood concentrations of MB66 antibodies.

Exploratory objectives included: ex vivo antiviral effects of MB66 antibodies in CVL fluid from participants after dosing with MB66 film, effects of MB66 on the cervicovaginal microbial environment as determined by Nugent scoring of Gram-stained vaginal smears, effects of MB66 film on vaginal inflammation and innate immunity by measuring levels of proinflammatory cytokines and immune mediators in CVLs before and after film use, and acceptability of MB66 vaginal film after 7 days of use.

#### HIV-1 neutralization test

A standard TZM-bl neutralization protocol [31] was used to assess neutralization of 3 different strains of HIV-1: Q23-17 (primary clade A isolate, R5, Tier 2), BaL (laboratory-adapted clade B strain, R5, Tier 1), and LAI (laboratory-adapted clade B strain, X4, Tier 1). The viral stocks were kindly provided by Dr. Manish Sagar, Boston University School of Medicine. CVL samples, collected at baseline and various time points after use of Active or Placebo film, were used for the neutralization assays; tests were performed in triplicate. Individual HIV-1 strains (500 IP in 25 μL) were mixed with 25 μL CVL sample and incubated for 1 hour. Samples were then added to 1 × 10^4^ TZM-bl cells (NIH AIDS Reagent Program, Germantown, Maryland, USA) that were suspended in 50 μL of tissue culture medium in 96-well plates. The infection was allowed to proceed for 48 hours, and the samples were processed with β-galactosidase assay (Galacto-Star, Thermo Fisher Scientific, Bedford, Massachusetts, USA). Luminescence in RLU was recorded on a microplate reader (Synergy HTX, BioTek, Winooski, Vermont, USA). Infected TZM-bl cells (treated with virus only) served as positive controls, and uninfected TZM-bl cells (no virus) served as negative controls.

#### HSV-2 neutralization assay

For HSV-2 neutralization, the HSV-2 Plaque Reduction Neutralization Test was utilized [32]. Vero (African Green Monkey kidney) cells (ATCC, Manassas, Virginia, USA) were seeded in a 96-well plate at 5,000 cells/well and incubated for 18 to 24 hours at 37°C until 75% to 80% confluent. HSV-2 strain G (ATCC; 1000 TCID_50_ in 25 μL of DMEM) was incubated with 25 μL of CVL sample for 1 hour; each test was performed in triplicate. The virus–antibody mixture was then added to the Vero cell cultures and incubated for 12 to 15 hours at 37°C. The cells were then washed to remove excess virus and were further incubated for 4 days. Plaques were counted using an inverted phase contrast microscope. Infected Vero cells (virus only) served as positive controls, while uninfected Vero cells (no virus) served as negative controls.

#### Assessment of cytokines

Aliquots of CVLs from various study visits in Segments A and B (see Fig 2 for exact time points) were assayed by Luminex (Bio-Plex MAGPIX, Bio-Rad Laboratories, Hercules, California, USA) for cytokines that have been associated with HIV transmission [33–38], utilizing a custom Invitrogen ProcartaPlex multiplex kit (ThermoFisher, Waltham, Massachusetts, USA) per manufacturer’s protocol. The analytes included: TNF-1α, IL-6, IL-1α, IL-1β, IL-1RA, MIP-1α, MIP-1β, RANTES, SDF-1α, IL-8, MCP-1, IP-10, GM-CSF, IFN-γ, IL-10, and IL-12p40.

#### Monoclonal antibody levels

VRC01-N concentrations in CVL and TearFlo samples (Fig 2) were measured by validated electrochemiluminescence immunoassay. HSV8-N concentrations in CVL and TearFlo samples were measured by colorimetric ELISA.

#### Perceptibility and willingness-to-use

Segment B participants completed a behavioral assessment via audio computer-assisted self-interview format (ACASI) capturing film acceptability, WTU, and user sensory perception and experience (USPE) data. WTU items assessed probabilities of future use in single- and multipurpose indications. USPE scales captured specific sensory experiences elicited by film use. Only fully evaluable participants (*n* = 24) were included in this assessment because they had completed the study, and thus had had full exposure to the study product.

#### Data analysis

Participants’ data were entered in a database (StudyTRAX, Macon, Georgia, USA) on a password-protected computer. Discrete variables were analyzed by Fisher exact tests. Most continuous variables were analyzed by repeated measures analysis of variance (ANOVA) with the Geisser and Greenhouse correction to control for deviations in sphericity or by mixed linear model analysis. A significant effect was followed by Tukey (for ANOVA) or Tukey–Kramer (for mixed linear model) multiple comparison tests. If continuous variables were not normally distributed, they were log (natural) transformed prior to analysis. Unpaired *t* tests or Mann–Whitney *U* tests were performed for 2-group comparisons of some continuous variables. Spearman rank correlation coefficients were performed to evaluate the relationships between variables. Statistical significance was assumed when *p* < 0.05. Analyses were performed with SAS (Version 9.4), JMP Pro (Version 14.1.0) (both from SAS Institute, Cary, North Carolina, USA), and Prism (Version 8.3.1; GraphPad Software, La Jolla, California, USA). With respect to WTU and USPE data, response means were calculated for each evaluable group. To understand USPE differences between groups, we conducted comparisons on USPE averaged mean item scale scores to determine Hedges *g* effect size differences between groups [39].

## Results

### Cohort characteristics and primary endpoints

For the Primary endpoint (Grade 2 or higher AE deemed related to study product), all enrolled participants were included in the analysis (intention to treat) because they were all exposed to at least 1 film, and it was possible that an AE could have been responsible for a participant withdrawing early from the study. For the Secondary and Exploratory Endpoints (i.e., antibody concentrations, cytokines, ex vivo viral neutralization, etc.), only fully evaluable participants were included in the analyses. Fully evaluable Segment A participants were defined as those who completed the Screening and Enrollment Visits and returned for the Day 1 visit. Fully evaluable Segment B participants were defined as those who completed the Screening and Enrollment Visits, returned for Visit 4, and were found by MEMS recordings to have used study product for at least 5 of the previous 7 days including the day before presentation.

### Segment A

#### General information

**Fig 3:**
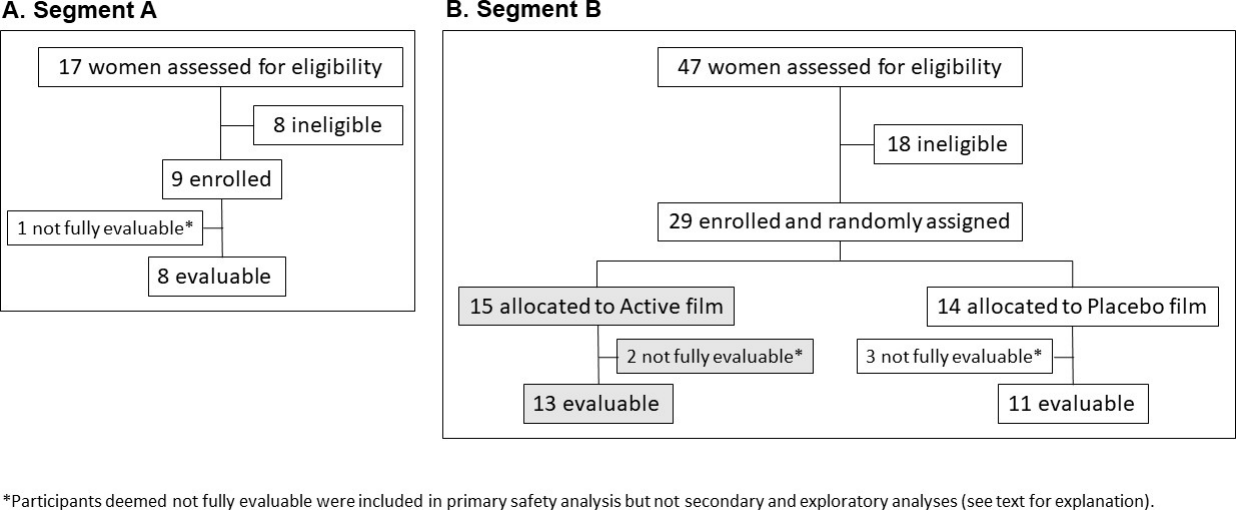
Diagram of MB66-01 enrollment.

In Segment A, 17 women were screened to enroll 9 participants, 8 of whom completed the study (fully evaluable). In Segment A, ineligibility following screening (*n* = 8) was due to: enrollment period lapse (*n* = 2), abnormal alanine aminotransferase test (*n* = 1), latex allergy (*n* = 1), abnormal urinalysis (*n* = 1), inadequate birth control/positive Trichomonas test (*n* = 1), abnormal Pap test (*n* = 1), and positive Trichomonas test/abnormal urinalysis (*n* = 1). See Table 1 for baseline characteristics and Fig 3A for enrollment breakdown.

**Table 1:** Summary of baseline characteristics of enrolled participants.

Demographic	Subcategory	Segment A	Segment B	
		Active Film (*n* = 9)	Active Film (*n* = 15)	Placebo Film (*n* = 14)
**Race**^**1**^	*White*	5 (45.5%)	11 (73.3%)	10 (71.4%)
	*African-American*	4 (36.4%)	3 (20.0%)	3 (21.4%)
	*Native American/ Alaska Native*	1 (9.1%)	2 (13.3%)	0
	*Asian*	1 (9.1%)	0	0
	*Native Hawaiian/Pacific Islander*	0	0	0
	*Other/Unknown*	0	1 (6.7%)	1 (7.1%)
**Ethnicity**	*Latino/Hispanic*	2 (22.2%)	2 (13.3%)	3 (21.4%)
	*Other*	7 (77.8%)	13 (86.7%)	11 (78.6%)
**Age** (no. in range)	*18–21*	0	2 (13.3%)	3 (21.4%)
	*22–30*	3 (33.3%)	9 (60.0%)	7 (50.0%)
	*31–40*	5 (55.6%)	3 (20.0%)	3 (21.4%)
	*41–45*	1 (11.1%)	1 (6.7%)	1 (7.1%)
**Age** (Mean ± SD)		32.1 ± 6.5	28.0 ± 6.9	26.1 ± 7.1
**Height (cm.)** (Mean ± SD)		162.3 ± 9.9	162.3 ± 7.6	164.3 ± 5.3
**Weight (kg.)** (Mean ± SD)		93.4 ± 32.8	78.0 ± 20.6	76.6 ± 12.1
**Contraception**^**1**^	*Hormone-based*	2 (22.2%)	6 (40.0%)	9 (64.3%)
	*IUD (hormonal)*	2 (22.2%)	4 (26.7%)	3 (21.4%)
	*IUD (copper)*	0	1 (6.7%)	0
	*Male Condoms*	0	1 (6.7%)	2 (14.3%)
	*Vasectomy*	1 (11.1%)	0	0
	*Sterilization*	1 (11.1%)	1 (6.7%)	0
	*Female Partner Only*	2 (22.2%)	0	0
	*Abstinence (at least 60 days)*	1 (11.1%)	3 (20.0%)	2 (14.3%)

^1^Totals sum to > 100% because more than 1 subcategory was allowed.

#### Adverse events

In Segment A, there were 8 AEs reported in 3 of the 9 participants (See Table 2, S4 and S6 Tables for details). All but one AE had a severity grading of 1; a vaginal itching AE had a severity grading of 2. Three grade 1 and no grade 2 or higher AEs were classified as related to study product. Following an assessment by the Protocol Safety Review Team, MB66-01 was allowed to proceed to Segment B.

**Table 2:** Distribution and nature of adverse events by study arm.

	Segment A		Segment B					
	Active Film		Active Film		Placebo Film		Active vs. Placebo	
	(*n* = 9)		(*n* = 15)		(*n* = 14)		*p*-value	
	Total	Related^1^	Total	Related	Total	Related	Total	Related
**No. of AEs**	8	3	27	10	18	9	-----	-----
**No. of AEs per Participant**	0.89 ± 0.48^**2**^	0.33 ± 0.24	1.80 ± 0.34	0.67 ± 0.23	1.29 ± 0.24	0.64 ± 0.20	0.29^**3**^	0.95
**Maximum AE Grade per Participant**	0.44 ± 0.24	0.22 ± 0.15	1.13 ± 0.17	0.60 ± 0.19	1.14 ± 0.21	0.57 ± 0.17	0.96	0.99
**No. of Participants with AE**	3 (33.3%)^**4**^	2 (22.2%)	13 (86.7%)	7 (46.7%)	12 (85.7%)	7 (50.0%)	1.00^**5**^	1.00
**AEs ≥ Grade 2**	1 (11.1%)	0	4 (26.7%)	2 (13.3%)	4 (28.6%)	1 (7.1%)	1.00	1.00
**AE Category**^**6**^								
**Genitourinary**								
UTI	0	0	1 (6.7%)	0	1 (7.1%)	1 (7.1%)	1.00	0.48
Uterine Cramping	1 (11.1%)	0	2 (13.3%)	2 (13.3%)	0	0	0.48	0.48
Vaginal Spotting	1 (11.1%)	1 (11.1%)	2 (13.3%)	1 (6.7%)	4 (28.6%)	2 (14.3%)	0.39	0.60
Vaginal Discharge	0	0	2 (13.3%)	2 (13.3%)	2 (14.3%)	2 (14.3%)	1.00	1.00
Vaginal Itching	1 (11.1%)	0	0	0	1 (7.1%)	1 (7.1%)	0.48	0.48
Vaginal Odor	0	0	3 (20%)	2 (13.3%)	1 (7.1%)	1 (7.1%)	0.60	1.00
Labial Abrasions	1 (11.1%)	0	0	0	0	0	1.00	1.00
Asymptomatic Microscopic Hematuria	1 (11.1%)	1 (11.1%)	7 (46.7%)	2 (13.3%)	4 (28.6%)	1 (7.1%)	0.45	1.00
Proteinuria	1 (11.1%)	1 (11.1%)	2 (13.3%)	1 (6.7%)	3 (21.4%)	1 (7.1%)	0.65	1.00
**Other Clinical**								
Cold Symptoms	0	0	1 (6.7%)	0	0	0	1.00	1.00
Sinus Congestion	0	0	1 (6.7%)	0	0	0	1.00	1.00
Diarrhea	0	0	0	0	1 (7.1%)	0	0.48	1.00
Fainting	0	0	1 (6.7%)	0	0	0	1.00	1.00
Breast Tenderness	0	0	1 (6.7%)	0	0	0	1.00	1.00
Chest Rash	1 (11.1%)	0	0	0	0	0	1.00	1.00

^**1**^Related to study product.

^**2**^Mean ± SE.

^**3**^Mann–Whitney *U* test.

^**4**^Frequency (%).

^**5**^Fisher exact test.

^**6**^Each participant contributed only 1 observation per category.

### Segment B

#### General information

In Segment B, 47 women were screened to enroll 29 participants who were randomly assigned to either Active (*n* = 15) or Placebo (*n* = 14) film groups. For this segment ineligibility following screening (*n* = 18) was due to: enrollment period lapse (*n* = 6), positive Trichomonas test (*n* = 2), no Pap test available (*n* = 2), unallowable medications (*n* = 4), positive pregnancy test (*n* = 1), irregular menses (*n* = 1), age (*n* = 1), and not medically stable (*n* = 1). Of the enrolled women, 5 were not fully evaluable due to incomplete film usage as a result of AEs (3 women) or nonadherence to film insertion schedule (2 women; as determined by MEMS Cap records). Of the 3 women who were not fully evaluable due to AEs, only 1 was in the Active film group. In addition, only 1 of the AEs in these 3 women was determined to be related to product and occurred in the Placebo film group. This left 13 fully evaluable women in the Active film group and 11 in the Placebo film group. See Fig 3B for enrollment breakdown and Table 1 for baseline characteristics. Demographics matched those of the patient population at Miriam Hospital and did not differ between Active and Placebo film groups.

#### Adverse events

In Segment B, 45 AEs were reported; 27 in the Active film group and 18 in the Placebo film group. The incidence of AEs ≥ Grade 2 deemed related to study product did not differ significantly between Active and Placebo groups [2/15 (13.3%) versus 1/14 (7.1%), *p* = 1.00]. An extensive comparison of AEs in Active and Placebo film groups revealed no significant differences between the groups (Table 2, S5 and S6 Tables).

### Secondary and exploratory variables

#### MB66 film dissolution

S1 Fig shows MB66 film dissolution at various time points following insertion of a single film in Segment A and insertion of 1 and 7 films in Segment B. All study films were fully hydrated and had transformed into a gel form 1 hour after insertion. In Segment A, substantial (>50%) film dissolution was observed in all participants at the 1-hour post film insertion time point (S1A Fig). In Segment B, MB66 and Placebo films were largely dissolved by 4 hours after insertion. There were no significant differences in dissolution rates between Active and Placebo film groups or after insertion of 1 versus 7 films (S1B Fig).

#### Vaginal pH

In Segment A, there were no significant differences in vaginal pH at Day 0 (prior to MB66 film insertion) compared to 24 hours and 7 days following insertion of a single film (S2A Fig). Similarly, in Segment B, there were no significant differences in vaginal pH across visits (following initial and repeated film insertions) or between Active and Placebo film groups (S2B Fig).

#### Nugent scores

In Segment A, there were no significant differences in Nugent scores at Day 0 (prior to MB66 film insertion) compared to 24 hours and 7 days following insertion of a single film (S3A Fig). Similarly, in Segment B, there were no significant differences in Nugent scores across visits (following initial and repeated film insertions) or between Active and Placebo film groups (S3B Fig).

#### Pharmacokinetics of mAb concentrations in vaginal secretions

**Fig 4:**
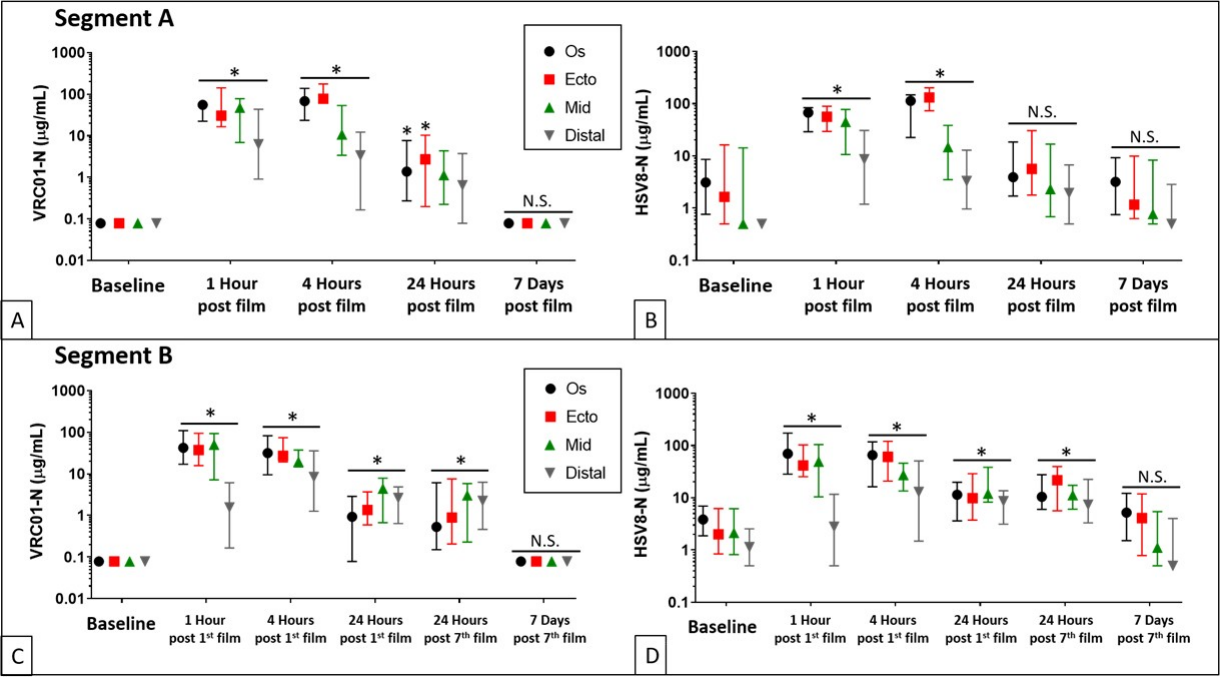
VRC01-N and HSV8-N concentrations (represented by medians with interquartile ranges) in cervicovaginal TearFlo samples in Segments A and B (Active film group only). (**A).** Segment A, VRC01-N: All 4 sites significantly elevated at 1 hour (*p* < 0.001 for all site comparisons) and 4 hours (*p* < 0.001 for cervical os, ectocervix, and mid vagina sites; *p* = 0.007 for distal vagina) post single film insertion compared to respective baseline concentrations. Cervical os (*p* = 0.046) and ectocervix (*p* = 0.02) were elevated 24 hours post film insertion. Mid (*p* = 0.09) and distal vagina (*p* = 0.54) were not significantly elevated 24 hours post film insertion. (**B).** Segment A, HSV8-N: All sites were significantly elevated at 1 hour and 4 hours post single film insertion (*p* < 0.001 for all comparisons) but not at the 24-hour time point. (**C).** Segment B, VRC01-N: All 4 sites were significantly elevated at 1 (*p* < 0.001 for all site comparisons) and 4 (*p* < 0.001 for all site comparisons) hours following first film insertion compared to each site’s respective baseline concentration. Concentrations at all 4 sites 24 hours after first film and 24 hours after seventh film remained significantly elevated compared to Baseline (*p* < 0.001 for distal vagina, ectocervix, and mid vagina sites and *p* = 0.01 for cervical os for Baseline vs. 24 hours post first film, and *p* < 0.001 for distal vagina, ectocervix, and mid vagina sites and *p* = 0.0007 for cervical os for Baseline vs. 24 post seventh film). (**D).** Segment B, HSV8-N: All 4 sites were significantly elevated at 1 and 4 hours following first film insertion compared to Baseline concentrations. Concentrations at all 4 sites at 24 hours after first film and 24 hours after seventh film remained significantly elevated compared to Baseline (*p* < 0.001 for all comparisons).

*VRC01-N*: In Segment A, VRC01-N concentrations in TearFlo samples were significantly elevated at all 4 cervicovaginal sites at 1 hour and 4 hours post single film insertion compared to baseline. MAb concentrations in cervical os and ectocervix samples remained significantly elevated 24 hours post film insertion, whereas concentrations in mid and distal vagina samples were not significantly elevated 24 hours post film insertion (Fig 4A). In Segment B, 96 Placebo film TearFlo samples from 8 participants (4 vaginal sites; baseline, 1-, 4-, and 24-hour time points) were all below detection for VRC01-N. For the Active film group, mAb concentrations at all 4 vaginal sites were significantly elevated at 1 and 4 hours following first film insertion compared to baseline (*p* < 0.001). Although concentrations at all 4 sites were decreased from peak values at 24 hours after film insertion, they remained significantly elevated compared to Baseline (Fig 4C).

*HSV8-N*: In Segment A, all sites were significantly elevated for HSV8-N at 1 hour and 4 hours post single film insertion (*p* < 0.001 for all comparisons) but not at the 24-hour time point (Fig 4B). In Segment B, both the Active and Placebo film samples were included in the statistical analysis of HSV8-N TearFlo sample concentrations. For the Active film group, mAb concentrations were significantly elevated at all 4 cervicovaginal sites at 1 and 4 hours following first film insertion compared to Baseline concentrations (*p* < 0.001 for all comparisons). As in the VRC01-N analysis, although concentrations at all 4 sites decreased at the 24-hour time point after first film and seventh film use, they remained significantly elevated compared to Baseline (*p* < 0.001 for all comparisons; Fig 4D). In addition, all post-film time points, except for 7 days after seventh film, were significantly higher for Active film compared to Placebo film counterparts for all 4 sites (*p* < 0.01 for all comparisons). The Baseline samples did not differ significantly between the Active and Placebo film groups.

#### Relationship between film dissolution and mAb pharmacokinetics

Because film dissolution was incomplete at 1 hour in a number of participants in Segment B, we examined the relationship between film dissolution and antibody concentrations. Therefore, we performed Spearman rank correlation coefficients between 1-hour film dissolution (%) and both VRC01-N and HSV8-N concentrations at 1 hour in the 4 TearFlo cervicovaginal sites in the Segment B participants. All of the correlation coefficients between film dissolution and antibody concentrations were <0.30 with *p*-values > 0.35. Therefore, in the Segment B samples, there was no detectable relationship between film dissolution and antibody concentration.

#### Pharmacokinetics of mAb concentrations in serum

Serum VRC01-N concentrations in Segment A (Visits 1, 3, and 4) and Segment B (Visits 1, 3, 4, and 5 for Active film participants) were all below the lower limit of quantitation of the assay (25 ng/mL).

#### Studies with CVL samples

CVL samples were collected at specific time points (baseline, 24 hours, and 7 days after film insertion) to provide sufficient material for mAb concentrations, virus neutralization assays, and assessment of proinflammatory cytokines and chemokines.

#### Assessment of mAb concentration in CVLs

**Fig 5:**
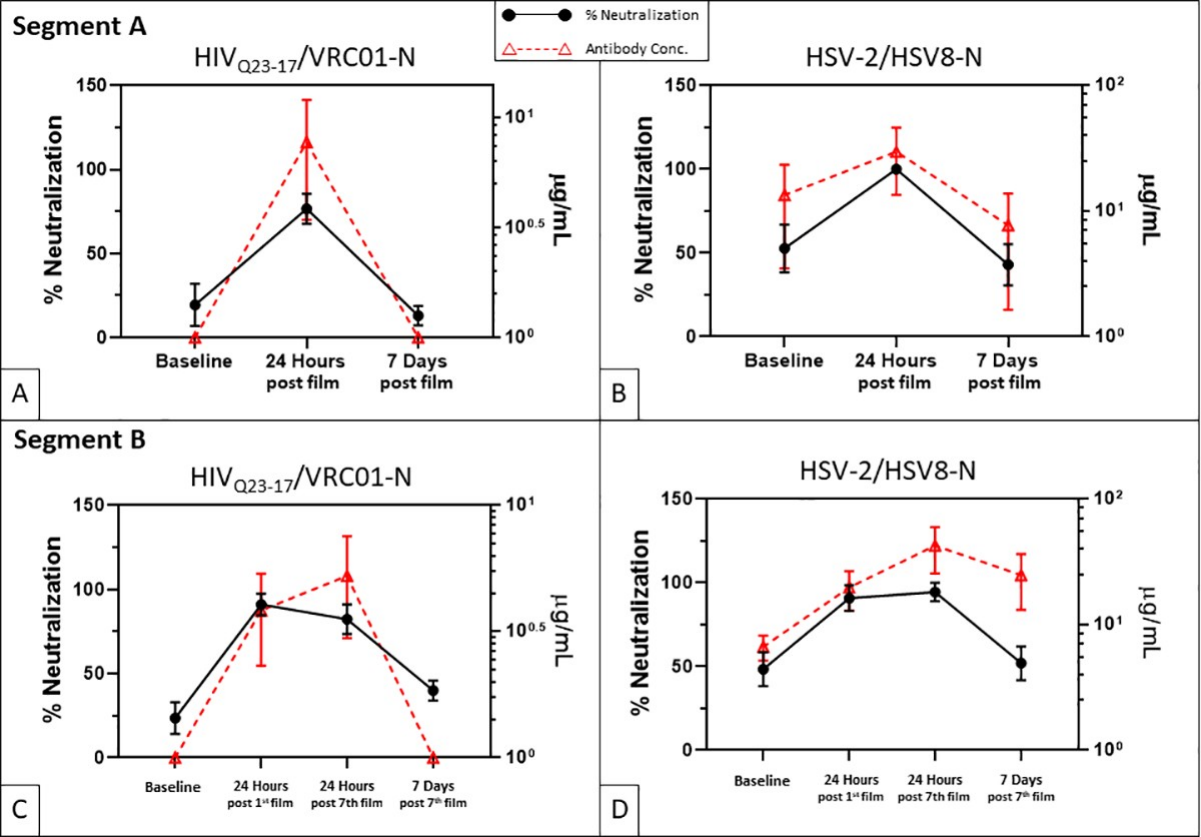
Viral neutralization and antibody pharmacokinetics (represented by means ± SEMs) in CVLs in Segments A and B (Active film group only). Neutralization percent is represented by solid lines/circles (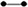) (left *y* axis), and antibody concentration is represented by dashed lines/open triangles (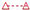) (right *y* axis) in each graph. (**A).** VRC01-N concentration and HIV_Q23-17_ neutralization in Segment A were significantly increased at 24 hours post single film insertion compared to both Baseline (*p* = 0.006 and 0.01, respectively) and 7 days post single film insertion (*p* = 0.006 and 0.02, respectively). (**B).** HSV8-N concentrations in Segment A were significantly increased at 24 hours post single film insertion compared to both Baseline (*p* = 0.04) and 7 days post single film insertion (*p* = 0.01). All CVL samples from 24 hours post film insertion exhibited 100% HSV neutralization; however, the omnibus F test *p*-value did not reach statistical significance (*p* = 0.052). (**C).** VRC01-N concentration and HIV_Q23-17_ neutralization in Segment B were significantly elevated 24 hours after first film and 24 hours after seventh film compared to Baseline (*p* < 0.001 for all comparisons). (**D).** In Segment B, HSV8-N concentrations 24 hours after first film and 24 hours after seventh film did not differ significantly from Baseline (*p* = 0.056, Group x Visit interaction), but HSV neutralization was significantly increased at 24 hours post first film (*p* = 0.003) and 24 hours post seventh film compared to Baseline (*p* = 0.001).

In Segment A, VRC01-N concentrations in CVLs were significantly increased at 24 hours post single film insertion compared to the Baseline visit 1 (*p* = 0.006), but at the 7-day post film insertion time point, mAb concentrations had returned to baseline (Fig 5A). In Segment B, CVL samples from 8 participants that received Placebo film did not contain detectable levels of VRC01-N at any time point. For the Active film group, VRC01-N concentrations in CVLs were significantly elevated 24 hours after first film and seventh film insertion time points compared to Baseline *p* < 0.001); at the 7-day post seventh film time point, VRC01-N levels had returned to Baseline (Fig 5C).

A similar pattern was observed for HSV8-N concentrations. In Segment A, HSV8-N concentrations in CVLs were significantly increased 24 hours post single film insertion compared to Baseline (Visit 1) (*p* = 0.04) but were not significantly different from Baseline at the 7-day post film time point (*p* = 0.64) (Fig 5B). In Segment B, both the Active and Placebo film samples were included in the statistical analysis of HSV8-N CVL concentrations since some of the Baseline and Placebo film CVLs had detectable HSV titers, probably due to the presence of natural anti-HSV antibodies in HSV-infected women. Antibody concentrations in CVL samples from Active film participants collected 24 hours after first and seventh MB66 film use were elevated but fell slightly short of statistical significance compared to Baseline and Placebo samples (Group x Visit interaction, *p* = 0.056) (Fig 5D).

#### HIV neutralization

In Segment A, CVLs collected from women 24 hours after single film insertion significantly neutralized the Tier 2 primary HIV isolate HIV_Q23-17_ [significantly different from Baseline (*p* = 0.01)]; however, neutralizing activity was no longer detectable by 7 days post film insertion [*p* = 0.99; Fig 5A]. CVL neutralization of 2 laboratory-adapted Tier 1 HIV strains, HIV_BaL_ and HIV_LAI_, exhibited the same pattern (S4 Fig). Similarly, in Segment B, CVLs from the Active film group collected 24 hours after insertion of 1 or multiple films significantly neutralized HIV_Q23-17_ (*p* < 0.001 for both compared to baseline), but CVLs collected 7 days after insertion of single or multiple films did not neutralize (Fig 5C). Similar neutralization patterns were observed for HIV_BaL_ and HIV_LAI_ (S5 Fig). HIV neutralization correlated with HIV mAb concentration (Fig 5).

#### HSV-2 neutralization

In Segment A, all CVL samples collected 24 hours after insertion of a single film exhibited 100% neutralization of HSV-2; however, the omnibus F test *p*-value for the ANOVA for HSV-2 neutralization did not quite reach significance (*p* = 0.052) due to small sample size and neutralization activity in some of the baseline samples (Fig 5B and S4 Fig). In Segment B, CVLs from the Active film group significantly neutralized HSV-2 at 24 hours post first film and post seventh film [p = 0.004 and p = 0.001 compared to Baseline, and p = 0.001 and 0.0007 compared to Placebo (S5 Fig)]. CVLs collected 7 days after daily insertion of 7 films did not neutralize (p = 0.1; Fig 5D and S5 Fig).

#### Immune mediators/cytokines

**Fig 6:**
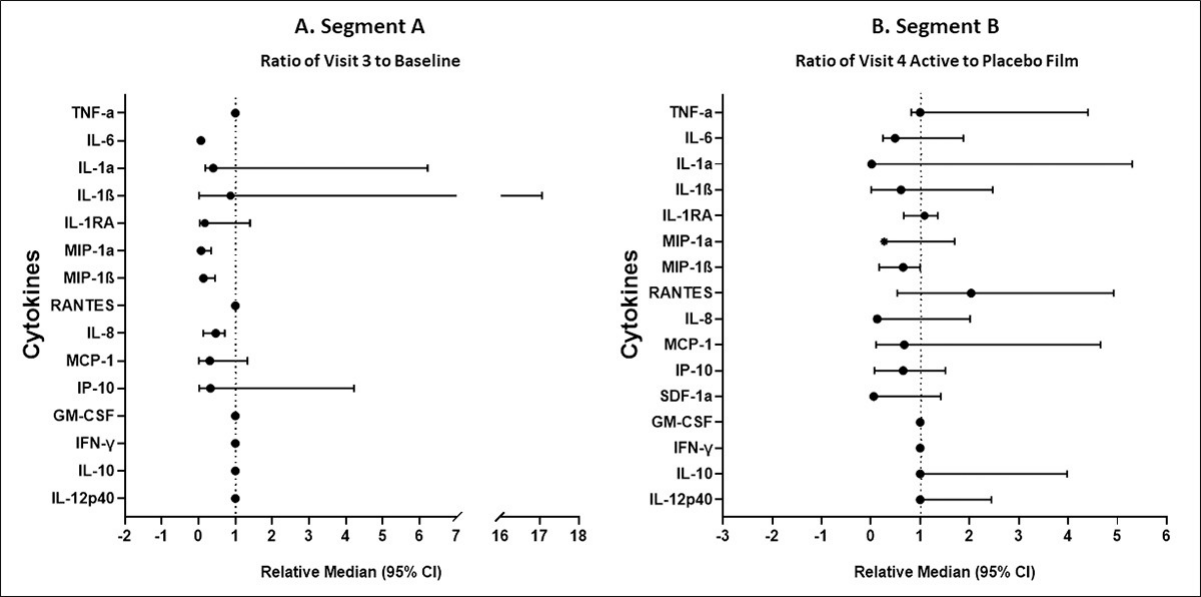
Forest plots of cytokines in CVLs: (**A).** The ratio (relative median ± 95% confidence interval) of Visit 3 (24 hours after single film) to Baseline concentrations in Segment A, and (**B).** The ratio (relative median ± 95% confidence interval) of Active film Visit 4 (24 hours after seventh film) to Placebo film Visit 4 concentrations in Segment B. No significant increases in cytokine concentrations were observed following Active film insertion or in Active film vs. Placebo film group comparisons.

In Segment A, levels of proinflammatory cytokines associated with HIV transmission were not significantly increased in CVLs 24 hours following single film insertion (Fig 6A). Specifically, there were no statistically significant differences in concentrations of 11 of 15 cytokines measured, including the key proinflammatory cytokines, IL-1β, TNF-α, IL-8, MCP-1, and IP-10 across all 3 visits [Baseline (predose), 24-hour, and 7-day post single film time points]. Interestingly, concentrations of IL-1α, IL-6, MIP-1α, and MIP-1β were significantly reduced at least 1 time point after film use compared to Baseline. The ratio of IL-1RA to IL-1, an adjusted anti-inflammatory measure [40–43], was significantly higher 7 days post film use compared to Baseline (*p* = 0.002). Concentrations of IL-12p40, IFN-γ, and RANTES were below detection for all Segment A CVL samples.

For Segment B, the Group main effect (i.e., Active versus Placebo film) was not significant for any CVL cytokine variable. Fig 6B summarizes the comparison of CVL cytokine variables for Active and Placebo films 24 hours after 7 daily exposures to film. Most cytokines (IL-10, IL-12p40, IFN-γ, MIP-1α, MIP-1β, GM-CSF, and SDF-1α) did not exhibit either Group or Visit main effects, but a subset of cytokines exhibited significant Visit effects; for the most part, these were the result of reductions in concentrations compared to Baseline, as had been observed for Segment A, and usually occurred across both Active and Placebo film groups (S6 and S7 Figs). Levels of IL-1RA and the IL-1RA/IL-1 ratio, both markers of anti-inflammatory effects, were significantly elevated in both Active and Placebo film groups 24 hours after daily use of 7 films compared to Baseline (S6 Fig).

#### Willingness-to-use and perceptibility

Overall, the results from the Placebo and Active film groups were similar with respect to willingness-to-use. Three women each from the Placebo and Active groups responded that they would “probably” use the study film, while 8 and 10 women, respectively, responded that they would “definitely” use the film. With respect to perceptibility, only 1 statistically significant difference between evaluable data was noted: the *AMB (ambulation)*: *Hygiene* scale. Lack of statistical significance is not surprising, as we expected low power with small sample sizes compared as independent groups. That said, there were notable effect sizes in USPE scale score comparisons between groups, many in the medium to large range (Hedges *d* of 0.2, 0.5, and 0.8 are considered small, medium, and large effects, respectively). These results can be indicative of important differences between groups and should be reassessed in larger samples. These are reported in Table 3. In sum and with deference to the effect size only, the Placebo film appears to have resulted in greater endorsement of leakage and hygiene concerns, while the Active film was somewhat easier to insert and more noticeable to the user during daily activities.

**Table 3:** Group comparisons with averaged scale item scores, standard deviations, mean differences, confidence intervals, and effect sizes for application (APP) and ambulation (AMB) scales removing nonevaluable cases.

Perceptibility (USPE^a^) Scale	Placebo ASIS (*sd*)^b^ (*n* = 11)	Active ASIS (*sd*)^b^ (*n* = 13)	Mean Difference^*c*^ (95% CI)	*p*-value	Effect Size Hedges *g* (95% CI)
*APP*^*d*^: *Leakage* (Perceptions and experiences of leakage immediately upon insertion)	1.45 (0.72)	1.18 (0.30)	.27 (−0.23, 0.78**)**	0.256^e^	.49 (−0.33, 1.30)
*APP*: *Ease* (Physical comfort, simplicity, and ease of insertion process)	3.33 (0.73)	3.83 (0.85)	.50 (−0.17, 1.18)	0.138	.61 (−0.22, 1.43)
*APP*: *Discreet-Portable* (Users’ perceptions of the product’s ability to be used and carried discreetly)	4.09 (0.99)	4.15 (0.68)	.06 (−0.64, 0.77)	0.855	.07 (−0.73, 0.83)
*APP*: *Product Awareness* (Physical sensations of the product in the vagina during and after insertion)	2.04 (0.87)	2.12 (0.83)	.09 (−0.63, 0.81)	0.805	.09 (−0.71, .89)
*AM*^*f*^: *Product Movement* (Sensations of the product moving/flowing from fornix toward introitus during ambulation)	1.42 (0.78)	1.26 (0.28)	.15 (−0.33, 0.63)	0.520	.27 (−0.53, 1.08)
*AMB*: *Leakage* (Sensations of the product leaking out of the vagina during ambulation; perceptions of messiness during ambulation; sensations of the product having moved/flowed to the introitus)	1.53 (0.70)	1.32 (0.34)	.21 (−0.24, 0.66)	0.347	.38 (−0.43, 1.19)
*AMB*: *Hygiene* (Sensations of leakage during daily activity; perceptions of desire to engage in hygiene (wiping) during daily activity)	2.09 (1.12)	1.38 (0.45)	.71 (0.01, 1.40)	0.048	.83 (0.01, 1.67)
*AMB*: *Stickiness* (Sensations of intra- and extravaginal stickiness during ambulation and daily activity)	1.58 (0.82)	1.44 (0.60)	.14 (−0.46, 0.74)	0.634	.19 (−0.61, 1.00)
*AMB*: *Product Awareness* (User perceptions that the product felt natural/comfortable as time passed postinsertion; physical sensations of the product in the vagina changing with time)	2.95 (0.85)	3.25 (0.85)	.30 (−0.43, 1.02)	0.405	.34 (−0.47, 1.15)
*AMB*: *Spreading Behavior* (Sensations and perceptions of the product distributing evenly in the vagina; sensations of smoothness, moisture, lubrication and mixing with natural lubrication)	3.04 (0.85)	2.68 (0.74)	.36 (−0.31, 1.03)	0.280	.44 (−0.37, 1.25)

^a^User sensory perception and experience: See Morrow et al [44] for detailed descriptions of scales.

^b^Averaged scale item score (standard deviation).

^c^Differences in the third decimal digits of the individual group means may cause the mean difference value to be off by 0.01 if calculating by hand.

^d^Application perceptibility scale.

^e^Robust test *p*-value reported because of homogeneity of variance violation.

^f^Ambulation perceptibility scale.

Note: Effect size column is the Hedges *g* effect size for the comparisons of placebo film to active film, where effect sizes of 0.2, 0.5, and 0.8 are considered, respectively, small, medium, and large. Given the small sample size (and lack of statistical power), the *g* effect size offers a more useful estimate of the potential difference between active and placebo films.

Likert Scale: 1 = Do Not Agree at All; 2 = Agree a Little; 3 = Agree Somewhat; 4 = Agree a Lot; 5 = Agree Completely.

## Discussion

The results of this Phase I safety trial of MB66 indicate that single and repeated vaginal applications of a film containing a combination of mAbs against different STI pathogens, which had been produced using a rapid cost-effective plant-based platform, were safe and well tolerated and achieved antibody concentrations in vaginal secretions capable of neutralizing HIV-1 and HSV-2 viruses for at least 24 hours after film application.

Two previous smaller studies assessed the safety of vaginal use of HIV mAbs in women. Ma and colleagues [20] tested the safety of the 2G12 HIV-neutralizing mAb produced in *N*. *benthamiana*, and Morris and colleagues [21] tested a gel (MABGEL) containing 3 HIV neutralizing mAbs, 2F5, 4E10, and 2G12, produced using Chinese Hamster ovary (CHO) cells. Both of these studies reported no serious AEs, and preliminary PK studies indicated that mAb concentrations peaked in vaginal secretions after 1 hour and were undetectable by 24 hours following the administration of up to 28 mg of mAb delivered in either saline or gel. Our study confirms and extends these findings but also differs in several significant aspects. Our study is the first-in-human safety trial of an antibody-based MPT product that contains mAbs against more than 1 STI pathogen. In addition to the clinical safety endpoints, we used vaginal secretions from study participants to show that the MB66 film did not incite a proinflammatory cytokine response related to HIV susceptibility. And even though we used a lower mAb dose than the other 2 studies (10 mg versus a maximum of 28 mg), we recorded significant mAb concentrations and antiviral neutralization activity in vaginal secretions 24 hours after film insertion (versus 12 hours in the other studies) possibly attributable to the film formulation. These findings recommend further development and testing of mAb-based MPT products.

There was a low incidence of Grade 2 or greater AEs determined to be related to study product in both Segment A (single film) and Segment B (multiple films), and the number of AEs did not differ between Active and Placebo film groups (Segment B). The most common AE noted in this study was asymptomatic microscopic hematuria. A total of 15 hematuria AEs was reported; 4 were classified as related to study product. Six of the cases occurred prior to film insertion and an additional 3 cases were related to menses. The laboratory test for hematuria was extremely sensitive, and the relatively high incidence of asymptomatic microscopic hematuria in this study was not associated with exposure to study product. Some of the episodes of microscopic hematuria may have been associated with trauma related to product insertion or pelvic procedures. Most of the participants with hematuria did not have other genitourinary abnormalities. In Segments A and B, there were no differences in vaginal pH or Nugent scores, or significant increases in levels of proinflammatory cytokines in CVLs 24 hours after Active film insertion. Likewise, in Segment B, there were no significant differences in any of these measures between Active and Placebo film groups after product use for 7 days.

Vaginal films were fully hydrated and showed substantial dissolution (disappearance) 1 hour post film insertion. Significant concentrations of VRC01-N and HSV8-N mAbs were detected in vaginal secretions following insertion of Active film. Antibody levels from filter paper samples peaked at 1 hour postdosing (median: 35 μg/mL) and remained significantly elevated at 24 hours post first film and seventh film (median: 1.8 μg/mL). Factoring in sample dilution (approximately 1:20), VRC01-N concentrations ranged from 36 to 700 μg/mL through the 24-hour time point, well above the IC_50_ for VRC01 (0.32 μg/mL) [45]; HSV8-N concentrations adjusted for dilution ranged from 80 to 601 μg/mL over the 24-hour collection period and remained above the IC_50_ for HSV8 (0.1 μg/mL) [46]. Film dissolution was incomplete at 1 hour in a number of participants in Segment B. However, there was no detectable relationship between film dissolution and antibody concentrations in vaginal secretions in these women. VRC01-N was undetectable in serum after film insertion indicating no evidence of systemic absorption of vaginally applied mAbs. This finding is consistent with that of Morris and colleagues [21] and Ma and colleagues [20] who reported no detectable systemic absorption of anti-HIV mAbs (2F5, 4E10, and 2G12) following vaginal administration. Low systemic absorption reduces the potential for immunogenicity or other systemic toxicities of vaginally administered mAbs.

Film dissolution did appear to be noticeably better 1 hour following single film insertion in Segment A than B. One explanation for this difference could be that the Segment A films were inserted by the study clinician, whereas Segment B films were inserted by the participants and may not have been placed as deeply. This difference did not appear to have an effect on safety or ex vivo efficacy measures but deserves attention in future studies.

CVL samples collected after MB66 film application were tested for antibody concentration and ex vivo efficacy in viral neutralization assays to determine whether the antibodies retained their activity in the vaginal environment. CVL samples collected 24 hours after single and multiple film insertion showed statistically significant neutralization of all 3 HIV-1 strains and of HSV-2 (Segment B only); however, viral neutralization was not observed at the 7-day time point, indicating that protection lasts for at least 24 hours but not 1 week after film insertion. In some individuals, modest neutralization of HIV and HSV observed in baseline CVL samples may be attributed to activation of endogenous vaginal defense mechanisms [47,48]. However, in general, there was more variability in HSV neutralization and HSV8-N concentrations probably due to endogenous anti-HSV antibodies in HSV-1 and 2 seropositive participants. While it may have been desirable to include only HSV-1 and 2 seronegative participants, it seems unlikely that confounding by HSV serostatus substantially influenced the current findings because HSV8-N concentrations and HSV-2 neutralization in vaginal secretions both increased following film insertion and fell again after washout. In fact, HSV-2 neutralization was observed in 100% of samples in Segment A following film insertion. In addition, differences in HSV-2 neutralization in Segment B were significant for comparisons to Baseline or Placebo. Finally, because the rate of HSV seropositivity is so high in US women (51% and 16% for HSV-1 and HSV-2, respectively), it can be argued that inclusion of such women in this Phase I study is warranted [49].

Insertion of MB66 Active film was not associated with an increase in proinflammatory cytokines or chemokines in CVLs. In fact, film insertion (both Active and Placebo) was associated with a reduction in the concentrations of a small subset of cytokines and chemokines. Because various microbicide gels have been shown to inhibit multiplex cytokine assays [33], we tested for MB66 film interference in the Luminex assay but did not observe a substantial inhibitory effect. Given that reductions in concentrations of some cytokines in CVLs have been observed after application of microbicide gels in other studies [33], it is possible that exposure to the MB66 film has an effect on the vaginal epithelium or microbiome; we are currently examining these possibilities.

Acceptability and willingness to use the product were judged to be high by post-use perceptibility and willingness-to-use surveys. With respect to perceptibility and, again, with deference to effect sizes rather than statistical significance in this small sample, there are user experiences to be considered moving forward. Given leakage and hygiene scores in this sample, it will be important to better characterize the processes and properties of dissolution and miscibility of the Active film and determine whether these impact user experience and willingness to use. It will also be important that the cumulative perceptibility effect of daily insertions of the film be monitored with respect to ongoing adherence in future clinical trials. Further, it was noted that women less than 30 years of age reported a lower score on the *APP*: *Ease* scale than those older, indicating lower endorsement of ease of insertion among these younger women. Given noted adherence differences in recent clinical trials among younger women, we believe learning insertion skills should be emphasized going forward [50–52].

The results of our study indicate that MB66 is safe and acceptable to women. Furthermore, ex vivo data demonstrated excellent antiviral protection for at least 24 hours after product insertion, providing evidence that MB66 is a promising MPT product to protect women against HIV and HSV-2. Limitations to our study include that samples were not collected between 24 hours and 7 days for pharmacokinetic evaluation, that women were not screened for HSV-1 and 2 antibodies prior to enrollment, and that variable film dissolution rates indicate that participants may require product insertion training. In addition, more clinical trials are needed to ascertain clinical efficacy and acceptability in at-risk populations. Further research is required to determine if more than 1 anti-HIV antibody is needed for optimal efficacy and to avoid HIV escape mutations. Thus, additional mAbs against HIV-1 and other STI pathogens, as well as contraceptive mAbs [53], could be included in an eventual commercial mAb-MPT product to provide more comprehensive protection.

## Supporting information

S1 CONSORT Checklist CONSORT 2010 checklist of information to include when reporting a randomized trial for MB66-01. (PDF)

S1 Table MB66-01 inclusion criteria. (DOCX)

S2 Table MB66-01 exclusion criteria. (DOCX)

S3 Table Details of study samples and laboratory testing. (DOCX)

S4 Table Segment A adverse events (AE). (DOCX)

S5 Table Segment B adverse events (AE). (DOCX)

S6 Table Summary of adverse events (AE) by study arm. (DOCX)

S1 Fig MB66 film dissolution (represented by means ± SEMs) in Segments A (following single film insertion) and B (following single and repeated film insertions). Percent dissolution did not differ significantly in Segment A over time (*p* > 0.10 for all comparisons). In Segment B, % film dissolution at the 1-hour time point was significantly lower than at all other time points (*p* < 0.001 for all comparisons).
(TIF)

S2 Fig Vaginal pH (represented by medians with interquartile ranges) in Segments A (following single film insertion) and B (following single and repeated film insertions). Differences were not statistically significant in either Segment A (*p* = 0.36) or Segment B [Group main effect (i.e., Active film vs. Placebo film, *p* = 0.33) and Visit main effect (*p* = 0.74)].
(TIF)

S3 Fig Nugent scores (measure of vaginal microbial environment; range: 0–10; represented by medians with interquartile ranges) in Segments A (following single film insertion) and B (following single and repeated film insertions). Differences were not statistically significant in either Segment A (*p* = 0.47) or Segment B [Group main effect (i.e., Active film vs. Placebo film, *p* = 0.59) and Visit main effect (*p* = 0.68)].
(TIF)

S4 Fig Neutralization of 3 HIV-1 strains (as assessed by TZM-bl HIV neutralization assay) and HSV-2 (as assessed by HSV-2 Plaque Reduction Neutralization assay) by CVLs before and after MB66 film insertion in Segment A. Assays were performed in triplicate, and data are represented as medians with interquartile ranges. Asterisks (*<0.05, **0.02, and ***<0.01) indicate statistically significant differences compared to Baseline by Tukey Multiple Comparison Test following a significant repeated measures ANOVA.
(TIF)

S5 Fig Neutralization of 3 HIV-1 strains (as assessed by TZM-bl HIV neutralization assay) and HSV-2 (as assessed by HSV-2 Plaque Reduction Neutralization assay) by CVLs before and at various time points following MB66 Active and Placebo film insertion in Segment B. Assays were performed in triplicate, and data are represented as medians with interquartile ranges. Asterisks (*) indicate statistically significant differences compared to Baseline and deltas (Δ) indicate statistically significant difference compared to respective Placebo time points. See text for exact *p*-values.
(TIF)

S6 Fig Boxplots of proinflammatory cytokine concentrations (pg/mL) in CVLs before and at various time points following MB66 Active and Placebo film insertion in Segment B. The Group main effect (i.e., Active vs. Placebo film) was not significant for any CVL cytokine variable. There were a number of significant visit effects (for both Active and Placebo film groups). For TNF-α, the 24-hour post seventh film time point was significantly higher than Baseline (*p* = 0.01) or the 7-day post seventh film time point (*p* = 0.02). For IL-6, Baseline was significantly higher than the 24-hour post seventh film time point (*p* = 0.03). For IL-1α, Baseline and the 24-hour post first film time point were significantly higher than the 24-hour post seventh film and the 7-day post seventh film time points (*p* < 0.001 for all comparisons). Similar results were found for IL-1β (*p* < 0.05 for all comparisons). For both IL-1RA and IL-1RA/IL-1, the 7-day post seventh film time point was significantly higher than the other 3 time points (*p* < 0.05 for all comparisons).
(TIF)

S7 Fig Boxplots of chemokine concentrations (pg/mL) in CVLs before and at various time points following MB66 Active and Placebo film insertion in Segment B. As for proinflammatory cytokines above, the Group main effect (i.e., Active vs. Placebo film) was not significant for any CVL chemokine variable, and there were a number of significant visit effects. For RANTES, the 24-hour post seventh film time point was significantly higher than the other 3 time points (p < 0.05 for all comparisons). For IL-8, Baseline and the 24-hours post first film time point were significantly higher than the 24-hour post seventh film and the 7-day post seventh film time points (*p* < 0.05 for all comparisons). For IP-10, Baseline was significantly higher than the 24-hour post seventh film time point (*p* = 0.02). For MCP-1, the Baseline was significantly higher than the 24-hour post seventh film time point for the Active film group only (*p* = 0.02).
(TIF)

S1 Study Protocol Study protocol for “A Phase 1, Single Center Study to Assess the Safety of MB66, a Combined Anti-HIV (VRC01-N) and Anti-HSV (HSV8-N) Monoclonal Antibody Film for Vaginal Application as Microbicide (MB66-01)”. (PDF)

## Abbreviations

AE: adverse event
CHO: Chinese Hamster ovary
CVL: cervicovaginal lavage
HEC: hydroxyethyl cellulose
HIV-1: human immunodeficiency virus-type 1
HSV-2: herpes simplex virus-type 2
IVR: intravaginal ring
mAbs: monoclonal antibodies
MPT: multipurpose prevention technology
N-9: nonoxynol-9
PK: pharmacokinetics
STI: sexually transmitted infections
