# Supplementary material for: Safety, acceptability, and pharmacokinetics of a monoclonal antibody-based vaginal multipurpose prevention film (MB66): A Phase I randomized trial
Source: PLoS Med. 2021 Feb 3;18(2):e1003495. doi: 10.1371/journal.pmed.1003495 (PMC7857576; doi:10.1371/journal.pmed.1003495)
Supplement: S1 Table — (DOCX) [file pmed.1003495.s002.docx]

**S1 Table. MB66-01 Inclusion Criteria**

## **Inclusion Criteria**

1. Age 18 through 45 years (inclusive) at screening.
2. Able and willing to provide written informed consent to be screened for and enrolled in the study.
3. Able and willing to provide adequate locator information at screening.
4. HIV-uninfected based on testing performed by study staff at screening.
5. In general good health as determined by the site clinician.
6. Agree to abstain from any vaginal insertions, including products, douches, devices such as sex toys, or penile or oral intercourse from 5 days prior to Visit 2 (Enrollment Visit) until the final Study Visit (one week after last dose of study film). Only tampons during menses and clinically indicated speculum exams are allowed.
7. Agree to use condoms provided by the study staff from one week after last use of study film until three weeks after last use of study film.
8. Willingness to undergo all study-related assessments and follow all study-related procedures.
9. Be currently using an effective method of contraception at enrollment (used continuously and with good compliance for the past 60 days as determined by participant self-report) with plans to continue use throughout the study period. Acceptable methods include any hormonal method (except vaginal ring); intrauterine device (IUD) inserted at least 90 days prior to enrollment; female sterilization; abstinent from sexual activity with male partner for the past 60 days; sexual activity with vasectomized partner; engages in sex exclusively with women.
10. For participants 21 and older, a Pap result in the 36 calendar months prior to the Enrollment Visit consistent with Grade 0 according to the Female Genital Grading Table for Use in Microbicide Studies Addendum 1 to the DAIDS Table for Grading Adult and Pediatric Adverse Events^1^, satisfactory evaluation with no treatment required of non-Grade 0 Pap result per American Society for Colposcopy and Cervical Pathology (ASCCP) guidelines or per local standard of care, within the last 36 calendar months prior to enrollment.

*Note: For participants aged 18-21, a Grade-0 or adequately evaluated abnormal Pap smear is not required as the American Society for Colposcopy and Cervical Pathology recommends initiating screening at age 21.*

1. At screening and enrollment, agrees not to participate in other research studies involving drugs, medical devices, or vaginal products while enrolled in this trial.

^1^DAIDS. Female Genital Grading Table for Use in Microbicide Studies (Addendum 1 to the DAIDS Table for Grading Adult and Pediatric Adverse Events, Version 1.0, November, 2007 [Clarification dated August 2009]. 2009.
