# Supplementary material for: Safety, acceptability, and pharmacokinetics of a monoclonal antibody-based vaginal multipurpose prevention film (MB66): A Phase I randomized trial
Source: PLoS Med. 2021 Feb 3;18(2):e1003495. doi: 10.1371/journal.pmed.1003495 (PMC7857576; doi:10.1371/journal.pmed.1003495)
Supplement: S2 Table — (DOCX) [file pmed.1003495.s003.docx]

## **S2 Table. MB66-01 Exclusion Criteria**

## **Exclusion Criteria**

1. Menopausal at screening (as defined as amenorrhea or irregular periods for one year or more without an alternative etiology).
2. Hysterectomy
3. Known adverse reaction to any of the study products (ever).
4. Known adverse reaction to latex (ever).
5. Non-therapeutic injection drug use in the 12 months prior to screening.
6. Surgical procedure involving the pelvis in the 90 days prior to screening (includes dilation and curettage or evacuation, and cryosurgery; does not include cervical biopsy for evaluation of an abnormal pap smear or IUD placement).
7. Participation in a drug, spermicide and/or microbicide study in the 30 days prior to screening or anticipated participation in an investigational drug study in the ensuing 8 weeks.
8. Pregnancy within 90 days prior to screening.
9. Lactating
10. Use of a diaphragm, NuvaRing, or spermicide for contraception.
11. As determined by the PI, a degree of menstrual cycle irregularity that would make it difficult to schedule follow-up visits without interruption by menses.
12. Active sexually transmitted infection or documented treatment of sexually transmitted infections in the last 6 months, including, but not limited to: chlamydia, gonorrhea, syphilis, trichomonas, cervicitis or pelvic inflammatory disease, or currently active HSV lesions or other sores. (Participants seropositive for or with a history of HSV without current active lesions will not be excluded.)
13. Women who by history engage in condom-less intercourse with HIV-infected partners, or who exchange sex for money, shelter, or gifts, or who in the opinion of the investigators, may be at risk for HIV acquisition during the duration of the study.
14. More than one sex partner within the past 3 months.
15. Current sexual partner known by participant to be HIV seropositive.
16. Current or planned use of pre-exposure prophylaxis against HIV infection.
17. Currently active genital HSV lesions, or other genital tract epithelial disruption or inflammation^1^.
18. Current or episodic use of anti-herpes suppressive therapy.
19. Urinary tract infection, symptomatic candidiasis, or symptomatic bacterial vaginosis within 14 days of enrollment, or currently residual symptoms thereof. Enrollment allowed after treatment and resolution of the infection.
20. Antibiotic or antifungal therapy (vaginal or systemic) within 7 days of enrollment.
21. Use of systemic immunomodulatory medications within 4 weeks of enrollment.
22. Menses or other vaginal bleeding at the time of enrollment or expecting menses in the 10 days after enrollment (Segment A participants) or 16 days after enrollment (for Segment B participants).
23. Lack of stable living conditions to allow reliable room temperature storage of study product (Segment B participants only).
24. At enrollment has any of the following laboratory abnormalities per the Division of AIDS (DAIDS) Table for Grading the Severity of Adult and Pediatric Adverse Events, Version 2.0, Nov 2014^2^: Grade 1 or higher AST, ALT, creatinine or platelets; Grade 2 or higher hemoglobin. Note: Otherwise eligible participants with an exclusionary test may be re-tested once during the screening process.
25. As determined by the Principal Investigator (PI), any subject who has any significant uncontrolled active or chronic cardiovascular, renal, liver, hematologic, neurologic, gastrointestinal, psychiatric, endocrine, respiratory, immunologic disorder or infectious disease, anticoagulation with warfarin or heparin; or any other condition that, in the opinion of the Investigator, would preclude provision of consent, make participation in the study unsafe, complicate interpretation of study outcome data, or otherwise interfere with achieving the study objectives.

^1^Prescreening for HSV-1 and HSV-2 serostatus was not done since since HSV8-N binds to and neutralizes both HSV-1 and HSV-2, and only a minority of potential subjects are negative for antibody to both viruses.

^2^DAIDS. Table for Grading the Severity of Adult and Pediatric Adverse Events, Version 2.0 (November, 2014), 2014.
