# Supplementary material for: Safety, acceptability, and pharmacokinetics of a monoclonal antibody-based vaginal multipurpose prevention film (MB66): A Phase I randomized trial
Source: PLoS Med. 2021 Feb 3;18(2):e1003495. doi: 10.1371/journal.pmed.1003495 (PMC7857576; doi:10.1371/journal.pmed.1003495)
Supplement: S3 Table — (DOCX) [file pmed.1003495.s004.docx]

**S3 Table. Details of Study Samples and Laboratory Testing**

- Urine Samples. Study staff at the clinical site used dipstick urinalysis to screen for possible urinary tract infection. Urine was tested for HCG via the QuPID One-Step Pregnancy Test, and for CT, GC, and trichomonas by Nucleic Acid Amplification Test (NAAT, Gen Probe APTIMA).
- Saliva Samples. OraQuick ADVANCE Rapid HIV-1/2 Antibody Test was utilized to test for HIV antibodies (non-gp120 based, CLIA exempt). When required, follow-up testing on blood was performed by the Miriam Hospital Laboratory.
- Blood Samples. Screening and follow-up safety blood tests (CBC, Liver and Renal Panels, and RPR) were performed on blood samples by the Miriam Hospital Laboratory. Blood samples were also analyzed for mAb levels.
- Vaginal Swabs. Vaginal swabs were used for testing for vaginal pH.
- Vaginal Smears. Bacterial morphotypes were assessed by Nugent Scoring of Gram stained vaginal smears^1^.
- CVL Samples. Cervicovaginal lavage (CVL) samples were collected with speculum in place, using 5 mL of Dulbecco’s phosphate buffered saline dispensed three times through a syringe to wash the vaginal walls and ectocervix, then recollected from the posterior fossa. Samples were centrifuged at 500 x g for 10 min to obtain the supernatant for analysis. CVL sampling was performed at Baseline (Screening), 24 hrs after single film insertion (Visit 3) and 7 days after single film insertion (Visit 4) for Segment A, and at Baseline (Screening), 24 hrs after first film (Visit 3), 24 hrs after seventh film (Visit 4) and 7 days after seventh film (Visit 5) for Segment B. These samples were used for mAb levels, viral neutralization studies (against HIV-1 and HSV-2) and immune mediator testing.
- TearFlo Sampling. Vaginal PK measurements were achieved by TearFlo (Beaver-Visitec International, Waltham, MA, USA) filter paper sampling method^2-5^ in order to sample multiple discrete locations for the purpose of observing the extent of distribution of the mAbs in the vagina as a function of time since dosing. TearFlo samples were taken from the distal vagina, mid-vagina, ectocervix and cervical os at 1 hr, 4 hrs and 24 hrs following one MB66 dose (Segments A and B), 7 days following a single dose (Segment A), and at 24 hrs and 7 days post seventh dose (Segment B). Specifically, the filter paper strips were applied to the epithelial surface along their length until saturated, and cut off at the 15 mm mark during insertion into a sample tube containing 0.5 mL of phosphate buffered saline. Pilot experiments demonstrated that this procedure resulted in uptake of approximately 30 mg of sample.

^1^Nugent RP, Krohn MA, Hillier SL. Reliability of diagnosing bacterial vaginosis is improved by a standardized method of gram stain interpretation. J Clin Microbiol. 1991;29(2):297-301.

^2^ Bennetto-Hood C, Johnson VA, King JR, Hoesley CJ, Acosta EP. Novel methodology for antiretroviral quantitation in the female genital tract. HIV Clin Trials. 2009;10(3):193-9.

^3^John GC, Sheppard H, Mbori-Ngacha D, Nduati R, Maron D, Reiner M, et al. Comparison of techniques for HIV-1 RNA detection and quantitation in cervicovaginal secretions. J Acquir Immune Defic Syndr. 2001;26(2):170-5.

^4^Quesnel A, Cu-Uvin S, Murphy D, Ashley RL, Flanigan T, Neutra MR. Comparative analysis of methods for collection and measurement of immunoglobulins in cervical and vaginal secretions of women. J Immunol Methods. 1997;202(2):153-61.

^5^Sherlock CH, Lott PM, Money DM, Merrick L, Arikan Y, Remple VP, et al. Use of Sno Strip filter-paper wicks for collection of genital-tract samples allows reproducible determination of human immunodeficiency virus type 1 (HIV-1) RNA viral load with a commercial HIV-1 viral load assay. J Clin Microbiol. 2006;44(3):1115-9.
