# Supplementary material for: Safety, acceptability, and pharmacokinetics of a monoclonal antibody-based vaginal multipurpose prevention film (MB66): A Phase I randomized trial
Source: PLoS Med. 2021 Feb 3;18(2):e1003495. doi: 10.1371/journal.pmed.1003495 (PMC7857576; doi:10.1371/journal.pmed.1003495)
Supplement: S4 Table — (DOCX) [file pmed.1003495.s005.docx]

**S4 Table. Segment A Adverse Events (AE)**

| **Part. ID** | **AE Description** | **Maximum Grade Per AE^1^** | **Related to Treatment** |
| --- | --- | --- | --- |
| **101** | Labial Abrasions | 1 | No |
| 101 | Asymptomatic Microscopic Hematuria | 1 | Yes |
| **104** | Uterine Cramping | 1 | No |
| 104 | Vaginal Spotting | 1 | Yes |
| 104 | Proteinuria | 1 | No |
| 104 | Proteinuria | 1 | Yes |
| **108** | Chest Rash | 1 | No |
| 108 | Vaginal Itching | 2 | No |

^1^Grade of Severity: 1=Mild; 2=Moderate; 3=Severe; 4=Life-Threatening
