# Supplementary material for: Safety, acceptability, and pharmacokinetics of a monoclonal antibody-based vaginal multipurpose prevention film (MB66): A Phase I randomized trial
Source: PLoS Med. 2021 Feb 3;18(2):e1003495. doi: 10.1371/journal.pmed.1003495 (PMC7857576; doi:10.1371/journal.pmed.1003495)
Supplement: S5 Table — (DOCX) [file pmed.1003495.s006.docx]

**S5 Table. Segment B Adverse Events (AE)**

| **Part. ID** | **Group** | **AE Description** | **Maximum Grade Per AE^1^** | **Related to Treatment** |
| --- | --- | --- | --- | --- |
| **202** | Active Film | Vaginal Odor | 1 | Yes |
| 202 | Active Film | Cold Symptoms | 1 | No |
| **207** | Active Film | Fainting | 1 | No |
| 207 | Active Film | Proteinuria | 2 | Yes |
| **208** | Active Film | Asymptomatic Microscopic Hematuria | 2 | No |
| **211** | Active Film | Asymptomatic Microscopic Hematuria | 1 | No |
| **213** | Active Film | Uterine Cramping | 1 | Yes |
| 213 | Active Film | Asymptomatic Microscopic Hematuria | 1 | No |
| **215** | Active Film | Asymptomatic Microscopic Hematuria | 1 | No |
| 215 | Active Film | Breast Tenderness | 1 | No |
| **218** | Active Film | Urinary Tract Infection | 2 | No |
| **219** | Active Film | Vaginal Discharge | 1 | Yes |
| 219 | Active Film | Asymptomatic Microscopic Hematuria | 1 | No |
| 219 | Active Film | Asymptomatic Microscopic Hematuria | 1 | Yes |
| **221** | Active Film | Proteinuria | 1 | No |
| **223** | Active Film | Asymptomatic Microscopic Hematuria | 1 | Yes |
| 223 | Active Film | Sinus Congestion | 1 | No |
| 223 | Active Film | Asymptomatic Microscopic Hematuria | 1 | No |
| **226** | Active Film | Vaginal Odor | 1 | No |
| **227** | Active Film | Asymptomatic Microscopic Hematuria | 1 | No |
| 227 | Active Film | Vaginal Spotting | 1 | No |
| 227 | Active Film | Uterine Cramping | 2 | Yes |
| 227 | Active Film | Vaginal Spotting | 1 | No |
| 227 | Active Film | Asymptomatic Microscopic Hematuria | 1 | No |
| **228** | Active Film | Vaginal Spotting | 1 | Yes |
| 228 | Active Film | Vaginal Discharge | 1 | Yes |
| 228 | Active Film | Vaginal Odor | 1 | Yes |
| **203** | Placebo Film | Proteinuria | 3 | No |
| 203 | Placebo Film | Asymptomatic Microscopic Hematuria | 2 | No |
| **204** | Placebo Film | Vaginal Itching | 1 | Yes |
| **209** | Placebo Film | Diarrhea | 1 | No |
| **210** | Placebo Film | Urinary Tract Infection | 2 | Yes |
| **212** | Placebo Film | Vaginal Spotting | 1 | No |
| **214** | Placebo Film | Vaginal Odor | 1 | Yes |
| 214 | Placebo Film | Vaginal Spotting | 1 | Yes |
| 214 | Placebo Film | Vaginal Odor | 1 | No |
| **216** | Placebo Film | Vaginal Spotting | 1 | Yes |
| **217** | Placebo Film | Vaginal Discharge | 1 | Yes |
| 217 | Placebo Film | Asymptomatic Microscopic Hematuria | 1 | Yes |
| **220** | Placebo Film | Vaginal Discharge | 1 | Yes |
| **224** | Placebo Film | Proteinuria | 1 | Yes |
| **225** | Placebo Film | Asymptomatic Microscopic Hematuria | 2 | No |
| **229** | Placebo Film | Vaginal Spotting | 1 | No |
| 229 | Placebo Film | Asymptomatic Microscopic Hematuria | 1 | No |
| 229 | Placebo Film | Proteinuria | 1 | No |

^1^Grade of Severity: 1=Mild; 2=Moderate; 3=Severe; 4=Life-Threatening
