# Supplementary material for: Safety, acceptability, and pharmacokinetics of a monoclonal antibody-based vaginal multipurpose prevention film (MB66): A Phase I randomized trial
Source: PLoS Med. 2021 Feb 3;18(2):e1003495. doi: 10.1371/journal.pmed.1003495 (PMC7857576; doi:10.1371/journal.pmed.1003495)
Supplement: S6 Table — (DOCX) [file pmed.1003495.s007.docx]

**S6 Table. Summary of Adverse Events (AE) by Study Arm**

|  | **Segment A** | | **Segment B** | | | |
| --- | --- | --- | --- | --- | --- | --- |
|  | **Active Film** | | **Active Film** | | **Placebo Film** | |
|  | **(n=9)** | | **(n=15)** | | **(n=14)** | |
|  | **Total^1^** | **Related^2^** | **Total** | **Related** | **Total** | **Related** |
| **No. of AEs** | 8 | 3 | 27 | 10 | 18 | 9 |
|  |  |  |  |  |  |  |
| **AE Category** |  |  |  |  |  |  |
| **Genitourinary** |  |  |  |  |  |  |
| UTI | 0 | 0 | 1 | 0 | 1 | 1 |
| Uterine Cramping | 1 | 0 | 2 | 2 | 0 | 0 |
| Vaginal Spotting | 1 | 1 | 3 | 1 | 4 | 2 |
| Vaginal Discharge | 0 | 0 | 2 | 2 | 2 | 2 |
| Vaginal Itching | 1 | 0 | 0 | 0 | 1 | 1 |
| Vaginal Odor | 0 | 0 | 3 | 2 | 2 | 1 |
| Labial Abrasions | 1 | 0 | 0 | 0 | 0 | 0 |
| Asymptomatic Microscopic Hematuria | 1 | 1 | 10 | 2 | 4 | 1 |
| Proteinuria | 2 | 1 | 2 | 1 | 3 | 1 |
| **Other Clinical** |  |  |  |  |  |  |
| Cold Symptoms | 0 | 0 | 1 | 0 | 0 | 0 |
| Sinus Congestion | 0 | 0 | 1 | 0 | 0 | 0 |
| Diarrhea | 0 | 0 | 0 | 0 | 1 | 0 |
| Fainting | 0 | 0 | 1 | 0 | 0 | 0 |
| Breast Tenderness | 0 | 0 | 1 | 0 | 0 | 0 |
| Chest Rash | 1 | 0 | 0 | 0 | 0 | 0 |

^1^Total number of AEs

^2^Number related to study product
