# Supplementary figures and images for: Safety, acceptability, and pharmacokinetics of a monoclonal antibody-based vaginal multipurpose prevention film (MB66): A Phase I randomized trial
Source: PLoS Med. 2021 Feb 3;18(2):e1003495. doi: 10.1371/journal.pmed.1003495 (PMC7857576; doi:10.1371/journal.pmed.1003495)

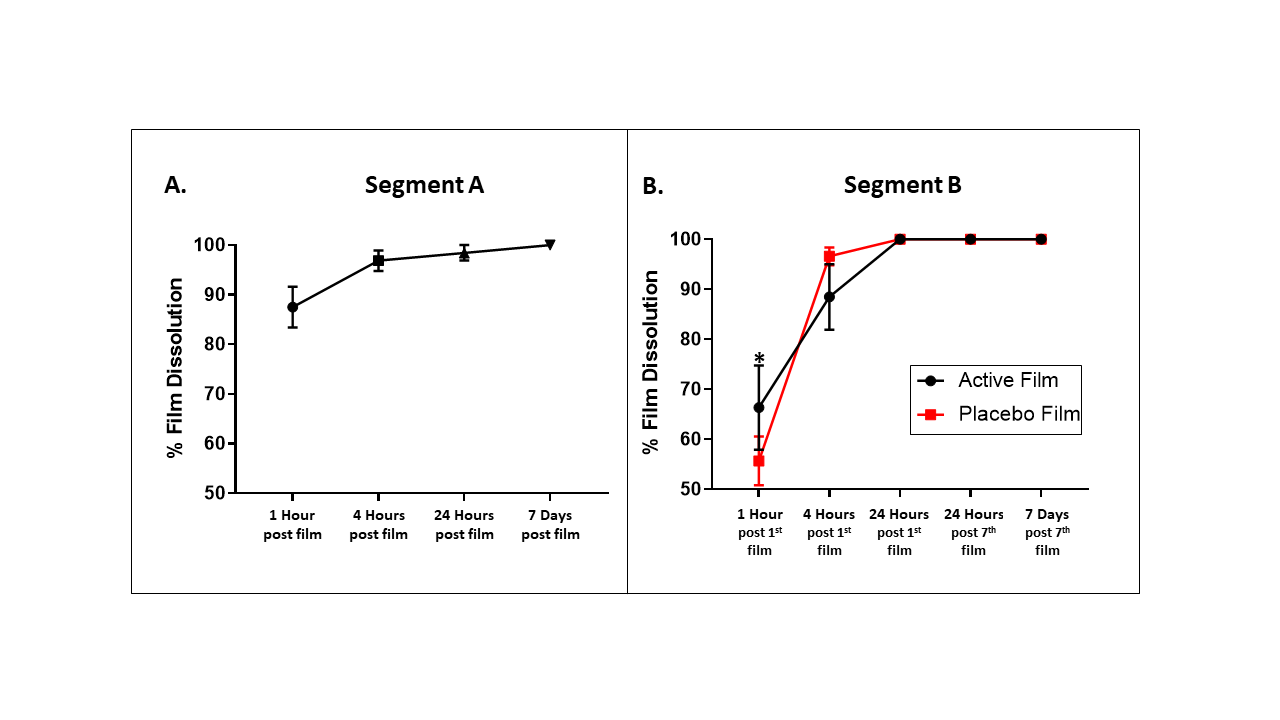

Supplement: S1 Fig — Percent dissolution did not differ significantly in Segment A over time (p > 0.10 for all comparisons). In Segment B, % film dissolution at the 1-hour time point was significantly lower than at all other time points (p < 0.001 for all comparisons). (TIF) [file pmed.1003495.s008.tif]

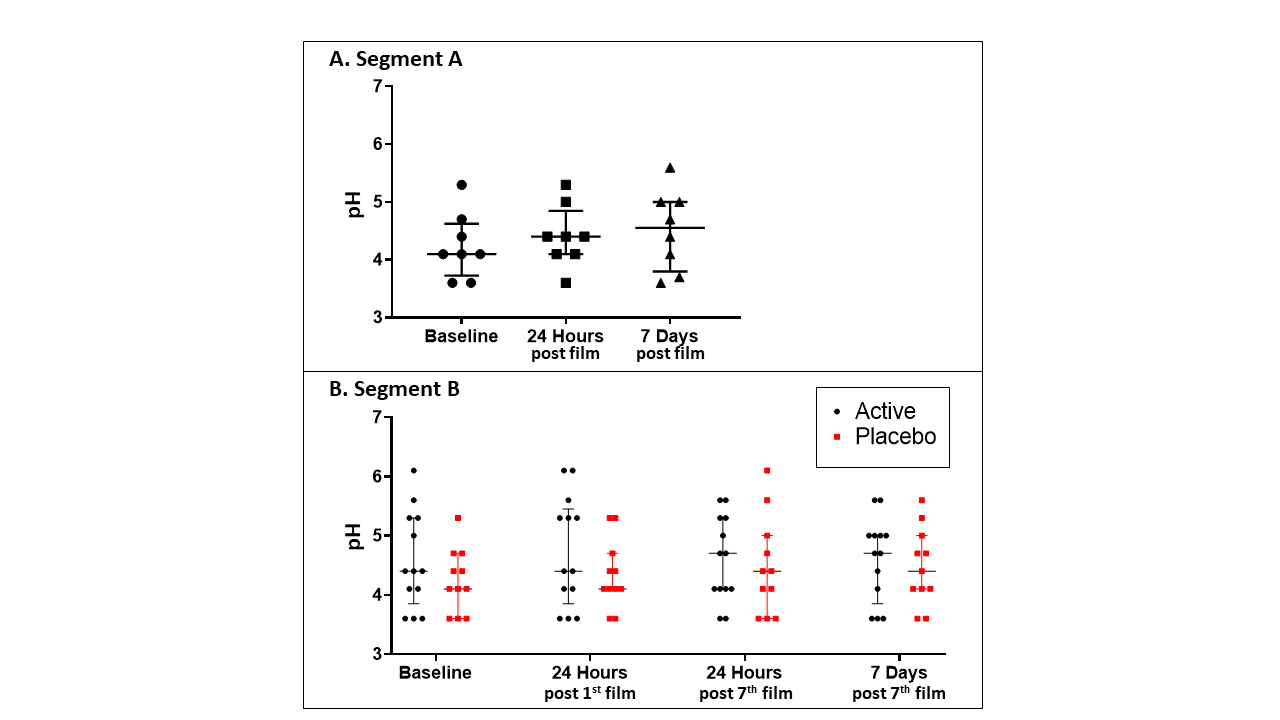

Supplement: S2 Fig — Differences were not statistically significant in either Segment A (p = 0.36) or Segment B [Group main effect (i.e., Active film vs. Placebo film, p = 0.33) and Visit main effect (p = 0.74)]. (TIF) [file pmed.1003495.s009.tif]

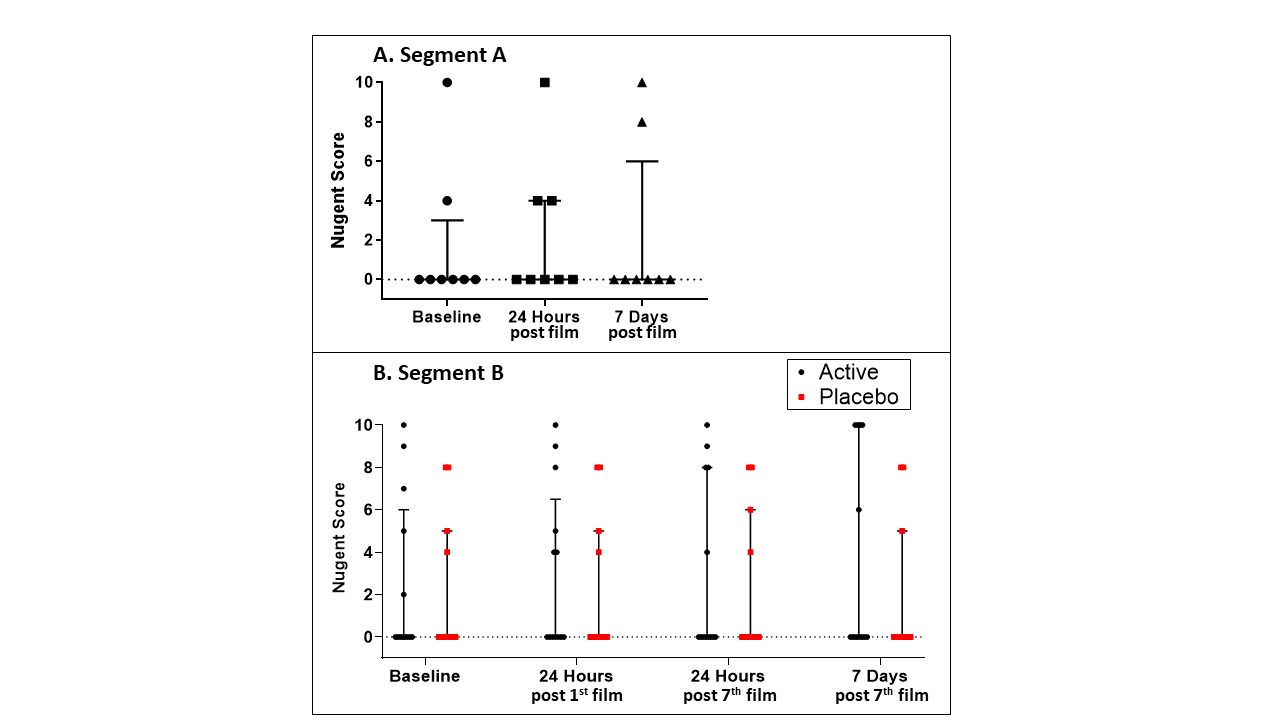

Supplement: S3 Fig — Differences were not statistically significant in either Segment A (p = 0.47) or Segment B [Group main effect (i.e., Active film vs. Placebo film, p = 0.59) and Visit main effect (p = 0.68)]. (TIF) [file pmed.1003495.s010.tif]

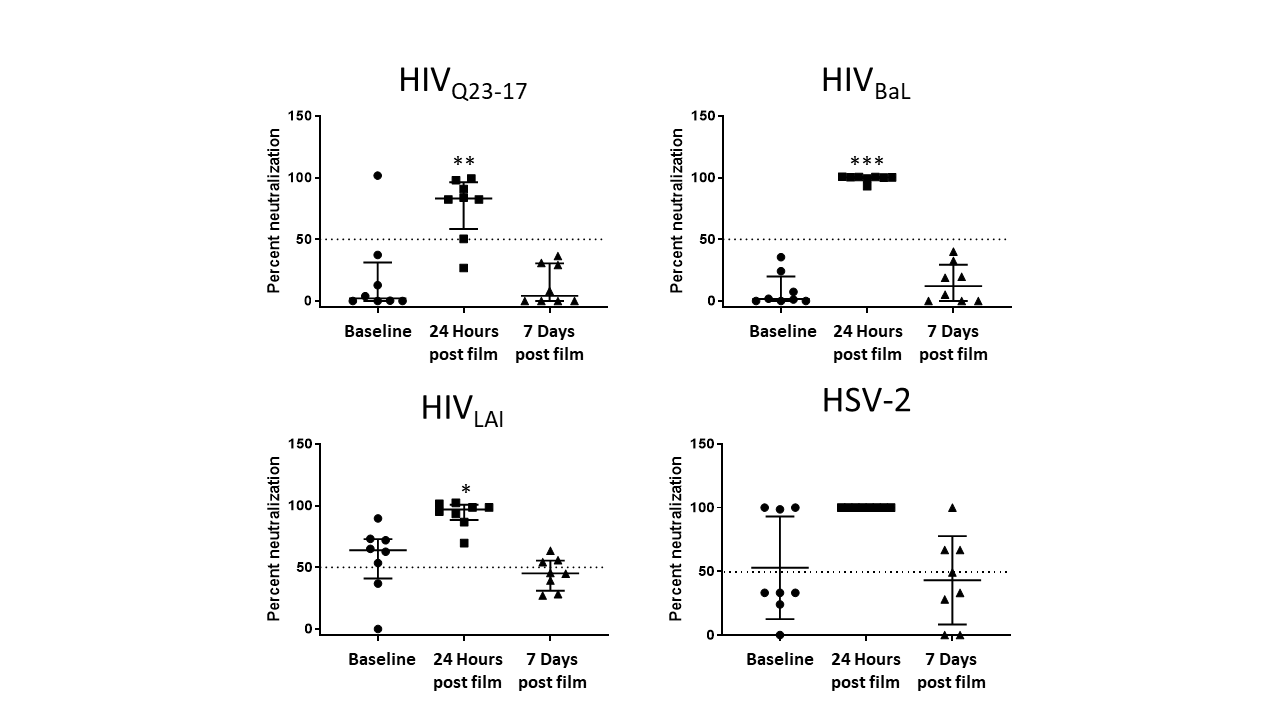

Supplement: S4 Fig — Assays were performed in triplicate, and data are represented as medians with interquartile ranges. Asterisks (*<0.05, **0.02, and ***<0.01) indicate statistically significant differences compared to Baseline by Tukey Multiple Comparison Test following a significant repeated measures ANOVA. (TIF) [file pmed.1003495.s011.tif]

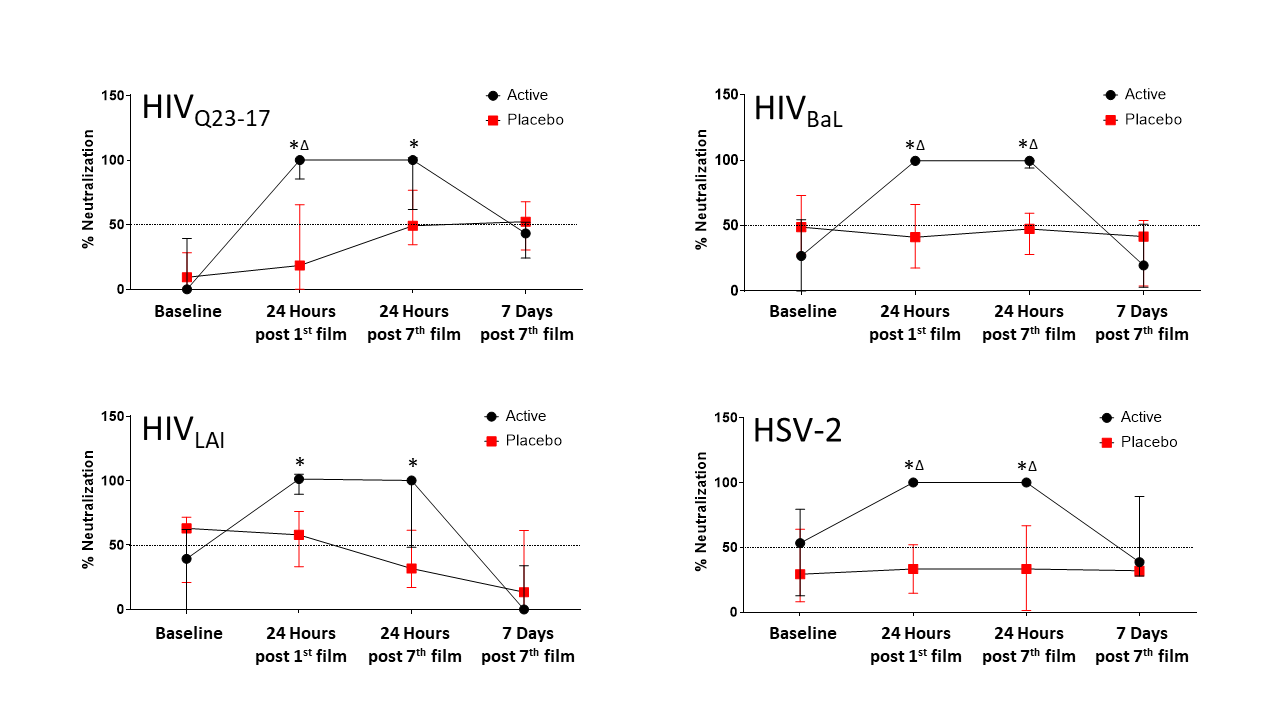

Supplement: S5 Fig — Assays were performed in triplicate, and data are represented as medians with interquartile ranges. Asterisks (*) indicate statistically significant differences compared to Baseline and deltas (Δ) indicate statistically significant difference compared to respective Placebo time points. See text for exact p-values. (TIF) [file pmed.1003495.s012.tif]

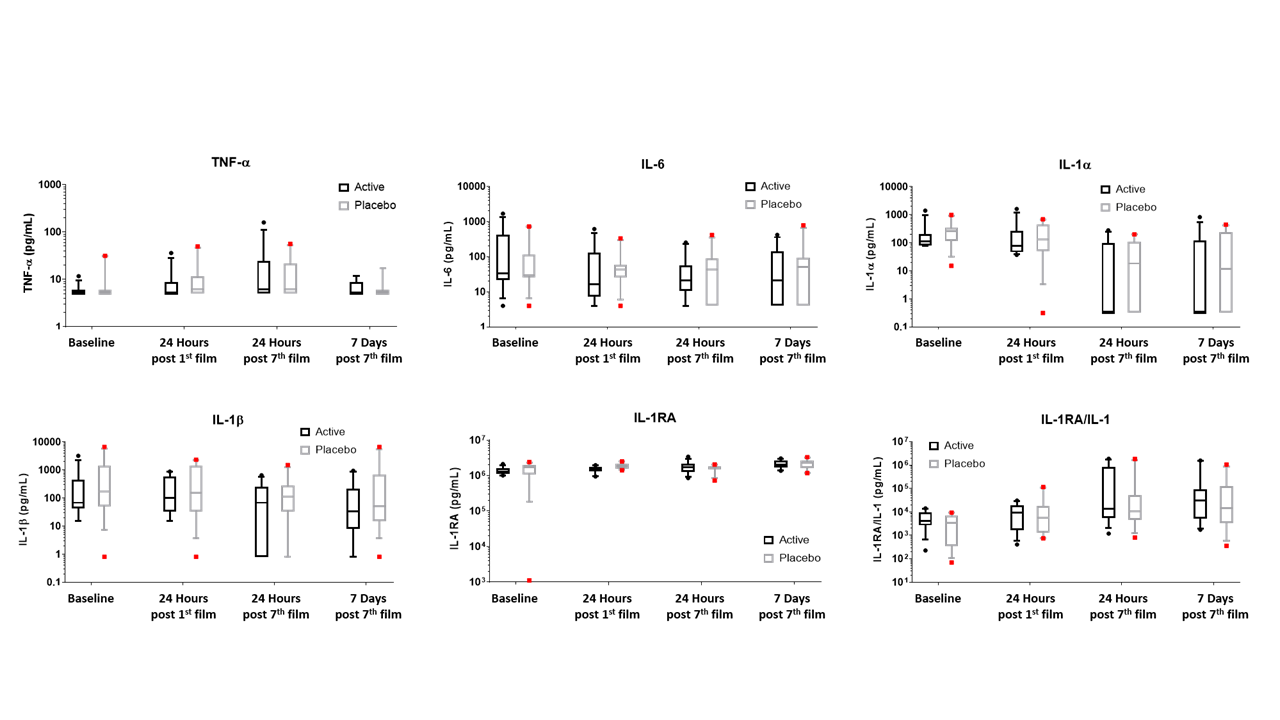

Supplement: S6 Fig — The Group main effect (i.e., Active vs. Placebo film) was not significant for any CVL cytokine variable. There were a number of significant visit effects (for both Active and Placebo film groups). For TNF-α, the 24-hour post seventh film time point was significantly higher than Baseline (p = 0.01) or the 7-day post seventh film time point (p = 0.02). For IL-6, Baseline was significantly higher than the 24-hour post seventh film time point (p = 0.03). For IL-1α, Baseline and the 24-hour post first film time point were significantly higher than the 24-hour post seventh film and the 7-day post seventh film time points (p < 0.001 for all comparisons). Similar results were found for IL-1β (p < 0.05 for all comparisons). For both IL-1RA and IL-1RA/IL-1, the 7-day post seventh film time point was significantly higher than the other 3 time points (p < 0.05 for all comparisons). (TIF) [file pmed.1003495.s013.tif]

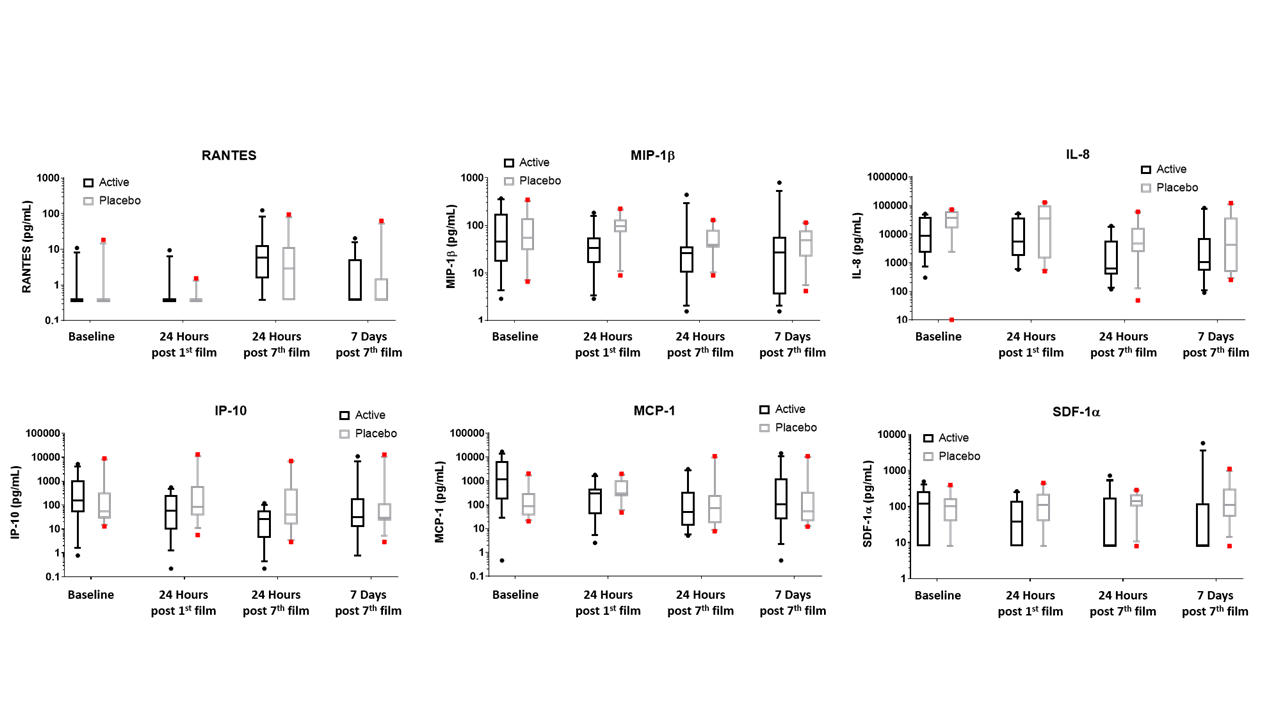

Supplement: S7 Fig — As for proinflammatory cytokines above, the Group main effect (i.e., Active vs. Placebo film) was not significant for any CVL chemokine variable, and there were a number of significant visit effects. For RANTES, the 24-hour post seventh film time point was significantly higher than the other 3 time points (p < 0.05 for all comparisons). For IL-8, Baseline and the 24-hours post first film time point were significantly higher than the 24-hour post seventh film and the 7-day post seventh film time points (p < 0.05 for all comparisons). For IP-10, Baseline was significantly higher than the 24-hour post seventh film time point (p = 0.02). For MCP-1, the Baseline was significantly higher than the 24-hour post seventh film time point for the Active film group only (p = 0.02). (TIF) [file pmed.1003495.s014.tif]
