# Supplementary material for: Safety, acceptability, and pharmacokinetics of a monoclonal antibody-based vaginal multipurpose prevention film (MB66): A Phase I randomized trial
Source: PLoS Med. 2021 Feb 3;18(2):e1003495. doi: 10.1371/journal.pmed.1003495 (PMC7857576; doi:10.1371/journal.pmed.1003495)
Supplement: S1 Study Protocol — (PDF) [file pmed.1003495.s015.pdf]

1. TITLE PAGE

Full Title:

A Phase 1, Single Center Study to Assess the Safety of MB66, a Combined Anti-HIV (VRC01-N) and Anti-HSV (HSV8-N) Monoclonal Antibody Film for Vaginal Application as Microbicide (MB66-01)

DAIDS-ES Document Number 11941

Funding Agency:
Division of AIDS, US National Institute of Allergy and Infectious Diseases
US National Institutes of Health

Grant Number: U19AI096398

IND Sponsor:
LeafBio, Inc., 6160 Lusk Blvd., Suite C105, San Diego, CA 92121

IND # 122,010

Protocol Chair/Co-Chair:

Susan Cu-Uvin, M.D.
Kenneth Mayer M.D

Final Version: 2.0
Version Date: September 1, 2015

2. TABLE OF CONTENTS

1. TITLE PAGE 1
2. TABLE OF CONTENTS 2
3. KEY ROLES 7
4. LIST OF ABBREVIATIONS AND DEFINITIONS 11
5. PROTOCOL SUMMARY 13
6. INTRODUCTION 17
   6.1 BACKGROUND INFORMATION 17
      6.1.1 HIV-1 and HSV-2 Prevention and Microbicides 17
      6.1.2 Integrated Preclinical/Clinical Program 18
      6.1.3 The MB66 Study Product 19
   6.2 RATIONALE 30
      6.2.1 Product Rationale 30
      6.2.2 Trial design rationale 32
   6.3 STUDY HYPOTHESES 34
7. STUDY OBJECTIVES 35
   7.1 PRIMARY OBJECTIVE 35
   7.2 SECONDARY OBJECTIVES 35
   7.3 EXPLORATORY OBJECTIVES 35
8. STUDY DESIGN 35
   8.1 OVERVIEW 35
      8.1.1 Study Endpoints 36
   8.2 DESCRIPTION OF STUDY POPULATION 37
   8.3 TIME TO COMPLETE ACCRUAL 37
   8.4 STUDY GROUPS 37
      8.4.1 Segment A: 37
      8.4.2 Segment B: 37
   8.5 EXPECTED DURATION OF PARTICIPATION 38
   8.6 STUDY SITE 38
9. STUDY POPULATION 38
   9.1 SELECTION OF STUDY POPULATION 38
   9.2 INCLUSION CRITERIA 38
   9.3 EXCLUSION CRITERIA 39
   9.4 CO-ENROLLMENT CRITERIA 41
   9.5 RECRUITMENT PROCESS 41
   9.6 PARTICIPANT RETENTION 41
   9.7 PLANNED SAMPLE SIZE 42
10. STUDY PRODUCT 42
   10.1 IDENTITY 42
   10.2 REGIMEN 43

10.3 ADMINISTRATION 43
10.4 STUDY PRODUCT FORMULATION 44
10.5 STUDY PRODUCT STABILITY 46
10.6 STUDY PRODUCT SUPPLY AND ACCOUNTABILITY 46
10.7 STUDY PRODUCT DISPENSING 46
10.8 ASSESSMENT OF PARTICIPANT STUDY PRODUCT ADHERENCE 46
10.9 CONCOMITANT MEDICATIONS 47
11. STUDY PROCEDURES/EVALUATIONS 47
11.1 SCHEDULE OF PROCEDURES/EVALUATIONS 47
11.1.1 Segment A (Single administration of study film) 47
11.1.1.1 Segment A, Pre-Screening 47
11.1.1.2 Segment A Visit 1: Screening Visit 47
11.1.1.3 Segment A, Visit 2: Enrollment 49
11.1.1.4 Segment A, Visit 3: Follow-Up Visit Day 1, PK sampling 50
11.1.1.5 Safety Contact 2-3 Days Post Enrollment 51
11.1.1.6 Segment A, Visit 4: Exit Visit, 1 Week Post Enrollment 51
11.1.2 Segment B (Seven daily doses, Active Vs. Placebo film) 52
11.1.2.1 Segment B, Pre-Screening 53
11.1.2.2 Segment B, Visit 1: Screening Visit 53
11.1.2.3 Segment B, Visit 2: Enrollment 54
11.1.2.4 Segment B, Visit 3: Follow-up Day 1 with PK Sampling 55
11.1.2.5 Safety Contact 2-3 Days Post Enrollment 57
11.1.2.6 Segment B, Visit 4: Follow-up Visit Day 7) 57
11.1.2.7 Segment B, Visit 5: Exit Visit, Day 14 58
11.2 CONTINGENT EVENTS AND PROCEDURES 59
11.2.1 Participants Who Become Infected with HIV 59
11.2.2 Participants Who Become Pregnant 60
11.2.3 Interim (Ad Hoc) Visits 60
11.2.4 Study Discontinuation Procedures 60
11.3 CLINICAL EVALUATIONS AND PROCEDURES 60
11.4 ACCEPTABILITY ASSESSMENT (CASI) 61
11.5 LABORATORY EVALUATIONS 62
11.5.1 Listing of laboratory tests 62
11.5.2 Details of laboratory testing 63
11.5.3 Specimen Preparation, Handling and Shipping 65
11.5.4 Biohazard Containment 65
11.5.5 Total Blood Volume 65
11.6 QUALITY CONTROL AND QUALITY ASSURANCE PROCEDURES 65
12. ASSESSMENT OF SAFETY 65
12.1 ADVERSE EVENT PROCEDURES AND REPORTING REQUIREMENTS 65
12.1.1 Adverse Events Definitions and Documentation 65
12.1.2 Expedited Adverse Event Reporting 67
13. CLINICAL MANAGEMENT 68
13.1 CLINICAL MANAGEMENT OF ADVERSE EVENTS 68
13.2 PREGNANCY 68
13.3 ACQUISITION OF HIV INFECTION ON STUDY 68
13.4 UNEXPECTED MENSTRUAL LIKE BLEEDING 68

13.5 CRITERIA FOR DISCONTINUATION 69
13.6 PRODUCT HOLD 69
13.7 CRITERIA FOR DISCONTINUATION 69
13.7.1 Permanent Intervention Discontinuation, ...69
13.7.2 Permanent Study Discontinuation, ...70
13.8 SAFETY MONITORING 70
13.9 CLINICAL DATA SAFETY REVIEW 71
14. STATISTICAL METHODS 71
14.1 REVIEW OF STUDY DESIGN 71
14.2 SAMPLE SIZE AND ACCRUAL 72
14.3 JUSTIFICATION FOR PLACEBO 73
14.4 STUDY ENDPOINTS 74
14.4.1 Primary Endpoint: Grade 2 Adverse events 74
14.4.2 Secondary Endpoints: Pharmacokinetics (PK) 75
14.4.3 Exploratory Endpoints 75
14.5 BLINDING 76
14.6 RANDOM ASSIGNMENT 76
14.7 DATA MONITORING AND ANALYSIS 77
14.7.1 Data Monitoring 77
14.7.2 Primary Endpoint Analysis 77
14.7.3 Secondary Endpoint Analysis 78
14.7.4 Exploratory Endpoint Analysis 79
14.7.5 Analysis Cohort 80
15. DATA HANDLING AND RECORD KEEPING 80
15.1 DATA QUALITY ASSURANCE 80
15.2 DIRECT ACCESS TO SOURCE DATA AND STUDY AUDITS 81
15.3 ARCHIVING STUDY RECORDS 81
16. CLINICAL SITE MONITORING 81
17. HUMAN SUBJECTS PROTECTION 82
17.1 INSTITUTIONAL REVIEW BOARD/ETHICS COMMITTEE 82
17.2 SPECIAL POPULATIONS 82
17.2.1 Men 82
17.2.2 Children 83
17.2.3 Prisoners 83
17.2.4 Pregnant women 83
17.3 GOOD CLINICAL PRACTICE 83
17.4 INFORMED CONSENT 83
17.5 STORED SAMPLES 84
17.6 SPECIMEN STORAGE AND POSSIBLE FUTURE RESEARCH TESTING 84
17.7 RISK/BENEFIT STATEMENT 84
17.7.1 Risks 84
17.7.2 Social Impact Events 85
17.7.3 Benefits 85
17.8 COMPENSATION 86
17.9 PARTICIPANT CONFIDENTIALITY 86
17.10 CRITICAL EVENT REPORTING 87

17.11 COMMUNICABLE DISEASE REPORTING 87
17.12 ACCESS TO HIV-RELATED CARE 87
17.13 STUDY DISCONTINUATION 87
17.14 APPROVAL OF STUDY PROTOCOL 87
17.15 AMENDING THE PROTOCOL 88
17.16 CONFIDENTIALITY 88
18. ADMINISTRATIVE PROCEDURES 88
18.1 PROTOCOL REGISTRATION 88
18.2 REGULATORY OVERSIGHT 89
18.3 STUDY COORDINATION 89
18.4 CLINICALTRIALS.GOV 89
18.5 PROTOCOL COMPLIANCE 89
18.6 TRAINING PROCEDURES 89
19. PUBLICATION POLICY 89
20. REFERENCES 90
21. APPENDICES 94

List of Figures

Figure 1: Segment A Study Schema 16
Figure 2: Segment B Study Schema 16
Figure 3: Distribution of N-Linked Glycans: IgG1 (Mol %) 21
Figure 4: Vaginal levels of VRC01-N after vaginal gel format dosing in cynomolgus monkeys 24
Figure 5: Vaginal antibody levels after a single dose of ½ MB66 film in Rhesus macaques (N=6) 25
Figure 6: Vaginal antibody levels after three daily doses of ½ MB66 film (N = 6) 26
Figure 7: Protection of cynomolgus macaques by VRC01 & 4E10 against SHIV challenge ... 29
Figure 8: Dose response curve for HSV8-N in vivo protection against HSV-2 30
Figure 9: MB66 film 45

List of Tables

Table 1: Extracellular VRC01-N Staining Observed in Extracellular Proteinaceous Material of Renal Glomeruli and Tubules 23
Table 2: Comparison of toxicology results in film studies vs. the proposed human dose 27
Table 3: Potency of VRC01 expressed in Nicotiana vs. mammalian cells against SHIV 28
Table 4: Study Dose Regimen 43
Table 5: MB66 active film composition 45
Table 6: Segment A, Visit 1 Procedures (Screening Visit) 48
Table 7: Segment A, Visit 2 Procedures (Enrollment) 49
Table 8: Segment A, Visit 3 Procedures (Follow-Up Visit Day 1) 50
Table 9: Segment A, Visit 4 Procedures (Exit Visit 1 Week Post Enrollment) 51
Table 10: Segment B, Visit 1 Procedures (Screening) 53
Table 11: Segment B, Visit 2 Procedures (Enrollment) 55
Table 12: Segment B, Visit 3 Procedures (Follow-up at Day 1) 56
Table 13: Segment B, Visit 4 Procedures (Follow-up at Day 7) 57
Table 14: Segment B, Visit 5 (Exit Visit) Procedures 58
Table 15: Laboratory Test Methods 62
Table 16: Exact Binomial probabilities for different scenarios for Segment A (n=8) for Study MB66-01 72
Table 17: Exact Binomial probabilities for different scenarios for Segment B (n=15) for_Study MB66-01 73
Table 18: Detectable differences and statistical power 74
Table 19: Segment A Study Visits and Procedures 95
Table 20: Segment B Study Visits and Procedures 96

List of Appendices

Appendix 1: Schedule of Study Visits and Procedures 95
Appendix 2: HIV Testing Algorithm 97
Appendix 3: Investigator's Agreement 98
Appendix 4: Informed consents for Segments A and B 99

3. KEY ROLES

**Sponsor**	LeafBio, Inc.<br>6160 Lusk Blvd., Suite C105<br>San Diego, CA 92121
**Funding Agency**	DAIDS/NIAID/NIH<br>5601 Fishers Lane<br>Rockville, MD 20852
**Medical Monitor**	Pharmaceutical Product Development (PPD), Inc.<br>929 North Front Street<br>Wilmington, NC 28401-3331 USA
**Safety Labs**	Miriam Hospital Laboratory<br>Miriam Hospital<br>164 Summit Avenue<br>Providence, RI 02906 USA
**Co Chairs**	Susan Cu-Uvin, M.D.<br>Protocol Chair<br>Professor, Obstetrics and Gynecology, and Medicine<br>Brown University<br>The Miriam Hospital<br>164 Summit Avenue<br>Providence, RI 02906<br>Phone: 401-793-4775<br>Fax: 401-793-4779<br>Email: scu-uvin@Lifespan.org<br>Kenneth Mayer, M.D.<br>Protocol Co-Chair<br>Professor of Medicine, Harvard Medical School<br>Medical Research Director<br>The Fenway Institute<br>Fenway Health<br>Boston, Mass 02215<br>Phone: 617-927-6087<br>Fax: 617-267-0900<br>Email: kmayer@fenwayhealth.org
**Pharmacology**	Jeff Thomas<br>Intertek Pharmaceutical Services<br>3985 Sorrento Valley Blvd., Suite C<br>San Diego, CA 92121<br>Phone: 858-210-3418<br>Email: jeff.thomas@intertek.com

**Immune Mediator Assays, and HIV-1 and HSV-2 Neutralization Studies**	Anderson Laboratory<br>Boston University School of Medicine<br>670 Albany St, Suite 516<br>Boston, MA 02118 USA
**Medical Officers**	Hans M.L. Spiegel, M.D., Contractor<br>Medical Officer<br>Preclinical Microbicide & Prevention Research Branch<br>Prevention Sciences Program<br>HJF-DAIDS, NIAID, NIH<br>5601 Fishers Lane<br>Room 8B51A<br>Rockville, MD 20852<br>Tel: 240-292-4633<br>Fax240-627-3465<br>Email: hans.spiegel@nih.gov<br>Jeanna Piper, M.D.<br>Sr. Medical Officer<br>Clinical Microbicide Research Branch<br>Prevention Sciences Program<br>DAIDS, NIAID, NIH<br>5601 Fishers Lane<br>Room 8B68<br>Rockville, MD 20852<br>Phone: 240-292-4798<br>Fax: 240-627-3465<br>Email: piperj@niaid.nih.gov<br>Thomas Moench, M.D.<br>Co-PI, IPCP U19 AI096398/RFA AI-10-006<br>Member, MB66-01 Protocol Safety Review Team (PSRT)<br>ReProtect, Inc., and Mapp Biopharmaceutical, Inc.<br>703 Stags Head Road<br>Baltimore, MD 21286<br>Tel: 410-516-7258<br>Fax: 410-337-3838<br>Email: tmoench@reprotect.com
**Program Officers**	Jim A. Turpin, Ph.D.<br>Chief<br>Preclinical Microbicide & Prevention Research Branch<br>Prevention Sciences Program<br>DAIDS, NIAID, NIH<br>5601 Fishers Lane<br>Room 8B31<br>Rockville, MD 20852<br>Phone: 301-451-2732<br>Fax: 240-627-3465

	Email: jturpin@niaid.nih.gov<br><br>Kristen A. Porter, Ph.D.<br>Health Scientist Administrator<br>Preclinical Microbicide & Prevention Research Branch<br>Prevention Sciences Program<br>DAIDS, NIAID, NIH/DHHS<br>5601 Fishers Lane<br>Room 8B35<br>Rockville, MD 20852<br>Tel: 240-292-4783<br>Fax: 240-627-3465<br>Email: porterka@niaid.nih.gov
**DAIDS Clinical Operations Representative**	Cherlynn Mathias, R.N., B.S.N.<br>Office of Director<br>Prevention Sciences Program<br>DAIDS, NIAID, NIH<br>5601 Fishers Lane<br>Room MSC 9831<br>Bethesda, MD 20892<br>Phone: 301-292-4791<br>Fax: 240-627-3465<br>Email: cmathias@niaid.nih.gov
**Data Management**	Joseph Politch, Ph.D.<br>Associate Professor<br>Boston University School of Medicine<br>Boston University<br>670 Albany St<br>Boston, MA 02118<br>Tel: 617-414-8486<br>Fax: 617-414-848<br>Email: joseph.politch@bmc.org<br>Kevin Hennegan, MA<br>Senior Director of Clinical Affairs<br>CBR International Corp.<br>2905 Wilderness Place Ste. 202<br>Boulder, CO 80301<br>Phone: 720-746-1190<br>Fax: 720-746-1192<br>khennegan@cbrintl.com
**Study Coordinator**	Helen Patterson, LPN<br>The Miriam Hospital<br>Infectious Diseases Clinical Trials<br>164 Summit Ave, Bldg, 1125 No. Main St.<br>Providence, RI 02906<br>Phone: 401-793-4771

Fax: 401-793-4323
hpatterson@lifespan.org

**Biostatistician**	Ralph D'Agostino Jr. Ph.D.<br>Professor, Biostatistical Sciences<br>Director, Biostatistics Core<br>Comprehensive Cancer Center<br>Wake Forest University School of Medicine<br>Medical Center Boulevard<br>Winston-Salem, NC 27157<br>Phone (Cancer Center) (336) 716-9011<br>Phone (PHS): (336) 716-9410<br>Fax: (336) 716-4128

4. LIST OF ABBREVIATIONS AND DEFINITIONS

**CASI**	Computer Assisted Structured Interview
**ADCC**	Antibody Dependent Cell-Mediated Cytotoxicity
**AE**	Adverse Event
**AIDS**	Acquired Immunodeficiency Syndrome
**ALT**	Alanine Transaminase
**API**	Active Pharmaceutical Ingredient
**AST**	Aspartate Aminotransferase
**BUN**	Blood Urea Nitrogen
**BV**	Bacterial Vaginosis
**CBC**	Complete Blood Count
**CBR**	CBR International Corporation, Boulder CO
**CDC**	Complement Dependent Cytotoxicity
**CRF**	Case Report Form
**CT**	Chlamydia trachomatis
**CVF**	Cervicovaginal Fluid
**CVL**	Cervicovaginal Lavage
**DAERS**	DAIDS Adverse Experience Reporting System
**DAIDS**	Division of AIDS
**EAE**	Expedited Adverse Event
**EC50**	50% Effective Concentration
**FDA**	(United States) Food and Drug Administration
**GC**	Neisseria gonorrhoeae
**GCP**	Good Clinical Practice
**HCG**	Human Chorionic Gonadotropin
**HEC**	Hydroxyethylcellulose
**HIV-1**	Human Immunodeficiency Virus, Type 1

**HSV-1, HSV-2**	Herpes Simplex Virus Type 1, Type 2
**IND**	Investigational New Drug application
**IRB**	Institutional Review Board
**ITT**	Intent-To-Treat
**IUD**	Intrauterine Device
**MAb**	Monoclonal Antibody
**MEMS**	Medication Event Monitoring System
**N-9**	Nonoxynol-9
**NAAT**	Nucleic Acid Amplification Test
**NIAID**	National Institute of Allergy and Infectious Diseases
**NIH**	National Institutes of Health
**OHRP**	Office of Human Research Protections
**PCR**	Polymerase Chain Reaction
**PI**	Principal Investigator
**PP**	Per Protocol
**PSRT**	Protocol Safety Review Team
**PVA**	Polyvinyl Alcohol
**RSC**	Regulatory Support Center
**RT**	Reverse Transcriptase
**SHIV**	Engineered Hybrid Virus with HIV-1 Envelope and SIV Core
**SLPI**	Secretory Leukocyte Protease Inhibitor
**SOP**	Standard Operating Procedure(s)
**STI**	Sexually Transmitted Infection
**WB**	Western Blot
**VCF**	Vaginal Contraceptive Film

5. PROTOCOL SUMMARY

Protocol Summary

**Protocol Title:**	An early Phase 1, Single Center Study to Assess the Safety of MB66, a Combined Anti-HIV (VRC01-N) and Anti-HSV (HSV8-N) Monoclonal Antibody Film for Vaginal Application as Microbicide (MB66-01)
**Short Title**	Safety of MB66 Vaginal Film
**Sample Size**	38 evaluable participants
**Population**	Healthy, HIV-uninfected women, 18 – 45 years of age, at low risk of HIV/STI acquisition, who have normal menstrual cycles
**Participating Site**	Miriam Hospital, Providence, RI
**Study Design**	This is a Pre-Phase 1, single center study with two sequential Segments:<br>**Segment A:** Single arm, single dose, study with 8 evaluable women and a pause for safety assessment upon completion and before initiation of Segment B.<br>**Segment B:** Two arm, repeated dose, randomized, single blind, placebo-controlled trial with 15 evaluable women per arm<br><br>The study will enroll approximately 43 women to reach a total of 38 evaluable women by the end of the study. Evaluable Segment A participants will be defined as those who complete the Screening and Enrollment Visits and return for the Day 1 visit. Evaluable Segment B participants will be defined as those who complete the Screening and Enrollment Visits and return for Visit 4 and are found by MEMS recordings to have used study product for at least 5 of the previous 7 days including the day before presentation. Non-evaluable participants will be replaced.<br><br>Accrual of 38 evaluable participants (8 in Segment A, 30 in Segment B) is expected to take 12 months. The expected duration of study participation for each participant will be approximately 5 to 9 weeks, including the screening period. After enrollment, the study duration will be 1-3 weeks.
**Schedule of Procedures/Evaluations**	A Day 0 dose will be placed in the study clinic in both Segments, and six additional doses will be inserted at home on Days 1-6 in Segment B. PK and safety evaluations will be done on Day 0, Day 1, and Day 7 in both Segments, and additionally on Day 14 for Segment B.

**Study Duration**	Estimated total study duration 12 months, individual participation 5-9 weeks
**Study Regimen/Intervention**	**MB66 film:** Active film contains 10 mg of VRC01-N and 10 mg of HSV8-N monoclonal antibodies (MAbs). In Segment A the single exposure dose will be 1 film (10 mg of each antibody). In Segment B seven daily doses of one film (10 mg of each antibody) will be given to active film recipients, and supplied in MEMS capped (adherence monitoring) vials.<br><br>**Placebo film:** A vehicle control film with same excipients as the active film will be given to the placebo group in Segment B (one film daily for seven consecutive days).
**Primary Objective**	To evaluate the safety of the MB66 film
**Secondary Objectives**	To measure pharmacokinetic parameters including:<br>The rate of MB66 film dissolution<br>The vaginal concentrations of the MB66 antibodies<br>The degree of systemic absorption of the MB66 antibodies
**Exploratory Objectives**	To assess the antiviral effect of the MB66 antibodies *ex vivo* in cervicovaginal lavage fluid from participants after dosing with MB66 film<br>To compare the effects of MB66 and placebo films on the cervicovaginal microbial environment using pH, Nugent score, and bacterial ribosomal DNA PCR<br>To compare the effect of MB66 and placebo films on cervicovaginal immune mediators by Luminex and ELISA assays<br>To assess the acceptability of the MB66 vaginal film after 7 days of use
**Primary Endpoint**	Incidence of Grade 2 or higher Adverse Events deemed related to study product
**Secondary Endpoints**	Naked eye visual assessment during speculum exam of the degree of film dissolution at 1, 4, and 24 hours after dosing<br>Concentrations of MB66 antibodies in vaginal fluid sampled with Tear Flo (filter paper) wicks, and measured by ELISA<br>Concentrations of MB66 antibodies in serum by ELISA

**Exploratory Endpoints**	Assays of the ex vivo viral neutralization of HIV-1 and HSV-2 in cervicovaginal lavage fluid after MB66 dosing<br>Vaginal pH<br>Nugent score on Gram stained vaginal smears<br>Vaginal microbiome assessment by polymerase chain reaction (PCR) for bacterial ribosomal DNA (samples stored for later analysis)<br>Levels of immune mediators in cervicovaginal lavage fluid measured by Luminex and ELISA immunoassays

Figure 1: Segment A Study Schema

	**Visit 1 Screening**	**Visit 2 Enrollment And Dosing**	**Visit 3 Clinical Evaluation**	**Telephone Safety Contact**	**Visit 4 Clinical Evaluation**
	Up to Day-45	*Day 0*	*Day 1*	*Day 3-4*	*Day 6-10*
**Arms and dosing:**					
8 women each receive 1 film		■			
Sampling:					
		PK at 0, 1, 4 hr	PK at 24 hr		PK at 1wk
	Immune modulators		Immune modulators		Immune modulators
		Microbial environment	Microbial environment		Microbial environment

Day -5 *Visit 4* *Day 21*
||----------------------Abstinence--------------------------||--Condom use--||

Figure 2: Segment B Study Schema

**Visit 1 Screening**	**Visit 2 Enrollment And Dosing**	**Visit 3 Clinical Evaluation**	**Telephone Safety Contact**	**Visit 4 Clinical Evaluation**	**Visit 5 Clinical Evaluation**
Up to Day-45	*Day 0*	*Day 1*	*Day 3-4*	*Day 7-8*	*Day 12-16*
**Arms and dosing:**					
15 women: 1 active film/d for 7 d	■	■	■ ■ ■ ■ ■		
15 women: 1 placebo film/d for 7 d	□	□	□ □ □ □ □		
**Sampling:**					
	PK at 0, 1, 4 hr	PK at 24 hr		PK at 1wk	PK at 2wk
Immune Modulators		Immune Modulators		Immune Modulators	Immune Modulators
	Microbial Environment	Microbial environment		Microbial environment	Microbial environment

Day -5 *Visit 4* *Day 28*
||----------------------Abstinence---||--Condom use---||

6. INTRODUCTION

6.1 BACKGROUND INFORMATION

6.1.1 HIV-1 and HSV-2 Prevention and Microbicides

Sexual transmission accounts for the majority of Human Immunodeficiency Virus (HIV-1) infections worldwide (www.unaids.org). Condoms are safe and effective when used consistently and correctly, but are generally perceived as a barrier to intimacy and sexual pleasure, and have relatively low acceptability among both men and women. While condoms are a safe, effective and inexpensive means for preventing heterosexual transmission of HIV-1 when used consistently, women in these populations may not be able to assure condom use by their sexual partners. Accordingly, there is a need for HIV-1 prophylaxes that can be used by women without requiring compliance by their sexual partners.

Similarly, condoms are effective prophylactics for the transmission of herpes simplex virus type 2 (HSV-2). HSV-2 infection leads to genital ulcerations and is associated with an increased risk of HIV-1 acquisition in men and women (Fleming, 1999; Freeman, 2006). Unlike bacterial sexually transmitted infections (STIs), HSV-2 infections cannot be treated with antibiotics; in fact, HSV-2 establishes a state of persistent infection with periodic flare-ups that result in recurrent lesions (Barnabas, 2012). While antiviral treatments that ameliorate these flare ups are available, they are not completely effective at preventing transmission and are relatively expensive for many at-risk populations.

Topical microbicides are agents designed to reduce the acquisition and transmission of sexually transmitted infections, including HIV-1 and HSV-2, when applied to the genital or rectal mucosa. Microbicides have the potential for greater overall acceptability than condoms, as they are less evident during coitus, may be initiated clandestinely by a female partner, may not require negotiation for their use, and generally do not interfere with sexual pleasure or pose a barrier to intimacy.

It was originally anticipated that a microbicide with broad-spectrum non-specific antiviral activity could be developed and rapidly implemented. The over-the-counter vaginal spermicide nonoxynol-9 (N-9) displayed strong antiviral activity *in vitro* due to its detergent properties, but failed to show efficacy in preventing HIV-1 infection in clinical trials. In fact, use of N-9 actually increased the likelihood of HIV-1 transmission, likely because it damaged the vaginal epithelium and promoted inflammation of the mucosa (Friend, 2013). Other microbicide candidates with broad, non-specific mechanisms of action including C31G (Savvy; a detergent), BufferGel (an acidifier), PRO2000 (a polyanion), cellulose sulfate (a polyanion), and Carraguard (a polyanion) also failed to protect against HIV-1 infection in clinical trials (Friend, 2013).

More specifically targeted anti-HIV-1 agents have been the focus of recent vaginal microbicide development. Studies evaluating the efficacy of the antiretroviral drug tenofovir in a hydroxyethyl cellulose (HEC) gel have displayed the most success. A recent study of 1%

tenofovir gel (CAPRISA 004 trial), suggested a significant reduction of HIV-1 transmission when the gel was administered two times in a coitally dependent manner i.e., within 12 hours pre-coitus and again within 12 hours post-coitus (Friend, 2013). High-risk women who used Tenofovir gel before and after intercourse decreased their risk of becoming infected with HIV-1 by at least 39%, and the efficacy increased if subjects were at least 80% adherent. However, a similar trial evaluating the efficacy of 1% Tenofovir gel applied once daily independent of coitus (VOICE trial) showed no effect on vaginal HIV-1 infection (Friend, 2013). Likewise the FACTS 001 study using the CAPRISA 004 dosing regimen failed to demonstrate efficacy, again with low levels of objectively measured adherence.

It has been suggested that vaginal gels have low adherence rates due at least in part to unfavorable side effects including messiness and leakage (Rosen, 2008; Friend, 2013). The high glycerin content and thus the hyperosmotic nature of most gel formulations may cause an influx of liquid into the vagina, exacerbating leakage, and leading to low adherence. The influx of liquid may be circumvented with a film formulation such as the MB66 film, which has a low osmotic load.

Several Phase III trials are currently investigating the efficacy of vaginal microbicides in an intravaginal ring format (dapivarine in the ASPIRE and RING studies), and the intravaginal ring format is expected to improve adherence compared to peri-coital or daily gel formats that have recently shown disappointing results. However, an “on demand” product format rather than a continously worn product may be more acceptable to certain groups of women, most obviously those with infrequent intercourse, or those who object or whose partners object to wearing a vaginal ring. The purpose of the proposed clinical trial is to determine the preliminary safety of MB66, a vaginal film containing one monoclonal antibody against HIV-1 and one monoclonal antibody against HSV-2.

6.1.2 Integrated Preclinical/Clinical Program

The Integrated Preclinical/Clinical Program for HIV Topical Microbicides (IPCP-HTM) supports microbicide development linked with research conducted as a multi-project, multidisciplinary cooperative agreement. The IPCP-HTM seeks to increase the variety of approaches and availability of possible candidate microbicides appropriate for advancement into clinical trials.

The program under the parent grant (U19 AI096398/RFA AI-10-006) brings together a group of research scientists from four academic institutions (Boston University, Johns Hopkins University, University of North Carolina, Emory University), and four companies (Mapp Biopharmaceutical, Aridis Pharmaceuticals, ReProtect, Inc, and Auritec, Inc.). The goal of the program is to develop and evaluate a film formulation of two antiviral monoclonal antibodies, VRC01-N against HIV and HSV8-N against HSV. The program comprises a coordinated combination of basic science studies of the interaction of antibodies with mucus secretions, in vitro and in vivo assessments of antibody potency and efficacy, formulation development, and the early exploratory IND clinical study of the present protocol. The program will generate new

knowledge regarding the feasibility of a multi-antibody film for further safety and efficacy studies for HIV and HSV prevention.

The present protocol describes a Pre-Phase I clinical study within the IPCP program, and in keeping with the pilot character of a Pre-Phase 1 trial and the resources within this program, the study has limitations in its size and goal. Its goal is to take important preliminary steps in exploring the safety and feasibility of several innovative features of this microbicide product. Among these are: monoclonal antiviral antibodies as microbicides, plant based production of these antibodies, a film format for delivery of a biopharmaceutical, and the effect of single vs. multiple daily doses on pharmacokinetics of the MAbs. Despite the limitations of its relatively small size and other limitations, we believe it will contribute important new knowledge on the feasibility of this approach.

6.1.3 The MB66 Study Product

Background: passive topical immunization for prevention

Human monoclonal antibodies (MAbs), typically administered as an intravenous infusion, are an accepted form of treatment for a number of diseases with 34 MAbs approved for licensure in the US and EU as of 2012 (Reichert, 2012). MAbs show promise as topical microbicides because of their proven specificity, flexibility, and broad-spectrum activity. Antibodies are a key component of the human immune system, and anti-pathogen antibodies applied as passive topical immunization are likely to be safe, a critical feature for preventive strategies. Notably, long-term topical administration of MAbs is expected to be safe (Mburu *et al*., 2009). MAbs are now widely used and extraordinarily successful approved products in a host of systemic applications (Reichert, 2012). Clinical trials evaluating the safety of systemic administration of one of the MB66 MAbs (VRC01) are currently ongoing (clinicaltrials.gov), with safety data reviewed in April 2014. Passive immunization is known to provide effective prevention when applied systemically (e.g., against rabies, hepatitis A and B, respiratory syncytial virus and other infections (Casadevall, 2004)). Clinical experience using MAbs for *topical* applications is at this date very limited, but an anti-IL8 MAb product (ABCream, Yes Biotech Laboratories Ltd., Mississauga, Ontario) is marketed in China for the treatment of psoriasis (Huang, 2002). Multiple MAbs can be used in concert as cocktails to provide redundancy against a single pathogen, and/or for broad protection against multiple pathogens. The presently studied MB66 product thus comprises a combination of an anti-HIV and an anti-HSV mMab to provide protection against two incurable viral infections with high and synergistic morbidity.

The MB66 drug product is a vaginal film containing two broadly neutralizing MAbs, an anti-HIV-1 MAb (VRC01-N) and an anti-HSV-2 MAb (HSV8-N). When formulated in a gel, VRC01 protected seven of nine humanized mice (RAG-hu) from HIV infection (Veselinovic *et al*., 2012). MabGel, a different MAb microbicide formulated as a multi-Ab gel (containing three other anti-HIV MAbs, 4E10, 2F5, 2G12), was shown to be partially protective in a macaque SHIV-162P3 vaginal challenge model (Moog *et al*., 2014). In a phase 1 trial of MabGel, the product was shown to be safe (Morris *et al*., 2010).

Unformulated HSV8, a fully human anti-HSV glycoprotein D (gD) Ab which neutralizes a range of low passage clinical isolates of HSV-1 and HSV-2 (de Logu *et al*., 1998), provided 100% protection at 100 µg/ml in a mouse/HSV model (Zeitlin *et al*., 1996; Zeitlin et al., 1997). In another study, complete protection against vaginal challenge with an unformulated anti-HSV gD Ab (produced in soy plants and mammalian cells) required approximately 1 mg/ml for both expression methods (Zeitlin *et al*., 1998). Controlled release of anti-HSV antibodies from ethylene vinyl acetate (EVA)-based vaginal rings demonstrated one week of protection in the HSV/mouse model (Sherwood *et al*., 1996), providing evidence that repeated dosing of antibodies from daily non-coital dosing with formats such as thin films could provide long-term protection.

Mechanism of Action of the MB66 Mabs

Antibodies directed against surface glycoproteins of enveloped viruses may exert inhibitory effects by binding to virus particles and thereby interfering with the ability of the virus to infect cells (virus neutralization). These antibodies can also kill virally infected cells that express the glycoproteins on their surfaces using two mechanisms: antibody dependent cell-mediated cytotoxicity (ADCC) and complement dependent cytotoxicity (CDC). However, as the MAbs in MB66 will be acting against early stages of virus infection, virus neutralization is the mechanism of action for both MAbs. Neutralization of HSV-2 virus and of HIV-1 virus by HSV8 and VRC01, respectively, have been confirmed in vitro (de Logo *et al.*, 1998; Li *et al.*, 2011).

The activity of VRC01 against clinical isolates of HIV-1 has been studied extensively (Li *et al.*, 2011). These results showed that VRC01 binds and neutralizes the large majority of clinical HIV-1 isolates with high affinity. The activity of HSV8 has been tested against three clinical isolates of HSV-2 and three of HSV-1. By virus neutralization, it showed essentially equal efficacy against six of six low passage clinical HSV-1 and HSV-2 isolates (de Logu *et al.*, 1998).

Production of MB66 MAbs in N. Benthamiana

The expression of MAbs in tissue culture systems is expensive, both in ongoing manufacturing costs, and also in the capital costs required to build manufacturing facilities. Production costs can be lowered considerably by expressing human MAbs in plants, as with the *Nicotiana* expression system employed to produce the MAbs contained in the MB66 film. Plant production of therapeutic proteins in general has made considerable progress in commercial pharmaceutical manufacturing; plant-produced proteins for a range of infectious diseases including hepatitis B, human papillomavirus, rabies, malaria, tetanus, and *E. coli* are currently under development (Hefferon, 2013).

The MAbs in MB66 are produced by expression of genetically engineered plasmids in a *Nicotiana benthamiana* based rapid antibody manufacturing platform (RAMP) using proprietary plasmid vectors with plant viral sequences in order to reduce the cost of MB66 production, and thereby make MB66 affordable for women in less developed countries. The use of a transgenic strain of *N. benthamiana* (in which plant specific glycosyltransferases [α1,3 fucosyltransferase and β1,2 xylosyltransferase] are inhibited by siRNA [Strasser, 2008]) allows the RAMP-

produced MAbs to have highly homogenous mammalian N-glycans and mitigates the potential for immunogenicity caused by carryover of plant glycans.

Production in *N. benthamiana* with blocked glycotranserases eliminates the potentially immunogenic plant fucose and xylose linkages, resulting in either aglycosylated or gn/gn glycosylated antibody, the latter present (albeit at low levels) in human immunoglobulins (Figure 3). Moreover, in contrast to the high diversity of glycosylation patterns observed in CHO-expressed antibodies, for example in comparison to the approved systemically-adminstered Mabs Synagis and Rituxan, the glycosylation pattern of the MB66 Mabs is considerably more homogeneous (Figure 3). Finally, the potential for immunogenicity is further reduced with vaginally administered Mabs because of the low potential for systemic absorption, as shown in a recent study of Mabgel, a microbicide gel containing three anti-HIV Mabs (C2F5, C4E10 & C2G12) administered vaginally for 12 consecutive days at up to 50 mg total Mab per dose (Morris, 2012). No detectable systemic toxicity, nor systemic absorption (serum tested by ELISA) was observed in 19 women exposed to the Mab gels (Morris, 2012).

Figure 3: Distribution of N-Linked Glycans: IgG1 (Mol %)

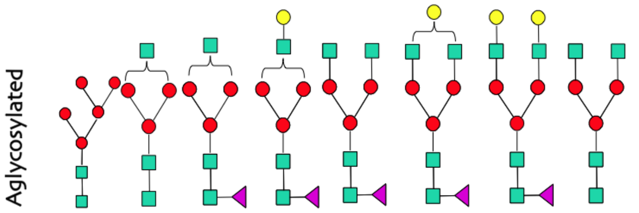

	Aglycosylated	M5	GnM	GnMF	G1M	G0	G1	G2	GnGn
**Synagis**		15		12	7	24	33	9	
**Rituxan**						53	35		8
**HSV8-N**	7								81
**VRC01-N**	6								86

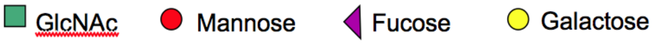

The RAMP technology employs a transient expression system initiated by infiltration of plants with *Agrobacterium* strains carrying plasmids with plant viral genes that encode plant viral polymerases and transport functions. The technology and its applications have been described by Giritch (2006), and has proven versatile with demonstrated expression of numerous heterologous proteins, including cytokines, interferon, bacterial and viral antigens, growth hormone, vaccine antigens, single chain antibodies and MAbs at levels of > 1 g of total soluble protein per kg of fresh biomass.

Specifically, this system uses two magnICON plasmid vectors that carry genes from two different plant virus genomes: turnip vein clearing virus (TVCV) and potato virus X (PVX). The

cDNAs of the virus polymerase genes, encoding the polymerases required for virus RNA replication, are transfected into *N. benthamiana* via an "Agro-infiltration" process that introduces the plasmid vectors, carried in Agrobacterium, to many cells throughout the transfected plant. The plasmid vectors are then "activated" by transcription of the transgenes from a promoter in the transfer or T-DNA region (derived from the Ti plasmid of Agrobacterium) to produce the viral-like RNAs in vivo, and the RNAs are transported to the cytoplasm for RNA amplification via transfected viral polymerases. The magnICON vectors encode the requisite viral proteins for cell to cell movement, including the movement proteins in the TVCV based vector pICH26212 and the triple block products and coat protein in the PVX-based vector pICH31161. These proteins allow movement of the viral-like-RNAs (including the transgenes encoding the VRC01 and HSV8 Mabs) locally within an inoculated leaf, resulting in the majority of cells being infected and becoming production sites for the MAbs. Aerial parts of the plant are harvested at 7 days post infiltration and extracted for the desired MAb. Note that during this process no assembled virus is produced, as the plasmid vectors are 'deconstructed' and are not capable of producing virions.

Downstream processing of the biomass containing the expressed MAbs consists of purification by Protein A chromatography, Capto Q chromatography and Ceramic Hydroxyapatite (CHT) column chromatography followed by ultrafiltration/diafiltration (UF/DF). In-process controls and analysis of purity by SDS-PAGE (under both reducing and non-reducing conditions) and SE-HPLC, as well as monitoring for levels of endotoxin (< 5 EU/mL), bioburden (< 1 CFU/mL, residual host cell DNA (< 6.2 pg/mg) and proteins (< 100 ng/mg), and heavy metals (conforms to USP 232) prior to release of MB66 mAbs ensure that quality attributes are met in accordance with FDA-approved specifications. It is expected that the MAbs comprising MB66 (VRC01-N and HSV8-N) produced by these methods (and designated "-N" to indicate production in *Nicotiana*) carry a low risk of inducing serious toxicities for a number of reasons. Among these are the fully human sequences of the antibodies, a glycosylation pattern also present in human immunoglobulin (see above and Figure 3), and their binding specificity against viruses rather than against self (human) antigen targets. Furthermore, production in plants obviates concerns regarding the transmission of mammalian viruses that might occur in Mabs produced by conventional production in mammalian cell culture.

The MB66 film

MB66 consists of two MAbs, VRC01-N and HSV8-N, in a polyvinyl alcohol (PVA) film. One MB66 film contains 10 mg of each MAb. VRC01-N is directed against the CD4 binding-site of the HIV-1 surface glycoprotein gp120, while HSV8-N is directed against the gD surface glycoprotein of HSV-2. Both antibodies are produced using a transient expression system in *Nicotiana benthamiana* of human antibody sequences.

The MB66 film is produced by mixing an aqueous solution of VRC01-N and HSV8-N MAbs containing stabilizer excipients, with a solution of PVA, casting the resulting solution in a mold, then drying the cast in an oven. The resulting film is then scalpel-cut in a template guide and packaged in a heat sealed foil laminate pouch, notched for easy opening. Each film contains 10 mg of VRC01-N and 10 mg of HSV8-N (see also Section 10.4 for ingredients and photo).

Preclinical safety and vaginal pharmacokinetic studies

Tissue cross reactivity: LeafBio has carried out tissue cross reactivity studies against a panel of human tissues with VRC01-N and HSV8-N. The panel included the following tissues: adrenal gland, heart, salivary gland, kidney, skin, blood cells, liver, spinal cord, blood vessels, lung, spleen, bone marrow, lymph ode, striated muscle, brain (cerebellum, cerebral cortex), pancreas, thymus, breast, parathyroid, thyroid, colon, peripheral nerve, tonsil, eye, pituitary, bladder, ureter, ovary, fallopian tube, placenta, uterus (cervix, endometrium), testis, and prostate. Although no cross reactivity was observed with HSV8-N, positive staining in several tissues was observed with VRC01-N. The majority of this staining was cytoplasmic, however extracellular staining was observed in renal tissue from two of three sources. In the extracellular proteinaceous material in kidney, rare to occasional staining was observed at the 20 µg/mL concentration of VRC01-N, and rare staining was observed at the 2 µg/mL concentration of VRC01-N, (Table 1).

Table 1: Extracellular VRC01-N Staining Observed in Extracellular Proteinaceous Material of Renal Glomeruli and Tubules

Source	Test Article	
	**20 µg/mL**	**2 µg/mL**
HT1559-1	Occasional	Rare
HT1563-1	Rare	Negative
HT1200-2	Negative	Negative

Considering the low systemic bioavailability of vaginally applied mAbs observed in a previous multi dose study with other anti-viral mAbs (Morris et al., 2010, and Morris et al., 2012), we expect that the cross reactivity of VRC01-N with cytoplasmic elements and with extracellular material in the kidney is not a concern with a topically applied vaginal microbicide. Therefore, the results of the tissue cross reactivity study with VRC01-N and HSV8-N do not suggest a significant risk for toxicity in humans.

Non-GLP primate study of VRC01-N pharmacokinetics and safety in gel format: *Inflammatory markers:* IPCP investigators at Emory (Yerkes Primate Center) have conducted non-GLP vaginal PK and safety studies in cynomolgus monkeys with the VRC01-N (and another Nicotiana-expressed anti HIV antibody, 4E10) in a gel format, using escalating vaginal doses of 1.25 mg, 5 mg, and 20 mg in a HEC gel, each given weekly for four weeks. No inflammation or other adverse effects were observed at any dose on clinical examinations. There were no significant changes in vaginal fluid cytokines observed comparing pre and post dose samples obtained predose, and at 4, 24, and 72 hours (see Investigator's Brochure).

Vaginal VRC01-N antibody levels after gel dosing: Vaginal levels of VRC01-N were assessed at 0.5, 4, and 24 hours post dosing with the gel formulation. Tear Flo filter paper strips were used to obtain weighed samples at multiple intravaginal locations and intervals after vaginal gel administration. Distribution of gel was relatively uniform, and antibody levels declined over time with a half-life between 4 and 12 hours (Figure 5).

Figure 4: Vaginal levels of VRC01-N after vaginal gel format dosing in cynomolgus monkeys

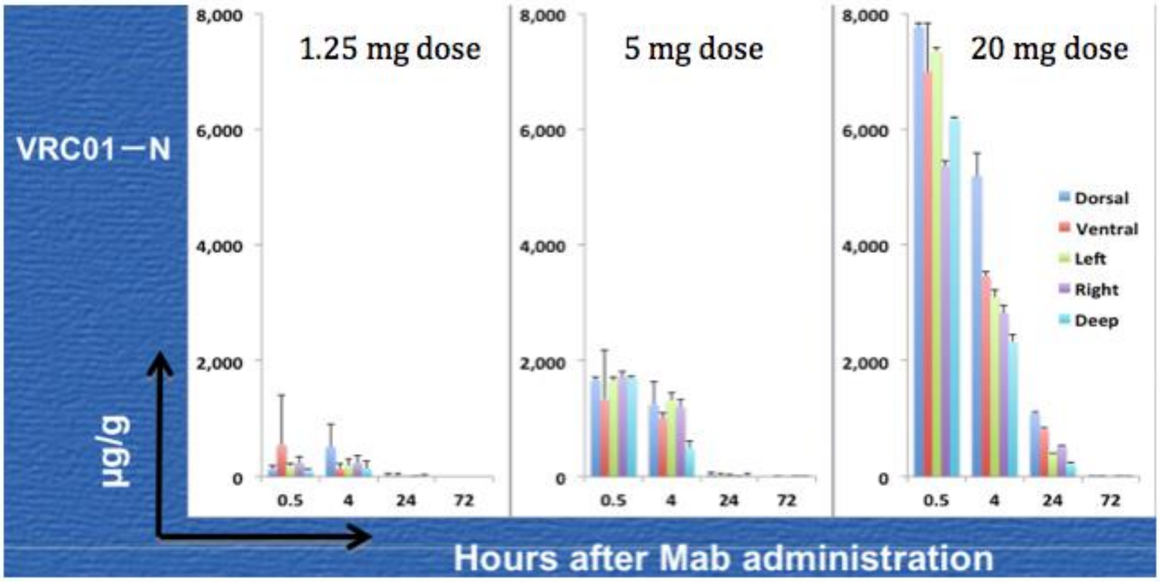

Non-GLP primate study of MB66 film

IPCP investigators at Emory (Yerkes Primate Center) have also conducted a non-GLP vaginal PK and safety study in Rhesus macaques with VRC01-N and HSV8-N in the film format (MB66 film). Rhesus macaques were dosed with ½ dry MB66 film or ½ dry placebo film, alternating one vs three daily doses per week for 4 weeks. Dose levels were thus 5 mg of each antibody per application, and the dosing intensity was 1.7 mg/kg (for comparison, the human dose will be 10 mg of each antibody and approximately 0.33 mg/kg). No adverse events were observed, specifically no vaginal edema, erythema, discharge, or bleeding. Vaginal biopsies taken 24 and 72 hr post dosing were unremarkable. Specifically there were no ulcerations, thrombi, and minimal to no inflammatory infiltrates. Vacuolated cytoplasm was noted in 4 of 54 sections, but in the opinion of the pathologist, these findings are compatible with a progestational effect of the luteal phase of the cycle (most animals were in the luteal phase), and the findings were equally distributed between placebo and MB66 (active) film groups.

Vaginal fluid samples were taken at intervals after dosing for cytokine determinations at 4, 24, and 72 hrs after dosing of MB66 or placebo film. No significant changes were observed as a function of time post dose, nor in comparing active MB66 film to placebo, which showed similar cytokine profiles (see Investigator’s Brochure).

Vaginal pharmacokinetics of MB66 film in Rhesus macaques

Macaques were dosed once, or once daily for three consecutive days with ½ of an MB66 film thus each dose containing 5 mg of VRC01-N and 5 mg of HSV8-N. The film was inserted via a small speculum. At intervals after dosing, pre-weighed Tear Flo filter paper strips were applied to the deep, mid and superficial vagina, and reweighed to determine the sample weight. Antibody levels of diluted samples were determine by ELISA with HIV gp 120 or HSV-2 gD antigen coated wells. Figure 5 plots antibody levels for both mAbs at intervals after a single dose of ½ film and

Figure 6 at intervals after three consecutive daily doses of ½ film, timed after the third dose. During the study some animals were menstruating, and mAb levels were lower and dropped faster in these animals. Vaginal residence half life in non-menstruating animals was between 4 and 8 hours.

Figure 5: Vaginal antibody levels after a single dose of ½ MB66 film in Rhesus macaques (N=6)

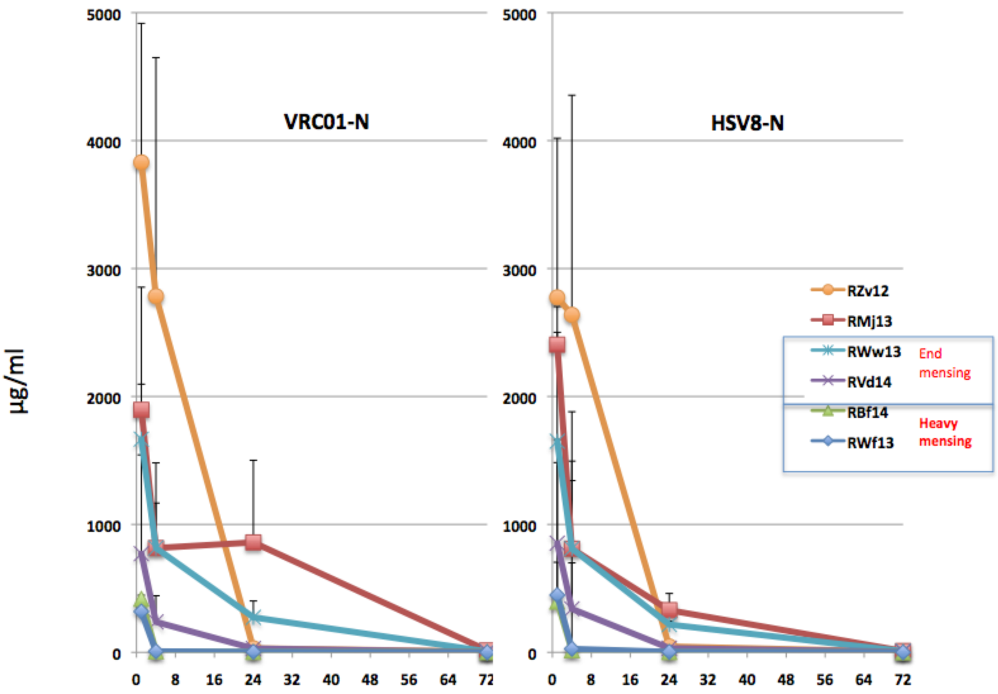

Figure 6: Vaginal antibody levels after three daily doses of ½ MB66 film (N = 6)

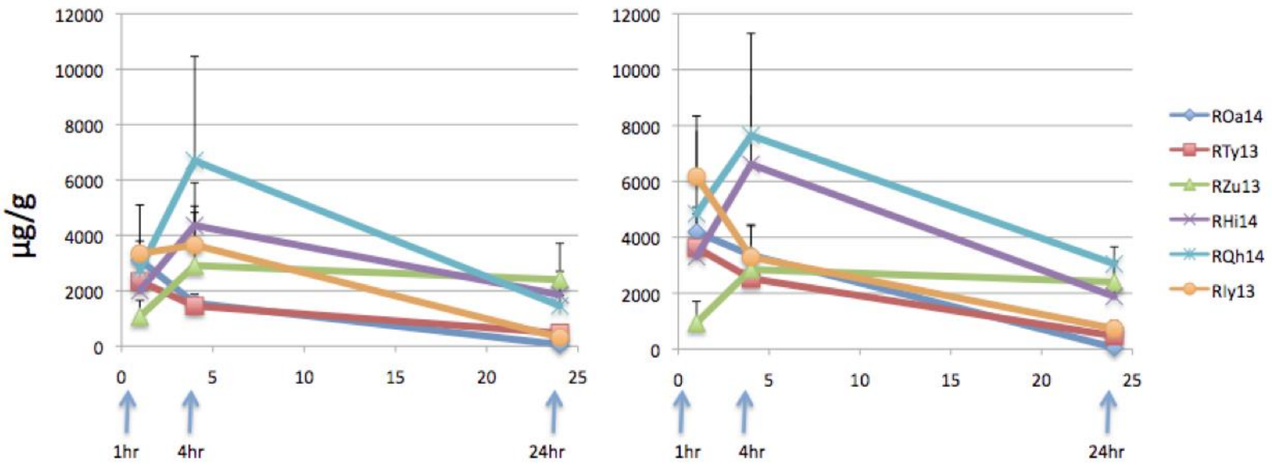

Note: Animals ROa14 and RTy13 (red and dark blue lines) were menstruating during the study.

GLP rabbit vaginal toxicology study

In a 10-day GLP rabbit vaginal toxicology study, both dry film and solubilized film was given to rabbits daily for 10 consecutive days. Groups included sham application, ¼ placebo film, ¼ MB66 dry film (1.66 mg/kg), 1/16th MB66 dry film (0.41 mg/kg), and one full MB66 film solubilized in water (6.7 mg/kg). In 10 days of daily dosing, there were no significant toxicological findings in any group except the ¼ MB66 dry film group. In this group bleeding was observed during the in-life phase in 3 of 9 animals, and histology revealed ulcers, inflammation, and sub-epithelial thrombi in the vaginal mucosa. The lack of findings with high dose (1 full film) in *solubilized* form indicates that the observed toxicity was not directly due to the active agent, the MB66 antibodies, but related to the delivery of the antibodies as the dry MB66 film. Because of the small size of the rabbit vagina, administering the vaginal film in rabbits required administering it as a multi-layer wound-up cylinder. The study pathologist speculated that the tightly wound film may have resulted in high concentrations of antibody over a small area of the rabbit vaginal mucosa. It is also notable that unlike the human vaginal epithelium, the area of the rabbit vagina exposed to film was not covered by keratinized epithelium. All serum samples were below the lower limit of quantitation of the VRC01 ELISA assay (50 ng/mL), thus showing no systemic absorption within the limits of the assay.

GLP rat vaginal toxicology study

In a 14-day GLP rat vaginal toxicology study, daily administration of solubilized MB66 did not produce any toxicologically significant adverse effects at doses up to a maximum of 6 mg/day (3/10ths of an MB66 film, 30 mg/kg, which is the equivalent of 90 times the proposed human dose). All serum samples were below the lower limit of quantitation of the VRC01 ELISA assay (50 ng/mL), thus showing no systemic absorption within the limits of the assay.

As described in Table 2, the No Observable Adverse Event Level (NOAEL) dose of dry film in rabbits is 1.25 times the proposed human dose on a mg/kg basis, and the dose in rabbits that resulted in observable toxicity with dry film was five-times the proposed human dose. In rats the NOAEL level with solubilized film was above the maximum dose of 30 mg/kg (90 times the proposed human dose.

Table 2: Comparison of toxicology results in film studies vs. the proposed human dose

**GLP Rabbit study (N=9)**			
**Film dose**	**Total MAb dose**	**mg/kg**	**Outcome in 10 d rabbit study**
$1/16^{th}$ MB66 film	1.25 mg	1.25 mg/3 kg = 0.41 mg/kg	No observed adverse effects
¼ MB66 film	5 mg	5 mg/3 kg = 1.66 mg/kg	In life: vaginal bleeding in 3 of 9 rabbits<br>Histopath: thrombi, ulcers, inflammation
1 full MB66 film	20 mg	20 mg/ 3kg = 6.67 mg/kg	No observed adverse effects
**GLP Rat study (N=14)**			
**Film dose**	**mg of active**	**mg/kg**	**Outcome in 10 d rat study**
$3/10^{ths}$ MB66 film (solubilized)	6 mg	6 mg/0.2 kg = 30 mg/kg	No observed adverse effects
**Non-GLP Rhesus macaques study (N=5)**			
**Film dose**	**mg of active**	**mg/kg**	**Outcome in 28 d intermittent dosing monkey study**
½ MB66 film	10	10 mg/5.8 kg 1.72 mg/kg	No observed adverse effects
**Proposed dosing in women**			
1 MB66 film	20 mg	20 mg/60 kg = 0.33 mg/kg	

Preclinical efficacy studies

In vitro potency neutralization potency of VRC01

A series of chimeric subtype C HIV-1 viruses were tested, which displayed HIVenv from primary isolate strains cloned into MJ4 backbone. Nicotiana expressed (left) and mammalian cell expressed VRC01 (right) showed similar neutralization potency on TZM-BL cells, and likewise against SHIV162p3 (Clade B). Nicotiana produced VRC01 was also shown to be active against an additional Clade C and against a Clade A construct (Table 3, unpublished, Villinger and Zhao, Emory University). Prior published data shows high and broad potency of mammalian cell expressed VRC01 against multiple HIV-1 strains (Zhao 2010).

Table 3: Potency of VRC01 expressed in Nicotiana vs. mammalian cells against SHIV

	IC50 (µg/ml)	
SHIV Strain	Nicotiana VRC01-N	Mammalian VRC01
109 M13 (Clade C)	0.445	0.442
109 F4 (Clade C)	0.296	0.773
53 F2.13 (Clade C)	0.465	4.937
53 M12 (Clade C)	1.060	>5
135 F72a (Clade C)	4.895	4.612
SHIV162p3 (Clade B)	0.972	1.109
2873 (Clade C)	5.561	
KNH1144p2 (Clade A)	0.860	

Neutralization potency of HSV8

HSV8, a fully human anti-HSV glycoprotein D (gD) Ab has been shown to neutralize a panel of six low passage clinical isolates of HSV-1 and HSV-2 (de Logu *et al.*, 1998).

In vivo protection after vaginal dosing of VRC01-N gel format in cynomolgus macaques (N=5)

Challenge studies in the cynomolgus macaque repeated-low-dose-challenge model were performed at Emory University/Yerkes Primate Center (Zhao, 2013). Five weekly viral challenges were given in conjunction with dosing at each of three Mab dose levels of either VRC01-N or 4E10-N (5, mg, 12.5 mg or 20 mg), with the antibodies given in HEC gel 30 min before each challenge. A 2 mL dose of gel containing 10 mg/mL of Mab in hydroxyethylcellulose gel (thus 20 mg total dose) dosed into five macaques provided complete protection against 5 weekly challenges with SHIV163p3 (2000 $TCID_{50}$, titered on Rhesus

macaque PBMC). In contrast all five animals dosed with vehicle control gel became infected after between two and three weekly challenges. Reducing the antibody concentration 4-fold (to 2.5 mg/mL, thus 5 mg total dose) reduced but did not eliminate protection: three animals resisted five challenges, one animal was infected after one challenge, and one after two challenges (Figure 7). The 4E10-N antibody provided moderate protection at the 20 mg dose level (Figure 7).

Figure 7: Protection of cynomolgus macaques by VRC01 & 4E10 against SHIV challenge

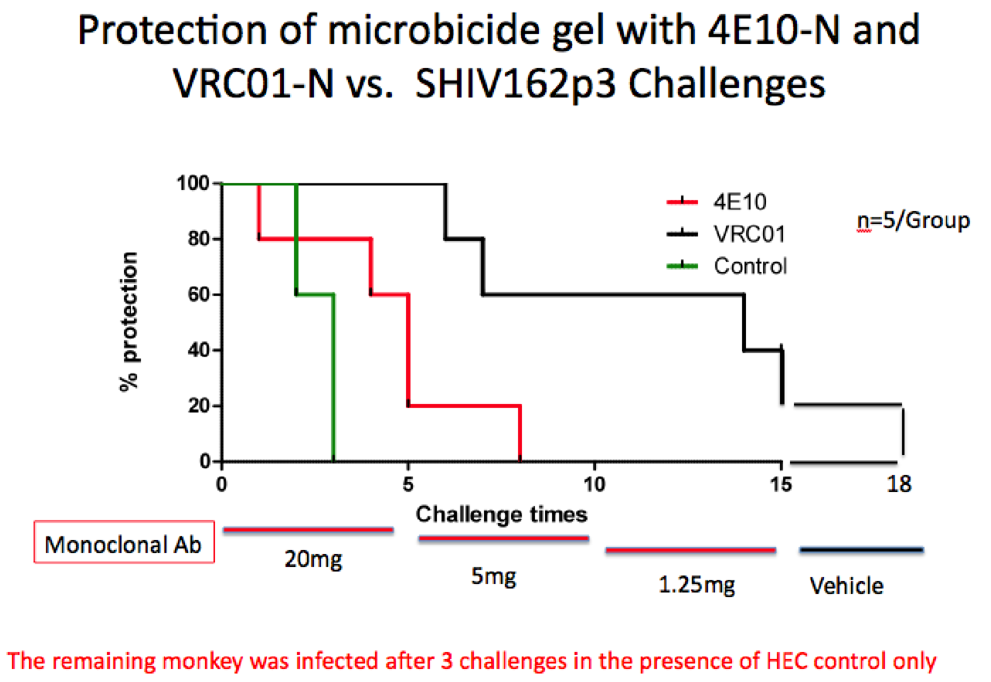

In vivo protection of unformulated HSV8-N in the mouse vaginal challenge model

IPCP investigators at Johns Hopkins using the HSV-2 mouse vaginal challenge model (Zeitlin 1996, Zeitlin 1997). Animals treated with Depo Provera to synchronize their estrus cycle and increase their sensitivity to infection were challenged with 10 animal ID_{50} of HSV-2 strain G, after vaginal dosing of HSV8-N in buffer (n = 20 for each of six concentrations between 0.001 and 1 mg/mL). Infection was diagnosed by culture of vaginal lavage fluid on human foreskin fibroblasts. HSV8-N provided dose dependent protection against vaginal transmission, with an in vivo EC_{50} of 40 ug/mL for HSV8-N, and an EC_{90} of approximately 1 mg/mL (Figure 8, R. Cone unpublished), similar to a previous study with mammalian expressed HSV8 (Zeitlin *et al.*, 1998).

Figure 8: Dose response curve for HSV8-N in vivo protection against HSV-2

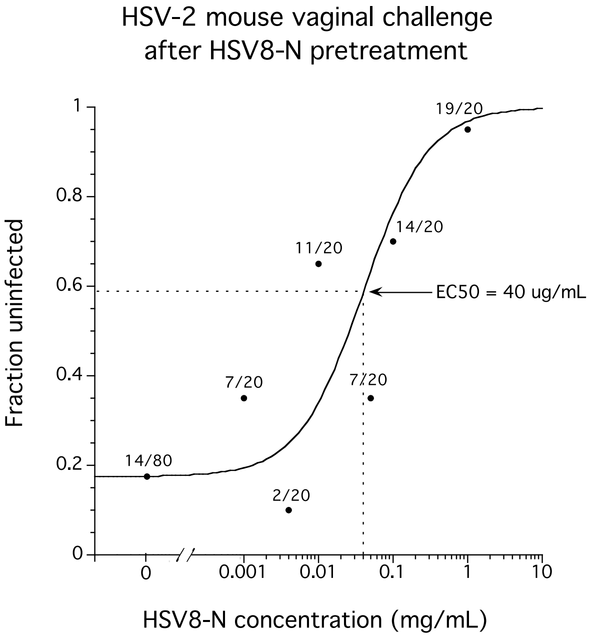

6.2 RATIONALE

6.2.1 Product Rationale

<u>Film product format</u>

The film format was chosen for multiple reasons. Adequate long-term stability of MAbs cannot be achieved in non-solid dosage forms (e.g., gels, creams, or ointments), whereas the film format provides adequate long term stability. Oral and vaginal films are established mucosal dosage forms and provide compactness (allowing for discretion), portability, and convenience. Contraceptive film has been found to be acceptable (Steiner *et al.*, 1995; Visness *et al.*, 1998; Raymond *et al.*, 1999), with women reporting comfort, ease of use, increased lubrication, and low side effects. Film formulations constitute a minimal added vaginal volume, and are expected to reduce the degree of vaginal discharge, which has reduced the acceptability of gel-based microbicides, especially when applied in daily non-coital regimens.

Despite the potential advantages of the film vaginal dosage form, difficulties with proper insertion of a dapivirine vaginal film have recently been reported (Bunge *et al.*, 2014), wherein 5 of 29 women appear to have inserted the film to an inadequate depth. These results may be attributable to the general unfamiliarity of women with this dosage form, stickiness of the film especially if the inserting finger is not completely dry, and insufficient emphasis in dosing instructions on the need for full-depth insertion. In light of these observations, participants in the present study will have intensified insertion instructions using a video demonstration (see

Section 10.3), and will practice insertion of the film into both a transparent rigid pelvic model and into a soft, opaque, silicone vaginal model, with emphasis on the need for full depth insertion. IPCP investigator Morrow's behavioral sciences group at Brown University has experience from other intravaginal film studies, and will devise the training, and instruct the clinical staff in the training methods.

Selection of MAbs and Dosage Form

The MB66 drug product is a vaginal film containing two broadly neutralizing MAbs, an anti-HIV-1 MAb (VRC01-N) and an anti-HSV-2 MAb (HSV8-N). When formulated in a gel, VRC01 protected seven of nine humanized mice (RAG-hu) from HIV infection (Veselinovic *et al*., 2012). MabGel, a multi-Ab gel (containing three other anti-HIV MAbs, 4E10, 2F5, 2G12), was shown to be partially protective in a macaque SHIV-1162P3 vaginal challenge model (Moog *et al*., 2014). In a phase 1 trial of MabGel, the product was shown to be safe (Morris *et al*., 2010).

Unformulated HSV8, a fully human anti-HSV glycoprotein D (gD) Ab which neutralizes a range of low passage clinical isolates of HSV-1 and HSV-2 (de Logu *et al*., 1998), provided 100% protection at 100 µg/ml in a mouse/HSV model (Zeitlin *et al*., 1996; Zeitlin et al., 1997). In another study, complete protection against vaginal challenge with an unformulated anti-HSV gD Ab (produced in soy plants and mammalian cells) required approximately 1 mg/ml for both expression methods (Zeitlin *et al*., 1998). Controlled release of anti-HSV antibodies from ethylene vinyl acetate (EVA)-based vaginal rings demonstrated one week of protection in the HSV/mouse model (Sherwood *et al*., 1996), providing evidence that sustained release of antibodies from daily non-coital dosing with formats such as thin films could provide long-term protection.

Dose rationale

The dose of VRC01-N and HSV8-N in the MB66 film was chosen based on in vivo protection studies. Both antibodies inactivate a broad range of clinical isolates in vitro with EC_{50} typically at or below 1 ug/mL. For in vivo protection by microbicides, concentrations of several hundred to 1000-fold higher are typically required (Zeitlin *et al*., 1997).. A concentration of 10 mg/mL of VRC01-N in hydroxyethylcellulose gel dosed into five macaques 30 minutes before each challenge provided complete protection against 5 weekly challenges with SHIV163p3 (see Figure 7 above). Likewise, IPCP investigators at Johns Hopkins using the HSV-2 mouse vaginal challenge model (Zeitlin 1996, Zeitlin 1997) found an animal EC_{50} of 40 ug/mL for HSV8-N, with an EC_{90} of approximately 1 mg/mL (see Section 6.1.3). With these SHIV and HSV-2 data in mind, a dosage level of 10 mg for each antibody per MB66 film was chosen, with the expectation that this will provide an intravaginal concentration of ~10 mg/mL before (in an estimated precoital vaginal fluid volume of 1 mL, (Owen and Katz, 1999)) and ~2 mg/mL after intercourse (addition of 3-4 mL ejaculate). This concentration is near the maximal dose that can be formulated into a film of this size and composition. We have chosen to use this near maximum Mab dose in order to improve the probability of robust protection. We have a high expectation of safety for these antibodies, which are fully human amino acid sequences with glycosylation patterns very similar to those of human antibodies (see Section 6.1.3), and directed

against pathogen rather than human epitopes. We believe such antibodies justify a strategy of choosing a dose near the practical limit in order to improve potency and duration of action.

Rationale for multiple antibodies: MB66 as an "MPT"

Multipurpose Prevention Technologies (MPTs) are intended to simultaneously address multiple sexual and reproductive health needs, including contraception, and HIV and other STI prevention. Conceptually, women could be protected against multiple risks, even if their intention was to address just one perceived health need. MPT products may help alleviate the heavy health and economic toll of unintended pregnancy and sexually transmitted infections (STIs) if women have the option to understand, purchase, store, and use fewer products to maintain sexual and reproductive health. MPTs may include rings, diaphragms, gels, films, vaccines or drug combinations.

First generation candidates for MPTs consist primarily of combinations of commercially available drugs. Future generations of MPT candidates are likely to include proteins/peptide-based molecules as drugs (Dereuddre-Bosquet *et al.,* 2012; Kouokam *et al.,* 2011, Lagenaur *et al.,* 2011; Lagenaur *et al.*, 2010), and vaccines (Diekman *et al.*, 1999; Walker *et al.*, 2011). MAbs are protein-based MPT candidates that are specific for their target, but can be multipurpose when combined to target an array of sexually transmitted pathogens. As discussed above, the MB66 film is intended to protect against HSV and HIV-1 transmission through the combination of the two MAbs (HSV8 and VRC01). Considering the morbidity, chronicity, and incurability of HSV genital infection, and its substantial role as a cofactor increasing susceptibility to HIV acquisition, it is among the highest priorities as an addition to HIV protection in an MPT product. Further, statistical modeling suggests that MPT products like MB66, which prevent the transmission of more than one STI, may increase the likelihood that women will adhere to their use (Harrison, 2013; Holt et al, 2010).

6.2.2 Trial design rationale

Rationale for dose escalation via dosing duration

We have elected to escalate the exposure level based on the duration of dosing (in Segment A followed by Segment B) rather than by altering the concentration of antibody per film, for several reasons. As a practical matter, the expense of manufacturing and characterizing films with three different concentrations of the two Mabs is resource intensive and beyond the means of our program. Second, the loading level of 10 mg of each of the two antibodies is very near the practical loading limit, and we are unable to increase the dose substantially without enlarging the film, or inserting a second film, both of which may result in excessive time require for hydration of the film in the vagina.

Rationale for daily dosing in Segment B

In addition to providing a dose frequency escalation and thus mimicking intensive precoital use of the MB66 film, the seven sequential doses given in Segment B also serve to explore an alternative dosing regimen, namely, daily, non-coital use, including assessments of the effect of repeated dosing on antibody pharmacokinetics and distribution.

Rationale for vaginal sampling methods and timing

Tear Flo sampling: We have chosen the Tear Flo filter paper (previously marketed as Sno Strip) sampling method (Quesnel et al., 1997; John GC et al., 2001; Sherlock et al., 2006; Bennetto-Hood et al., 2009) for our vaginal PK measurements instead of cervicovaginal lavage (CVL) or higher capacity absorptive sampling devices (swabs or Weck-Cel sponges) based on the following rationale: First, our goal is to sample multiple discrete locations in order to observe the extent of distribution of the MAbs in the vagina as a function of time since dosing, and to test the hypothesis that repeated daily doses will improve the extent of MAb distribution over that observed after a single dose. To this end we want to obtain a limited sample volume, taken over multiple limited sampling areas to avoid depletion of MAb by the sampling method (as would occur with CVL or larger capacity sponge sampling devices), thus minimally perturbing residual vaginal antibody and allowing for repeated sampling of the same individual. Second, unlike CVL sampling the Tear Flo method allows for a close estimate of the sampled volume and thus the subsequent dilution factor. Third, Tear Flo filter paper strips have been widely used for vaginal research, including sampling of immunoglobulins, antiretrovirals, and viral load measurements (Quesnel, 1997; Sherlock, 2006; Bennnetto-Hood, 2009; John, 2001). We are aware that despite use of Tear Flo sampling, some depletion of vaginal antibody levels will occur due to sequential sampling of the same individual, but expect this to be sufficiently modest in effect to provide an adequate preliminary picture of antibody persistence after dosing in this Pre-Phase I study. Tear Flow samples will be tested for VRC01-N and HSV8-N antibody levels (in validated ELISA assays using Intertek Pharmaceutical Services SOPs to GLP standards). (Intertek Pharmaceutical Services 3985 Sorrento Valley Blvd., Suite C, San Diego, CA 92121).

Effect of sampling procedures on film retention and antibody levels: Other aspects of our procedure are recognized to also have some potential for depletion of vagial mAb levels. The speculum required to obtain localized samples from multiple sites has the potential to remove antibody or partially hydrated film. We will attempt to minimize this by removing adherent film or fluid from the speculum with a wooden tongue depressor and tip the speculum to maximize vaginal retention of fluid during speculum withdrawal.

CVL sampling and timing: CVL sampling is required for assessments for which Tear Flo sampling gives inadequate volumes, namely for viral neutralization studies and immune mediators. CVL sampling will be delayed until after Tear Flo sampling has been completed at the 24 h post dosing time point, a time when data adequate to calculate the vaginal retention kinetics from the 1, 4, and 24 hours Tear Flo sampling without interference from CVL sampling, has already been obtained. An estimate of the amount of film inadvertantly removed, if any, will be recorded.

Rationale for non-exclusion of HSV seropositives

Participants will not be pre-screened for HSV-2 (or HSV-1) antibodies (to detect for infection with HSV-2 or HSV-1). The resources available for screening/recruitment in this Pre-Phase I study with IPCP funding are not adequate to allow screening out both HSV-2 and HSV-1 seropositive individuals, together estimated to be approximately 50% of the expected otherwise eligible study candidates based on both national surveys and on experience at studies based at

Miriam Hospital. Screening out only HSV-2 positive women will not be helpful since both anti-HSV-1 and HSV-2 endogenous antibodies will both bind in the ELISA assay. We strongly expect both VRC01-N (anti-HIV) and HSV8-N (anti-HSV) antibodies to have very similar absorption, so data on VRC01-N will provide strong preliminary evidence of the level of systemic exposure to both antibodies. Moreover, approximately half the participants are expected to be HSV double seronegative (negative for both serotypes) even without screening, and will contribute substantial direct data on HSV8-N absorption. Regarding vaginal samples, vaginal levels of anti-HSV antibody are very low (generally sub-microgram/mL) even in HSV seropositive individuals (Wang, 2014). Considering these low baseline values, we therefore expect little difficulty assessing the vaginal PK at levels clinically relevant to our criteria for likely protection (~100 ug/mL), even in HSV seropositive participants.

Rationale for choice of HIV tests in a study of VRC01-N

An anti-HIV-1 gp120 antibody (VRC01-N) is being vaginally applied in this study. Although the bioavailability of vaginally applied antibodies has been found to be low (see Section 6.1.3), there a theoretical risk that small amounts of antibody may be absorbed and might be sufficient to cause a false positive HIV antibody test. For this reason we have chosen to use the OraQuick Rapid HIV 1/2 test for the HIV antibody testing done in this study (at baseline and followup) because the test does not include an HIV gp120 target. Although not being used in the present study, there are some HIV antibody tests that do use HIV gp120 target antigen. There is a theoretical risk of false positive results with HIV antibody tests that do contain gp120. However, even these tests appear not to react with VRC01 antibody. The University of Washington Virology Specialty Laboratory (UW-VSL, Dr. Robert Coombs), in conjunction with the HVTN and the VRC/NIH, has tested HIV uninfected human plasma samples spiked with VRC01 at 200 mcg/mL, 50 mcg/mL, 12.5 mcg/mL, 3.1 mcg/mL and 0.8 mcg/mL, and did not observe positive test results in several standard antibody-based HIV-1/2 diagnostic tests used in the US (personal communication Dr. John Hural, HVTN/Fred Hutchinson Cancer Research Center, Seattle, WA, USA). Additional testing is being done in those and other trials of systemically administered VRC01, and we will rely on information from those trials rather than make further assessments of possible interactions with VRC01-N and such HIV blood tests during the present pre-Phase I study.

6.3 STUDY HYPOTHESES

The study will test the following hypotheses:

1. That the MB66 film will be safe as compared to placebo vehicle only film;
2. That the MB66 film will show adequate dissolution, distribution, and residence time for pre-coital use;
3. That antibody distribution will be enhanced and residence time will be lengthened by daily administration of film compared to a single film administration.

7. STUDY OBJECTIVES

7.1 PRIMARY OBJECTIVE

To evaluate the safety of the MB66 film

7.2 SECONDARY OBJECTIVES

To measure pharmacokinetic parameters including:

- The rate of MB66 film dissolution
- The vaginal concentrations of the MB66 antibodies
- The degree of systemic absorption of the MB66 antibodies

7.3 EXPLORATORY OBJECTIVES

- To assess the antiviral effect of the MB66 antibodies *ex vivo* in cervicovaginal lavage fluid from participants after dosing with MB66 film
- To compare the effects of MB66 and placebo films on the cervicovaginal microbial environment using pH, Nugent score, and bacterial ribosomal DNA PCR
- To compare the effect of MB66 and placebo films on cervicovaginal immune mediators by Luminex and ELISA assays
- To assess the acceptability of the MB66 vaginal film after 7 days of use

8. STUDY DESIGN

8.1 OVERVIEW

This is a single center, Pre-Phase 1, randomized, single blind, placebo-controlled, two-segment study to assess the safety of the MB66 vaginal film. After appropriate screening, approximately 43 healthy women will be enrolled for a target evaluable population of 38 healthy, HIV uninfected women 18-45 years of age.

The study will be divided into two sequential Segments. The first, Segment A, is a single-arm, single-dose, open label design. The 8 participants will receive a single dose of one full MB66 film. After dosing, subjects will be asked to maintain sexual abstinence and will be evaluated in person on Day 1 (24-hours post MB66 administration), by telephone on Day 3 or 4 and in person on the Day 6-10 Exit Visit, after which subjects will be allowed to resume sexual activity with study-provided condoms until three weeks after last exposure to film (to avoid male exposure to residual drug product). A placebo arm was not included in Segment A, because the very low risk of toxicity of the placebo film makes it unlikely that toxicity observed in Segment A would be wrongly attributed to the active agents (mAbs) in the MB66 film when it was instead due to the vehicle. This conclusion is based on the known tolerance of the very similar vehicle used in a commercial PVA-based vaginal film (VCF), and the absence of toxicity of the MB66 placebo

film in the very sensitive rabbit model after substantially higher dosing intensity and duration (see Section 6.1.3). Figure 1 (Section 5) shows a diagram of the Segment A study schema.

Completion of Segment A and a safety review of Segment A adverse events will trigger the initiation of Segment B (Figure 2, Section 5), a repeat dose, randomized, two arm, single-blind, placebo-controlled design. Subjects will be randomized 1:1 into two groups (15 evaluable subjects per group) and be treated once daily with either 1 MB66 film or 1 vehicle control placebo film for seven days. For five days before, and for 7 days after the dosing period, subjects will be asked to maintain a period of sexual abstinence. Subjects will be evaluated in person on Day 0 (at 1 and 4 hours post MB66 in-clinic dosing), again on Day 1 (24 hr post dosing), by telephone on Day 3-4, and evaluated again in person on Day 7-8, after which, subjects will be allowed to resumed sexual activity with condoms required until three weeks after last film insertion. Subjects will be evaluated a last time on the Day 12-16 Exit Visit.

8.1.1 Study Endpoints

Primary Endpoint:

Incidence of Grade 2 or higher Adverse Events deemed related to study product, graded according to the *Division of AIDS (DAIDS) Table for Grading the Severity of Adult and Pediatric Adverse Events, Version 2.0, Nov 2014* (Reference 39), and the Female Genital Grading Table for Use in Microbicide Studies (Addendum 1 to the DAIDS Table for Grading Adult and Pediatric Adverse Events, Version 1.0, December 2004 [Clarification dated August 2009]), (see Reference 40). Safety will be assessed throughout the study by monitoring of adverse events, vital signs, physical examinations, and/or clinical laboratory values, and review of concomitant medications and procedures. The number of Grade 2 or higher AEs will be the primary endpoint, but all AEs will be reviewed by the PSRT and analyzed regardless of severity or relationship.

Secondary Endpoints

Pharmacokinetic (PK)

- Naked eye visual assessment during speculum exam of the degree of film dissolution at 1, 4, and 24 hours after dosing
- Concentrations of MB66 antibodies in vaginal fluid sampled with Tear Flo (filter paper) wicks, and measured by ELISA
- Concentrations of MB66 antibodies in serum by ELISA

Exploratory Endpoints:

Ex vivo antiviral effect

- Assays of the ex vivo viral neutralization of HIV-1 and HSV-2 in cervicovaginal lavage fluid after MB66 dosing

Cervicovaginal microbial environment

- Vaginal pH
- Nugent score on Gram stained vaginal smears
- Vaginal microbiome assessment by polymerase chain reaction (PCR) for bacterial ribosomal DNA (samples stored for later analysis)
- Levels of immune mediators in cervicovaginal lavage fluid measured by Luminex and ELISA immunoassays

Immune modulators

- Assays of cytokines and other soluble immune mediators in CVL

Acceptability

- Determine acceptability of MB66 film by audio computer administered structured interview after 7 days of MB66 or placebo film.

8.2 Description of Study Population

The study population will consist of healthy, low risk HIV-uninfected women who meet the criteria outlined in Sections 9.2 and 9.3.

8.3 Time to Complete Accrual

Accrual is expected to be completed in approximately 12 months, with study completion in approximately 14 months.

8.4 Study Groups

8.4.1 Segment A:

Enrollment of 8 evaluable participants:

- Segment A participants: 1 MB66 film

8.4.2 Segment B:

Randomization with be 1:1 amongst the two groups of 15 evaluable participants each:

- Segment B Study Arm 1: 1 MB66 film
- Segment B Study Arm 2: 1 Vehicle control placebo film

8.5 EXPECTED DURATION OF PARTICIPATION

The duration of study participation per woman is expected to be approximately 5-9 weeks, including the screening period. Participation from enrollment to Exit Visit is 1 week for Segment A and 2 weeks for Segment B, plus an additionl 2 weeks of post exit mandated condom use.

8.6 STUDY SITE

The study will be conducted at a single study site: The Miriam Hospital, Providence, RI.

9. STUDY POPULATION

9.1 SELECTION OF STUDY POPULATION

The inclusion and exclusion criteria in this section will be used to ensure the appropriate selection of study participants.

Once a participant is enrolled, the study site will make every effort to retain her in follow-up to minimize possible bias associated with loss-to-follow-up (see Section 9.6). Participants may withdraw from the study at any time. No justification for such a decision is required. Participants must also be withdrawn from the study if the Investigator considers it in the best interest of the participant. The date and reasons for study withdrawal must be recorded in the case report form (CRF) and in the participant's study source documents. If possible, all final study assessments and procedures, such as a complete, final examination, should be performed on all participants who choose to withdraw or are withdrawn from the study. Whether due to loss of follow-up or inability to use study product, participants who are non-evaluable (see Section 14.2) will be replaced.

9.2 INCLUSION CRITERIA

Women must meet all of the following criteria to be eligible for inclusion in the study:

1) Age 18 through 45 years (inclusive) at screening
2) Able and willing to provide written informed consent to be screened for and enrolled in the study
3) Able and willing to provide adequate locator information at screening
4) HIV-uninfected based on testing performed by study staff at screening (per algorithm in Appendix 2)
5) In general good health as determined by the site clinician
6) Agree to abstain from any vaginal insertions, including products, douches, devices such as sex toys, or penile or oral intercourse from 5 days prior to Visit 2 (Enrollment Visit) until

the final Study Visit (one week after last dose of study film). Only tampons during menses and clinically indicated speculum exams are allowed.

7) Agree to use condoms provided by the study staff from one week after last use of study film until three weeks after last use of study film
8) Willingness to undergo all study-related assessments and follow all study-related procedures
9) Be currently using an effective method of contraception at enrollment (used continuously and with good compliance for the past 60 days as determined by participant self-report) with plans to continue use throughout the study period. Acceptable methods include any hormonal method (except vaginal ring); intrauterine device (IUD) inserted at least 90 days prior to enrollment; female sterilization; abstinent from sexual activity with male partner for the past 60 days; sexual activity with vasectomized partner; engages in sex exclusively with women.
10) For participants 21 and older, a Pap result in the 36 calendar months prior to the Enrollment Visit consistent with Grade 0 according to the Female Genital Grading Table for Use in Microbicide Studies Addendum 1 to the DAIDS Table for Grading Adult and Pediatric Adverse Events, (see Reference 40) satisfactory evaluation with no treatment required of non-Grade 0 Pap result per American Society for Colposcopy and Cervical Pathology (ASCCP) guidelines or per local standard of care, within the last 36 calendar months prior to enrollment.

    Note: For participants aged 18-21, a Grade-0 or adequately evaluated abnormal Pap smear is not required as the American Society for Colposcopy and Cervical Pathology recommends initiating screening at age 21.

11) At screening and enrollment, agrees not to participate in other research studies involving drugs, medical devices, or vaginal products while enrolled in this trial

9.3 EXCLUSION CRITERIA

Women who meet any of the following criteria by participant report will be excluded from the study. Of note, the study is limited to pre-menopausal women with an intact uterus because the mucosal immune environment differs substantially between pre- and post-menopausal women. Therefore, inclusion of post-menopausal women would introduce heterogeneity into the population.

1) Menopausal at screening (as defined as amenorrhea or irregular periods for one year or more without an alternative etiology)
2) Hysterectomy
3) Known adverse reaction to any of the study products (ever)
4) Known adverse reaction to latex (ever)
5) Non-therapeutic injection drug use in the 12 months prior to screening

6) Surgical procedure involving the pelvis in the 90 days prior to screening (includes dilation and curettage or evacuation, and cryosurgery; does not include cervical biopsy for evaluation of an abnormal pap smear or IUD placement)
7) Participation in a drug, spermicide and/or microbicide study in the 30 days prior to screening or anticipated participation in an investigational drug study in the next 8 weeks
8) Pregnancy within 90 days prior to screening
9) Lactating
10) Use of a diaphragm, NuvaRing, or spermicide for contraception
11) As determined by the PI, a degree of menstrual cycle irregularity that would make it difficult to schedule follow up visits without interruption by menses
12) Active sexually transmitted infection or documented treatment of sexually transmitted infections in the last 6 months, including, but not limited to: chlamydia, gonorrhea, syphilis, trichomonas, cervicitis or pelvic inflammatory disease, or currently active HSV lesions or other sores. (Participants seropositive for or with a history of HSV without current active lesions will not be excluded.)
13) Women who by history engage in condom-less intercourse with HIV-infected partners, or who exchange sex for money, shelter, or gifts, or who in the opinion of the investigators, may be at risk for HIV acquisition during the duration of the study
14) More than one sex partner within the past 3 months
15) Current sexual partner known by participant to be HIV seropositive
16) Current or planned use of pre-exposure prophylaxis against HIV infection
17) Currently active genital HSV lesions, or other genital tract epithelial disruption or inflammation
18) Current or episodic use of anti-herpes suppressive therapy
19) Urinary tract infection, symptomatic candidiasis, or symptomatic bacterial vaginosis within 14 days of enrollment, or currently residual symptoms thereof

    Note: women with these infections at screening can be enrolled after treatment and resolution of the infection.

20) Antibiotic or antifungal therapy (vaginal or systemic) within 7 days of enrollment
21) Use of systemic immunomodulatory medications within 4 weeks of enrollment
22) Menses or other vaginal bleeding at the time of enrollment* or expecting menses in the 10 days after enrollment (Segment A participants) or 16 days after enrollment (for Segment B participants)

    **Note: for women with monthly cycles, every attempt will be made to enroll these participants in the first half of their menstrual cycle. Women who have vaginal bleeding at the scheduled Enrollment Visit may return at a different date to be re-examined and*

possibly enrolled provided they are still within the screening window and meet all criteria.

23) Lack of stable living conditions to allow reliable room temperature storage of study product (Segment B participants only)

24) At enrollment has any of the following laboratory abnormalities per the *Division of AIDS (DAIDS) Table for Grading the Severity of Adult and Pediatric Adverse Events, Version 2.0, Nov 2014* (Reference 39):
    - Grade 1 or higher AST or ALT
    - Grade 1 or higher creatinine
    - Grade 2 or higher hemoglobin
    - Grade 1 or higher platelets

    Note: otherwise eligible participants with an exclusionary test may be re-tested once during the screening process.

25) As determined by the Principal Investigator (PI), any subject who has any significant uncontrolled active or chronic cardiovascular, renal, liver, hematologic, neurologic, gastrointestinal, psychiatric, endocrine, respiratory, immunologic disorder or infectious disease, anticoagulation with warfarin or heparin; or any other condition that, in the opinion of the Investigator, would preclude provision of consent, make participation in the study unsafe, complicate interpretation of study outcome data, or otherwise interfere with achieving the study objectives.

9.4 Co-enrollment Criteria

As mentioned in Section 9.3 (Exclusion Criterion #7), participation is prohibited in any drug, spermicide and/or microbicide study in the 30 days prior to screening, as is anticipated participation (co-enrollment) in an investigational drug study in the 8 weeks following enrollment in the present trial.

9.5 Recruitment Process

Participants will be recruited from a variety of sources including the ambulatory clinic of The Miriam Hospital and the surrounding population. Participants will also be referred to the study from other local research projects, research registries, IRB approved advertisements, and other health and social service providers serving the target study population. Recruitment materials will be approved by the Miriam Hospital Institutional Review Board (IRB).

9.6 Participant Retention

Once a participant is enrolled, the study site will make every effort to retain her in follow-up to minimize possible bias associated with loss-to-follow-up. The site will implement the following procedures to enhance retention:

- Thorough explanation of the study visit schedule and procedural requirements during the informed consent process and re-emphasis at each study visit
- Thorough explanation of the importance of all study groups to the overall success of the study
- Collection of detailed locator information at the study Screening Visit, and active review and updating of this information at each subsequent visit
- Use of appropriate and timely visit reminder mechanisms
- Immediate follow-up on missed visits

Participants may withdraw from the study at any time. No justification for such a decision is required. Participants must also be withdrawn from the study if the Investigator considers it in the best interest of the participant. The date and reasons for study withdrawal must be recorded in the case report form (CRF) and in the participant's study source documents. If possible, all final study assessments and procedures, such as a complete, final examination, should be performed on all participants who choose to withdraw or are withdrawn from the study.

The study site will use a participant tracking mechanism to facilitate visit scheduling and timely identification and follow-up on missed visits. Whether due to loss follow-up or inability to use study product, participants who are non-evaluable (see Section 14.2) will be replaced.

9.7 Planned Sample Size

An estimated 43 study participants from a single study center at The Miriam Hospital, Providence, RI will be enrolled to reach 38 evaluable subjects.

10. STUDY PRODUCT

10.1 Identity

**Biologic name:**	VRC01-N monoclonal antibody<br>HSV8-N monoclonal antibody
**Dosage form:**	Vaginal film
**Strength:**	10 mg of each monoclonal antibody per film
**Contract Manufacturer (Drug Substance and Drug Product):**	Kentucky Bioprocessing, Inc.<br>3700 Airpark Dr.<br>Owensboro, KY 43201<br>(Contract Manufacturer for Sponsor Leaf Bio, Inc.)
**Proposed Indication(s) or context of product development**	Topical anti-microbial agent used to reduce mucosal transmission of human immunodeficiency virus type 1 (HIV-1) and human herpes simplex virus (HSV-2)

The study will test the safety of the MB66 film containing the monoclonal antibodies VRC01-N and HSV8-N in MB66 films containing a dose of 10 mg of each antibody in each film. In Segment A, the study clinician will administer a single dose of one MB66 film with a dose level of 10 mg of each antibody. Then in Study Segment B, dosing will escalate to seven daily doses of one MB66 film (10 mg of each antibody daily) tested against the vehicle control placebo.

10.2 REGIMEN

Segment A participant will receive the following regimen:

- 1 MB66 film

Segment B participants will be randomized to one of the following regimens:

- Study Arm 1: 1 MB66 film
- Study Arm 2: 1 vehicle control placebo film

Study films will be provided individually sealed in a lightproof foil laminate (Mylar) pouch. Packaged study film will be stored at 2-8 °C until dispensed and at room temperature once dispensed to Segment B participants.

Table 4: Study Dose Regimen

Study Group	Regimen
**Segment A**	
MB66 film	Single dose of one MB66 film<br>(10 mg VRC01-N and 10 mg HSV8-N) (8 evaluable participants)
**Segment B**	
MB66 film	Seven daily doses of one MB66 film *<br>(10 mg VRC01-N and 10 mg HSV8-N per day) (15 evaluable participants)
Placebo film	Seven daily doses of one placebo film * (15 evaluable participants)

*The Visit 4 target date for Segment B is Day 7, though the window for the visit includes Day 8. Consequently, some participants will receive 8 daily doses, although every effort will be made to schedule participants for Day 7 if feasible, to limit the variation in dosing.

10.3 ADMINISTRATION

A study clinician will manually insert the MB66 film in the clinic for Segment A participants, and Segment B participants will self-administer their first dose of study film in clinic in the presence of the study clinician (to assure themselves and study staff of their ability to insert the film), and then self-administer doses 2-7 at home. Segment B participants will take home their remaining doses in a vial capped with a Medication Event Monitoring System (MEMS) cap, to provide objective data about adherence with and timing of at home dosing, (each opening of the MEMS cap will be electronically recorded for time and date, with data downloaded at Visit 4). An extra dose of study film will be available for each Segment A participant in case of an

initially unsuccessful insertion, and three extra doses provided for each Segment B participant in case of unsuccessful insertion in clinic, at home, or for the possibility of delaying Visit 4 to Day 8 instead of Day 7. *(While the intended duration of exposure for Segment B participants is 7 days, participants who are only able to reschedule Visit 4 on Day 8 post MB66 administration will be allowed to do so. For that group, the total dosing will be 8 days rather than 7. Every effort will be made to bring participants back on Day 7 so as to minimize variation in product exposure. Participants will only be enrolled if they indicate their availability to return on Day 7, but if a scheduling conflict should arise post enrollment, they will be allowed to return to the clinic for Visit 4 on Day 8 after administering an additional dose of film on day 7.)*

For Segment A participants the study will be open label (unmasked). For Segment B, the film identity (active or placebo) will be randomly assigned and assignment will be single blinded (study film identity will be masked to the participants) Reliable blinding of study staff will not be feasible due to the non-identical degree of film translucency of the active and placebo films.

The film is folded once and draped over the index or middle finger of the dominant hand, and advanced into the vagina, initially supported by an adjacent finger and thumb. A commercially available vaginal film (VCF contraceptive film) with size and properties very similar to MB66 film will be used to demonstrate film folding and finger placement, (but will not be placed in the participant's vagina). Detailed training (video instruction, one-on-one instruction, and hands-on training with insertion of VCF film into pelvic models) will be provided so that both the study staff and participants are confident in women's ability to insert the film to full depth within the vagina. The video is the VCF contraceptive film video and shows how to open the packaging, fold the film, position it on a finger, and then insert it into a transparent pelvic model (the video may be viewed at: http://www.vcfcontraceptive.com/howdoesvcfwork.html). As part of the adherence and product use counseling, Segment B participants will be reminded to wash and dry their hands before and after administration, to avoid use of lotions after washing, and to avoid insertion of other vaginal products. They will receive instruction on the MEMS cap system, which will provide objective data about adherence with at home dosing, (each opening of the MEMS cap is electronically recorded for time and date). Vials of appropriate size will be provided to allow storage and removal of the stacked film pouches.

10.4 STUDY PRODUCT FORMULATION

MB66 film: The MB66 film contains 10 mg of VRC01-N anti HIV-1 MAb, and 10 mg of HSV8-N anti-HSV-2 MAb per film. The film (

Figure 9) is a soft, flexible, translucent, 2” x 2” (51 mm x 51 mm) square, with a thickness of 0.002” (0.06 mm) , and a mass of 0.2 grams. The composition of the active film is listed in Table 5.

Figure 9: MB66 film

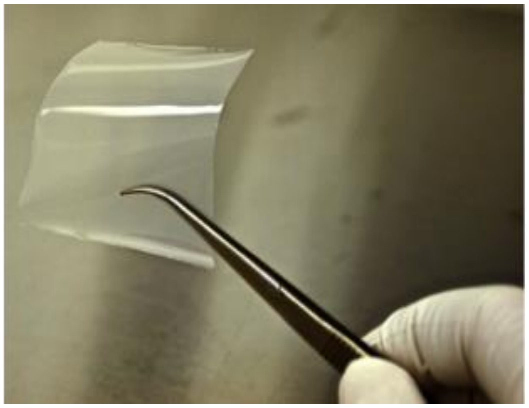

Table 5: MB66 active film composition

Component	% wt/wt)
PVA 8-88	60
Maltitol	25
Histidine	0.1
Polysorbate 20	0.01
HSV8-N	5
VRC01-N	5
Water	5
Total	100

The placebo film is a vehicle control, consisting of the identical excipients and proportions as the corresponding MB66 film formulation, but without the active pharmaceutical ingredients (MAbs). The placebo film is similar in appearance and texture, but is more transparent.

10.5 STUDY PRODUCT STABILITY

Stability testing on the MB66 film (Lot 14MB66-010, mfg 10-JUL-2014), showed that all parameters remained within specifications, with no significant changes in any parameter at 1, 2, 3, and 6-month time points after 4° C storage. Testing parameters included antibody concentration (A_{280}), potency (HIV-1 gp120 and HSV-2 gD ELISA binding), and purity (size exclusion HPLC, and SDS PAGE). This stability study is ongoing. A new clinical lot was manufactured in April 2015, including stability at 25° C, which showed no change in stability parameters after 2 weeks, thus adequate to justify home storage of this duration at room temperature.

10.6 STUDY PRODUCT SUPPLY AND ACCOUNTABILITY

LeafBio, Inc. is the IND holder for MB66 film and will take responsibility to ensure that its contract manufacturer, (Kentucky Bioprocessing, Inc.) provides the appropriately packaged and labeled study product to the Miriam Hospital Research Pharmacy. All study products will be stored in and available to the study staff through the Research Pharmacy.

The Miriam Hospital research pharmacist will maintain complete accountability records of all study products received for this protocol and dispensed to participants. These records will not be available to other members of the research staff. Additional documentation will be required for study film returns, destruction (if applicable) and other related issues as outlined in instructions for DAIDS clinical trials.

Film will be stored at 2-8° C in the Miriam Research Pharmacy until dispensed, and at room temperature in participant’s homes during 6 days of at-home dosing. Participants will be instructed to return any films not used at home to study staff, and returned to the pharmacy for quarantined storage until study completion. All returned or otherwise unused film will be destroyed by the Research Pharmacy after study completion.

10.7 STUDY PRODUCT DISPENSING

Study products will be dispensed only to enrolled participants upon receipt by the Miriam Hospital Research Pharmacy of a written prescription signed by an authorized prescriber.

10.8 ASSESSMENT OF PARTICIPANT STUDY PRODUCT ADHERENCE

Dosing of Segment A participants will be performed by a study clinician. The first dose for Segment B will be placed by the participant in the clinic in the presence of a study clinician. Doses 2-7 for Segment B participants will be administered by participants at home, and adherence will be monitored by the use of MEMS cap devices (see Section 10.3 and 11.1.2.4). MEMS cap data will be used to determine which Segment B participants are sufficiently adherent to be evaluable (see Section 14.2) and will be included in the mITT (per protocol) analysis (see Section 14.7.5).

10.9 CONCOMITANT MEDICATIONS

Enrolled study participants may use non-excluded concomitant medications during study participation. (Excluded medications are: Antibiotic or antifungal therapy (vaginal or systemic), systemic immunomodulatory medications (but not including topical or intranasal steroids), pre-exposure prophylaxis against HIV infection, anti-herpes suppressive therapy and any intravaginal medication other than study film,

All concomitant medications, over-the-counter preparations, vitamins and nutritional supplements, recreational drugs, and herbal preparations will be recorded on the concomitant medications log form.

All participants will be counseled to avoid the use of spermicide and other non-study vaginal products (other than tampons during menstruation and female condoms) while participating in the study. Participants who report use of these products will be counseled regarding the use of alternative methods, but reported use of these products does not require any change in study product administration or follow-up procedures. Condoms provided by study staff will not be coated with any type of spermicide.

11. STUDY PROCEDURES/EVALUATIONS

11.1 SCHEDULE OF PROCEDURES/EVALUATIONS

The schedule for study procedures and evaluations for the entire study is listed in tabular form in Appendix 1. A narrative describing study procedures for each visit of Segment A and Segment B follows below, along with tables describing procedures and evaluations for each visit.

11.1.1 Segment A (Single administration of study film)

11.1.1.1 *Segment A, Pre-Screening*

As part of participant outreach and recruitment strategies, study staff may pre-screen potential study participants (e.g. via telephone) using an IRB approved script. During these interactions, study staff may explain the study to participants and ascertain elements of presumptive eligibility, to be confirmed at the on-site Screening Visit. If the participant is eligible based on responses to a screening script, her name and appointment time will be written on the script. If she signs consent, the telephone script will then become part of her research record. If she does not sign consent, the form will be de-identified.

11.1.1.2 *Segment A Visit 1: Screening Visit*

Screening may take place up to 45 days prior to the Enrollment Visit. Screening procedures may occur over several visits. Written informed consent for screening will be obtained before any screening procedures are initiated. During this visit, women must agree to be sexually abstinent for five days before the Enrollment Visit and for one week after enrollment, and to use condoms

for vaginal intercourse for an additional two weeks after the Exit Visit (Visit 4), thus to approximately Study Day 21. For participants who do not meet the eligibility criteria, screening will be discontinued once ineligibility is determined. If blood counts, AST, ALT, or creatinine are outside exclusionary limits, they may be redrawn and the participant enrolled if the new values are within acceptable limits. Likewise, confirmed cure of treatable non-STI infections before enrollment will allow enrollment of an otherwise excluded participant. If scheduling does not allow enrollment within 45 days of screening, a participant can be rescreened once and enrolled within 45 days of the second successful rescreening. Exclusionary laboratory values will be shared with women, and where further evaluation is clinically indicated, women will be referred to a primary provider. Table 6 below outlines procedures to take place at the Segment A Screening Visit. All vaginal sampling (Tear Flo, swabs, CVL) is via speculum.

Table 6: Segment A, Visit 1 Procedures (Screening Visit)

Component	Procedure/Analysis
**Administrative/Interview**	Participant education<br>Informed consent document<br>Assign screening number<br>Collect contact information<br>Screening interview/eligibility checklist<br>List concomitant medications in log<br>HIV pre- and post-test counseling<br>Abstinence counseling<br>Schedule Visit 2 (Enrollment) appointment if applicable<br>Reimbursement
**Saliva**	Rapid HIV test
**Urine**	Pregnancy test (HCG)<br>Nucleic Acid Amplification Test (NAAT) for chlamydia, gonorrhea, and trichomonas<br>Urinalysis
**Blood**	Complete blood count<br>AST, ALT, Creatinine<br>Syphilis screen (RPR)<br>Baseline serum sample for study product MAb levels<br>Confirmatory HIV testing[Δ]
**Physical Exam**	Vital signs (temperature, blood pressure, and heart rate)<br>Physical exam<br>Height and weight (to be done only at screening)
**Pelvic Exam**	Gynecologic exam including visual inspection of external genitalia, speculum, and bimanual exam[§]<br>Vaginal swab for pH[Δ]<br>Vaginal swab for wet prep[Δ]<br>Pap smear[Δ]

Component	Procedure/Analysis
**CVL***	Baseline anti-viral activity, MAb levels, immune mediators
$^{\Delta}$If clinically indicated<br>§ Bimanual exam is only required at screening<br>*Performed here and in all visits as described in Section 11.5.2	

11.1.1.3 *Segment A, Visit 2: Enrollment*

Subjects who meet the inclusion and exclusion criteria following the Screening Visit may schedule an Enrollment Visit. Care will be taken to attempt scheduling the Enrollment Visit at a time when the participant is not expecting to be currently menstruating or anticipating her menses within 10 days.

After an enrollment eligibility checklist confirms that the participant meets all inclusion/ exclusion criteria based on review of laboratory studies and history, the participant will undergo brief physical exam and a pelvic examination. Pelvic specimens as described below will be collected and the study staff will assess for bleeding and epithelial abnormalities. All vaginal sampling (swabs, and Tear Flo strips) is via speculum.

If no bleeding or other exclusionary findings are evident, the participant will then be enrolled and assigned a Participant ID Number. Subjects with active vaginal bleeding at the Enrollment Visit will not be enrolled at that time, but may be rescheduled for another Enrollment Visit. If vaginal bleeding does not resolve during the screening window period, and the subject expresses interest in enrollment for the study and no additional exclusionary findings are detected, she may be allowed to rescreen.

After enrollment, the study clinician will digitally insert the study film and record the time of insertion. At one and four hours after film insertion, the study clinician will obtain post-dose vaginal Tear Flo samples via speculum for measurement of MAb levels. The clinician will also estimate the degree of film dissolution (conversion from a solid film to a hydrated gel-like consistency assessed by naked-eye visualization via speculum during Tear Flow sampling) as follows: 100%, between 75 and 100%, between 50 and 75%, between 25 and 50%, between none and 25%, and no evident dissolution. Actual times of film dissolution observations and Tear Flo sampling will be recorded here and in all other visits in Segment A and B.

Table 7: Segment A, Visit 2 Procedures (Enrollment)

Component	Procedure/Analysis
**Administrative/Interview**	Update contact information<br>Update concomitant medications log<br>Enrollment interview/eligibility checklist<br>Confirm enrollment eligibility with checklist<br>Enroll and assign Participant ID #<br>Adverse event collection

Component	Procedure/Analysis
	Schedule Visit 3 appointment (24 hours post dose)<br>Abstinence counseling<br>Reimbursement
**Urine**	Pregnancy test (HCG)<br>Dipstick urinalysis
**Physical Exam**	Vital signs (temperature, blood pressure, heart rate)<br>Focused physical exam
**Pelvic Exam**	Gynecologic exam including visual inspection of external genitalia and speculum exam<br>Vaginal smear for Gram stain<br>Vaginal swab for pH<br>Vaginal swab for wet prep[Δ]<br>Pre-dose CVF via Tear Flo sampling (cervical os, ectocervix, mid vagina, distal vagina*)
**Film dosing**	Clinician digital insertion of assigned film dose
**Post dose vaginal PK**	1 hour and 4 hour post dose Tear Flo MAb levels (cervical os, ectocervix, mid vagina, distal vagina), and visual assessment of degree of film dissolution
[Δ] If clinically indicated<br>* One sample at each site, at this visit and at all other Tear Flo sampling visits	

11.1.1.4 *Segment A, Visit 3: Follow-Up Visit Day 1, PK sampling*

This visit will assess safety endpoints and adverse events, and obtain vaginal Tear Flo samples to determine residence half-life and vaginal distribution of the study MAbs, and a blood sample to determine the level of systemic absorption of the study MAbs. Table 8 outlines procedures that will be conducted in Visit 3. All vaginal sampling (swabs, Tear Flo and CVL) is conducted via speculum. At all visits where CVLs are taken, the CVL will be performed *after Tear Flo sampling*. As with Tear Flo sampling, the actual time of CVL sampling will be recorded in this and in all CVL sampling in other visits in Segment A and B.

Table 8: Segment A, Visit 3 Procedures (Follow-Up Visit Day 1)

Component	Procedure/Analysis
**Administrative/Interview**	Visit 3 interview<br>Adverse event collection<br>Update concomitant medications log<br>HIV pre- and post-test counseling<br>Abstinence counseling<br>Schedule Visit 4 appointment<br>Reimbursement
**Urine**	Dipstick Urinalysis<br>NAAT for chlamydia, gonorrhea, and trichomonas[Δ]

Component	Procedure/Analysis
**Blood**	Serum for study product MAb levels<br>Complete blood count$^{\Delta}$<br>Confirmatory HIV testing$^{\Delta}$<br>Syphilis screen (RPR) $^{\Delta}$
**Physical Exam**	Vital signs (temperature, blood pressure, heart rate)<br>Focused physical exam
**Pelvic Exam**	Gynecologic exam including visual inspection of external genitalia and speculum exam<br>Vaginal swab for pH<br>Vaginal swabs for wet prep$^{\Delta}$<br>Vaginal swab for Gram stain<br>CVF via Tear Flo for MAb levels (cervical os, ectocervix, mid & distal vagina), and visual assessment of degree of film dissolution
**CVL**	Anti-viral activity, MAb levels, immune mediators
$^{\Delta}$ If clinically indicated	

11.1.1.5 *Safety Contact 2-3 Days Post Enrollment*

Participants will be contacted via telephone 2-3 days after Visit 3. The purpose of this telephone contact is to assess whether the participant is experiencing any adverse events, and to reinforce the requirement for abstinence. In the event that the clinical staff is concerned that the participant is experiencing an adverse event that needs to be assessed in person, the participant will be asked to return to the clinic for an interim (ad hoc) visit.

11.1.1.6 *Segment A, Visit 4: Exit Visit, 1 Week Post Enrollment*

The final scheduled visit will be targeted for Day 7, seven days post MB66 film administration. The window period will extend from Day 6 to Day 10 post MB66 administration. This visit will assess safety endpoints and adverse events, obtain vaginal Tear Flo samples for study MAb levels, and a blood sample to determine the level of systemic absorption of the study MAbs, and other procedures are conducted as outlined in Table 9. All vaginal sampling (swabs, Tear Flo and CVL) is conducted via speculum. Participant will be reminded they are required to use condoms during any vaginal intercourse until Study Day 21, three weeks after film administration. In the event of an unresolved AE requiring follow-up, an additional ad hoc visit (Section 11.2.4) will be scheduled.

Table 9: Segment A, Visit 4 Procedures (Exit Visit 1 Week Post Enrollment)

Component	Procedure/Analysis
**Administrative**	Visit 4 Interview<br>Adverse event collection

Component	Procedure/Analysis
	HIV pre- and post-test counseling<br>Update concomitant medications log<br>Condom counseling and distribution<br>Reimbursement
**Saliva**	Rapid HIV test
**Urine**	Pregnancy test (HCG)<br>Dipstick urinalysis<br>NAAT for chlamydia, gonorrhea, and trichomonas$^{\Delta}$
**Blood**	Serum for study product MAb levels<br>Complete blood count<br>AST, ALT, Creatinine<br>Confirmatory HIV testing$^{\Delta}$<br>Syphilis screen (RPR) $^{\Delta}$
**Physical Exam**	Focused physical exam<br>Vital signs (temperature, blood pressure, heart rate)
**Pelvic Exam**	Gynecologic exams including visual inspection of external genitalia and speculum exam<br>Visual inspection for any remaining film<br>CVF via Tear Flo MAb levels (cervical os, ectocervix, mid & distal vagina)<br>Vaginal swab for pH<br>Vaginal swab for Gram stain<br>Vaginal swabs for wet prep$^{\Delta}$
**CVL**	Anti-viral activity, MAb levels, immune mediators
$^{\Delta}$ If clinically indicated	

11.1.2 Segment B (Seven daily doses, Active Vs. Placebo film)

After completion of Segment A, the Protocol Safety Review Team (PSRT) (Section 13.8) will review laboratory findings, all AEs, and tabulations of the number and the frequency of ≥ Grade 2 adverse events and ≥ Grade 1 Genitourinary AEs from all Segment A participants. Criteria for ending the study after Segment A and not advancing to Segment B are enumerated in Section 13.8. If the PSRT determines that the observed findings do not indicate excessive risk to proceeding with the multiple dose portion of the study, recruitment of Segment B participants will be authorized. Segment A participants will not be allowed to roll over into Segment B.

11.1.2.1 *Segment B, Pre-Screening*

As described in Section 11.1.1.1, as part of participant outreach and recruitment strategies, study staff may pre-screen potential study participants (e.g. via telephone) using an IRB approved script.

11.1.2.2 *Segment B, Visit 1: Screening Visit*

Screening may take place up to 45 days prior to the Enrollment Visit. Screening procedures may occur over several visits. Written informed consent for the study will be obtained before any screening procedures are initiated. During this visit, women must agree to be sexually abstinent from 5 days prior to the Enrollment Visit until Visit 4 (approximately two weeks after enrollment and one week after last film use), and to use condoms during vaginal intercourse for an additional two weeks, thus until 3 weeks after last film use at approximately Study Day 28. For participants who do not meet the eligibility criteria, screening will be discontinued once ineligibility is determined, except as described in Section 11.1.1.2.

Table 10 outlines procedures to take place at the Screening Visit, for Segment B. All vaginal sampling (swabs and CVL) is done via speculum.

Table 10: Segment B, Visit 1 Procedures (Screening)

Component	Procedure/Analysis
**Administrative/Interview**	Participant education<br>Informed consent document<br>Assign screening number<br>Collect contact information<br>Screening interview/eligibility checklist<br>List concomitant medications in log<br>HIV pre- and post-test counseling<br>Abstinence counseling<br>Schedule Visit 2 (Enrollment) appointment if applicable<br>Reimbursement
**Saliva**	Rapid HIV test
**Urine**	Pregnancy test (HCG)<br>NAAT for chlamydia, gonorrhea, and trichomonas<br>Dipstick urinalysis
**Blood**	Complete blood count<br>AST, ALT, Creatinine<br>Syphilis screen (RPR)<br>Baseline serum sample for study product MAb levels<br>Confirmatory HIV testing[Δ]
**Physical Exam**	Vital signs (temperature, blood pressure, and heart rate)<br>Physical exam<br>Height and weight (to be done only at screening)

Component	Procedure/Analysis
**Pelvic Exam**	Gynecologic exam including visual inspection of external genitalia, speculum, and bimanual exam§<br>Vaginal swab for pH$^{\Delta}$<br>Vaginal swab for wet prep$^{\Delta}$<br>Pap smear$^{\Delta}$
**CVL**	Baseline anti-viral activity, MAb levels, immune mediators
$^{\Delta}$ If clinically indicated<br>[§]Note the bimanual exam is only required at screening	

11.1.2.3 *Segment B, Visit 2: Enrollment*

Subjects who meet the inclusion and exclusion criteria following the Screening Visit may schedule an Enrollment Visit. Care will be taken to attempt scheduling the Enrollment Visit at a time when the participant is not expecting to be menstruating or anticipating her menses within 16 days.

Before performing any study procedures, an enrollment eligibility checklist will confirm that the participant meets criteria including laboratory test results and history. The participant will undergo a brief physical exam and pelvic examination. Pelvic specimens as described below will be collected via speculum and the study staff will assess for bleeding and epithelial lesions. If no bleeding or other exclusionary findings are evident, the participant will then be randomized to study product and assigned a Participant ID Number. The act of randomization is considered the act of enrollment for Segment B.

Subjects with active vaginal bleeding at the Enrollment Visit will not be randomized/enrolled at that time, but may be rescheduled for another Enrollment Visit. If vaginal bleeding does not resolve during the screening window period, and the subject expresses interest in enrollment for the study and no other exclusionary findings are detected, she may be allowed to rescreen.

It is anticipated that the participants' familiarity with vaginal products will vary and that very few of the women will have had any experience with vaginal film insertion. To ensure consistency of application, participants will undergo a standardized training on the study product application after randomization. The training will include viewing a brief video (instructional video demonstrating the handling of VCF film (see description and link under Enrollment Visit for Segment A), a marketed vaginal contraceptive film with similar size and consistency to the MB66 study films). Participants will then then practice insertions using VCF film, first into a transparent pelvic model, and then into a silicone vaginal model. The clinic staff will be immediately available to answer any questions or address any concerns. Participants will then insert their first dose of Study Film vaginally, in the presence of the study clinician, who will assess the participant’s competence in the film insertion procedure (ability to insert fully without visible film at the introitus or on the fingers), and provide additional guidance as needed.

At one and four hours after film insertion, the study clinician will obtain post-dose vaginal Tear Flo samples via speculum for determination of MAb levels in CVF.

Table 11 outlines procedures during the Segment B Enrollment Visit (Visit 2). All vaginal sampling (swabs, Tear Flo strips, and CVL) is done via speculum.

Table 11: Segment B, Visit 2 Procedures (Enrollment)

Component	Procedure/Analysis
**Administrative/Interview**	Update contact information<br>Update concomitant medications log<br>Enrollment interview/eligibility checklist<br>Confirm enrollment eligibility with checklist<br>Enroll and assign Participant ID #<br>Randomization and assignment to Study Film type<br>Adverse event collection (post dosing)<br>Abstinence counseling<br>Study product adherence counseling<br>Schedule Visit 3 appointment<br>Reimbursement
**Urine**	Pregnancy test (HCG)<br>Dipstick urinalysis
**Physical**	Vital signs (temperature, blood pressure, heart rate)<br>Focused physical exam
**Pelvic**	Gynecologic exam including visual inspection of external genitalia and speculum exam<br>Vaginal swab for pH<br>Vaginal swab for Gram stain<br>Swab for microbiome PCR<br>Swab for wet prep$^{\Delta}$<br>CVF via Tear Flo sampling pre-dose (cervical os, ectocervix, mid & distal vagina)
**Film dosing**	Participant digital insertion of first film
**Post-dosing PK sampling**	1 hour and 4 hour post dose Tear Flo MAb levels (cervical os, ectocervix, mid vagina, distal vagina), and visual assessment of degree of film dissolution
$^{\Delta}$ If clinically indicated	

11.1.2.4 *Segment B, Visit 3: Follow-up Day 1 with PK Sampling*

This visit will assess safety endpoints and adverse events, obtain vaginal Tear Flo samples to determine study Mab concentrations and vaginal distribution of the study MAbs, and obtain a blood sample to determine the level of systemic absorption of the study MAbs, and perform other testing as outlined in Table 12. All vaginal sampling (swabs, Tear Flo strips, and CVL) will be performed via speculum. The participant will be given eight doses (thus including two extra

doses) of the study product to be administered at home within a container capped with a MEMS cap to provide an electronic record of opening for disbursing each dose. Testing of the proper functioning of the MEMS cap device will be performed, and instructions for its use will be reviewed, including cautioning against "curiosity openings", and cautioning against removal of more than one film at a time. Participants will be reminded of the need for storage at controlled room temperature, and to avoid high temperature exposures outside the home, for example leaving the films in a vehicle. Instructions on film insertion will be reiterated, and participants will be asked if they have questions about film insertion and if they wish to do further training with the pelvic models.

Table 12: Segment B, Visit 3 Procedures (Follow-up at Day 1)

Component	Procedure/Analysis
**Administrative/Interview**	Visit 3 Interview<br>Adverse event collection<br>Update concomitant medications log<br>Abstinence counseling<br>Study product adherence counseling<br>Schedule Visit 4 appointment<br>Dispense 8 films in MEMS-capped vial and give instructions for MEMS use and daily home dosing<br>Review instructions on film insertion<br>Reimbursement
**Urine**	Dipstick Urinalysis<br>NAAT for chlamydia, gonorrhea, and trichomonas[Δ]
**Blood**	Serum for study product MAb levels<br>Complete blood count[Δ]<br>Syphilis screen (RPR) [Δ]<br>AST, ALT, Creatinine[Δ]
**Physical**	Vital signs (temperature, blood pressure, heart rate)<br>Focused physical exam
**Pelvic**	Gynecologic exam including visual inspection of external genitalia and speculum exam<br>Vaginal swab for pH<br>Swab for microbiome PCR<br>Vaginal swab for Gram stain<br>CVF via Tear Flo MAb levels (cervical os, ectocervix, mid & distal vagina), and visual assessment of degree of film dissolution<br>Vaginal swabs for wet prep[Δ]
**CVL**	Anti-viral activity, MAb levels, immune mediators
[Δ] If clinically indicated	

11.1.2.5 *Safety Contact 2-3 Days Post Enrollment*

Participants will be contacted via telephone 2-3 days (Study Days 3-4) after Visit 3. The purpose of this telephone contact is to assess whether the participant is experiencing any adverse events, to reinforce product adherence and abstinence, and to reiterate the timing of the final film dose 24 hours before the scheduled time of Visit 4. In the event that the clinical staff is concerned that the participant is experiencing an adverse event that needs to be assessed in person, the participant will be encouraged to return to the clinic for an interim (ad hoc) visit.

11.1.2.6 *Segment B, Visit 4: Follow-up Visit Day 7)*

The Visit 4 target date for Segment B is Day 7. The window for the visit also includes Day 8. Consequently, some participants will receive 8 daily doses although every effort will be made to schedule participants for Day 7. When Visit 4 is scheduled, participants will be instructed to insert film on the day before the visit at the same time as the visit is scheduled on the following day, to allow sampling at 24 (+/-4) hours after the last daily dose. During the visit participants will return any unused films, be interviewed for AEs, evaluated for safety endpoints by pelvic exam, and pharmacokinetic sampling of blood and vaginal Tear Flo samples and CVL will be obtained to assess MAb levels and distribution. CVL will also be assayed for antiviral activity, and immune mediators. Additional samples will be used to assess vaginal pH, Nugent score, and microbiome by PCR. All vaginal sampling (swabs, Tear Flo strips, and CVL) will be performed via speculum. Observations on the acceptability of the MB66 study film will be obtained in Segment B participants after they have completed seven daily insertions of the film. Participants will complete a computer assisted structured interview (CASI). We recognize the limitations of acceptability endpoints in participants who have not used the product during intercourse, but expect to learn important new information regarding their experience inserting the film, the degree of vaginal discharge with this product format, and their attitudes toward antibody based products and the plant based manufacturing system.Visit 4 procedures are detailed in Table 13 below.

Table 13: Segment B, Visit 4 Procedures (Follow-up at Day 7)

Component	Procedure/Analysis
**Administrative/Interview**	Visit 4 Interview<br>Adverse event collection<br>Update concomitant medication log<br>Receive returned unused films<br>HIV pre- and post-test counseling<br>Acceptability interview (CASI)<br>Abstinence counseling<br>Schedule Visit 5 appointment<br>Reimbursement
**Urine**	Dipstick Urinalysis<br>Pregnancy test (HCG)<br>NAAT for chlamydia, gonorrhea, and trichomonas$^{\Delta}$

Component	Procedure/Analysis
**Blood**	Serum for study product MAb levels<br>Complete blood count$^{\Delta}$<br>AST, ALT, Creatinine$^{\Delta}$<br>Syphilis screen (RPR) $^{\Delta}$<br>Confirmatory HIV testing$^{\Delta}$
**Physical Exam**	Focused physical exam<br>Vital signs (temperature, blood pressure, heart rate)
**Pelvic**	Gynecologic exam including visual inspection of external genitalia and speculum exam<br>Vaginal swab for pH<br>Vaginal swab for Gram stain<br>Vaginal swab for microbiome PCR<br>CVF via Tear Flo MAb levels (cervical os, ectocervix, mid vagina, distal vagina), and visual assessment of degree of film dissolution<br>Vaginal swabs for wet prep¶
**CVL**	Anti-viral activity, MAb levels, and immune mediators
$^{\Delta}$ If clinically indicated	

11.1.2.7 *Segment B, Visit 5: Exit Visit, Day 14*

The final scheduled visit will be targeted for Day 14, one week after completing seven daily doses of film. The window period for Visit 5 will extend from Day 12 to Day 16 inclusive. During the visit participants will be interviewed for AEs, evaluated for safety endpoints by pelvic exam and safety blood tests. Pharmacokinetic sampling of blood and vaginal Tear Flo samples and CVL will be obtained to assess MAb levels and distribution. CVL will also be assayed for antiviral activity, MAb levels, and immune mediators. Vaginal swabs will be used to assess vaginal pH, Nugent score, and microbiome by PCR. All vaginal sampling (swabs, Tear Flo strips, CVL) will be done via speculum. Participants will be reminded they are required to use condoms during all vaginal intercourse until Study Day 28 (three weeks after their last film administration). In the event of an unresolved AE requiring follow-up, an additional ad hoc visit (Section 11.2.4) will be scheduled. Visit 5 procedures are detailed in Table 14.

Table 14: Segment B, Visit 5 (Exit Visit) Procedures

Component	Procedure/Analysis
**Administrative/Interview**	Visit 5 Interview<br>Adverse event collection<br>HIV pre- and post-test counseling<br>Update concomitant medication log

Component	Procedure/Analysis
	Condom counseling and distribution<br>Reimbursement
**Saliva**	Rapid HIV test
**Urine**	Pregnancy test (HCG)<br>Dipstick Urinalysis<br>NAATS for chlamydia, gonorrhea, and trichomonas$^{\Delta}$
**Blood**	Serum for study product MAb levels<br>Complete blood count<br>AST, ALT, Creatinine<br>Syphilis test (RPR) $^{\Delta}$<br>Confirmatory HIV testing$^{\Delta}$
**Physical**	Focused physical exam<br>Vital signs (temperature, blood pressure, heart rate)
**Pelvic**	Gynecologic exams including visual inspection of external genitalia and speculum exam<br>Vaginal swab for pH<br>Vaginal swab for Gram stain<br>Vaginal swab for microbiome PCR<br>CVF via Tear Flo MAb levels (cervical os, ectocervix, mid & distal vagina), and visual inspection for any remaining film<br>Vaginal swabs for wet prep$^{\Delta}$
**CVL**	Anti-viral activity, MAb levels, and immune mediators
$^{\Delta}$ If clinically indicated	

11.2 CONTINGENT EVENTS AND PROCEDURES

11.2.1 Participants Who Become Infected with HIV

Rapid HIV testing is scheduled at the Screening Visit and at intervals during the study . Trained clinical staff will provide standard post-test counseling and refer subjects who test positive or indeterminate on confirmatory testing during the Screening Visit to a physician for follow-up testing and care. Enrolled participants who have positive or indeterminate Rapid HIV testing results will have standard post-test counseling as well as follow-up confirmatory testing provided by the study (see Appendix 2). Referral for additional counseling related to testing or diagnosis will occur if needed or requested by the participant. Study staff will perform HIV antibody testing at other visits as clinically indicated. Participants who are found to be HIV positive in follow-up confirmatory blood testing will be terminated from the study and referred for care.

11.2.2 Participants Who Become Pregnant

Participants will be counseled to be sexually abstinent from five days prior to enrollment to seven days after their last dose of study film, and to use condoms during vaginal intercourse until three weeks after their final MB66 administration, and to continue their requirement for use of an effective contraceptive regimen (see Section 9.2). Urine pregnancy tests will be done on Screening, Enrollment and Exit Visits. Participants with positive pregnancy tests at Screening or Enrollment Visits will not be enrolled. Participants with a positive pregnancy test at the Exit Visit will be referred for obstetrical care. Women who become pregnant during the study period after randomization and exposure to study product will not be excluded from analysis. The study team will request that the participant signs a medical release form, so that information about the status of the pregnancy and health of the newborn can be included in future reports about the study product. Every effort will be made to maintain periodic contact with the participant after study termination until the completion of the pregnancy in order to determine the pregnancy outcome, and to obtain records from the facility at which the outcome occurred.

11.2.3 Interim (Ad Hoc) Visits

Ad hoc interim visits may be performed at any time during the study at the discretion of the investigator, for the following or other reasons:

- For administrative reasons, e.g., a participant may have questions for study staff, or may need to re-schedule a follow-up visit
- In response to AEs. When interim contacts or visits are completed in response to participant reports of AEs, study staff will assess the reported event clinically and provide or refer the participant to appropriate medical care. Moreover, all AEs ongoing at the end of study participation will be followed clinically until stabilized or resolved and referrals for additional care will be provided if appropriate.
- As an Early Termination Visit for participants who elect to discontinue participation in the study
- As a Pregnancy Visit, if a woman becomes aware of being pregnant
- For other reasons at participant request

Details of the interim visit will be recorded in the CRF.

11.2.4 Study Discontinuation Procedures

Participants in Segment B who discontinue study product will be encouraged to remain in the study for follow up safety evaluations.

11.3 Clinical Evaluations and Procedures

Physical exams will include the following assessments:

Vital signs:

- Temperature
- Blood pressure

- Pulse

Measurements of:

- Weight (screening only)
- Height (screening only)

Clinical assessments of:

- General appearance
- Cardiac exam (screening only)
- Respiratory exam (screening only)
- Abdomen (to include assessment of distension and tenderness) (All visits)
- Skin (screening only)

Pelvic exams will include the following assessments, in addition to other specific testing procedures as outlined in specific visit tables:

- Visual inspection of external genitalia
- Speculum exam
- Bimanual exam to assess internal organs (screening only)

Additional assessments may be performed at the discretion of the examining clinician in response to symptoms or illnesses present at the time of the exam.

11.4 ACCEPTABILITY ASSESSMENT (CASI)

At the end of the 7-day use period, participants will complete a computer assisted structured interview (CASI) questionnaire. This questionnaire will focus on capturing participants' preferences for the study product, and will capture conventional acceptability and willingness to use. The CASI survey will include scales developed under Dr. Morrow's MIP awards (R21/R33 MH80591 and AI076967. Items are divided into two sections: application/insertion experience and ambulation/daily activity experience. The survey will consist of both conventional acceptability items (capturing "face-value" characteristics, such as the participant's reaction to the products color and texture from a likes/dislikes perspective) and perceptibility (user sensory perception and experience) items. Items and responses are read to the participant via audio-recorded wave files linked to each item in the programming.

The User Perception scales administered to participants in this study consist of statements (i.e., items), written in a factual tone, to which participants respond with varying levels of agreement (i.e., 1=Do Not Agree at All; 2=Agree a Little; 3=Agree Somewhat; 4=Agree a Lot; 5=Agree Completely). Each item is designed to capture a specific construct, concept, or experience targeted in the study.

Each scale has a different number of items. As such, each scale has a different potential score range. In interpreting each scale score, averaged item means will be considered as an indication of the overall disagreement-agreement for each scale. Thus, the item mean represents the general level of agreement the sample endorsed with respect to a given film experience. Female vaginal

scales were psychometrically validated. The scales have not yet been fully validated for *film* evaluation.

11.5 Laboratory Evaluations

11.5.1 Listing of laboratory tests

Testing methods and testing laboratories are listed in 156 according to specimen type and are discussed further in Section 11.5.2.

Table 15: Laboratory Test Methods

Sample	Method	Laboratory
**Urine**	HCG kit test, dipstick urinalysis<br>NAATS for CT, GC, and trichomonas	Clinical staff testing<br><br>Miriam Hospital Laboratory CLIA #41DO701581
**Vaginal swabs and Tear Flo strips**	Swab for Gram stained vaginal smear	Miriam CFAR Laboratory (non-diagnostic laboratory)
	Swab stored frozen for later microbiome PCR	To be determined
	Vaginal pH, wet prep	Clinical staff testing
	Tear Flo strips for VRC01-N and HSV8-N ELISA	Intertek Pharmaceutical (non-diagnostic laboratory)
**Serum**	VRC01-N and HSV8-N ELISA assays	Intertek Pharmaceutical (non-diagnostic laboratory)
**Blood**	CBC, Liver and Renal Panels, and RPR	Miriam Hospital Laboratory CLIA #41DO701581
**Saliva**	Rapid HIV test	Clinical staff testing (Follow-up testing, when required is done in Miriam Hospital Laboratory, CLIA #41DO701581.)
**CVL**	Immune mediators	Anderson Laboratory, Boston University School of Medicine (non-diagnostic laboratory)
	Antiviral assays (HIV/HSV neutralization assays)	Anderson Laboratory, Boston University School of

Sample	Method	Laboratory
		Medicine (non-diagnostic laboratory)
**Endocervical/ Cervical cell collection**	Papanicolau smear (Visit 1) Pap smear	Miriam Hospital Laboratory CLIA # 41DO701581

11.5.2 Details of laboratory testing

Urine Samples

Study staff at the clinical site will use dipstick urinalysis to screen for possible urinary tract infection.. Urine will be tested for HCG via the QuPID One-Step Pregnancy Test. Urine will be tested by NAAT for CT, GC, and trichomonas (Gen Probe APTIMA).

Saliva Samples

Study staff at the clinical site will use the OraQuick ADVANCE Rapid HIV-1/2 Antibody Test for HIV antibodies (non-gp120 based, CLIA exempt). When required, follow-up testing on blood will be performed by the Miriam Hospital Laboratory as outlined in Appendix 2.

Serum Samples

Serum samples will be tested for VRC01-N and HSV8-N antibody levels, in validated assays using Intertek Pharmaceutical Services SOPs to GLP standards (Intertek Pharmaceutical Services 3985 Sorrento Valley Blvd., Suite C, San Diego, CA 92121).

Blood Samples

Screening and follow-up safety blood tests (CBC, Liver and Renal Panels, and RPR) will be done on blood samples by the Miriam Hospital Laboratory.

Vaginal Samples (Swabs and Tear Flo strips)

Swabs: Vaginal swabs will be used for sampling to make assessments of the cervicovaginal environment, including testing for vaginal pH, bacterial morphotypes assessed by Nugent Scoring of Gram stained vaginal smears, and stored frozen for microbiome analysis by polymerase chain reaction (PCR) for bacterial ribosomal DNA.

Tear Flo strips: Tear Flo strips (see rationale in Section 6.2.2) will be applied to the epithelium (one sample per specified location) until saturated to the predefined mark, and handled and analyzed per SOP. Collection will be done with care to collect an adequate but low-volume sample without disturbing the mucosa.

Cervicovaginal Lavage Samples:
CVL sampling will be used at 24 hours and 7 days, where it will not interfere with short time base (1 and 4 hour) sampling with Tear Flo strips. These CVL samples will be used for viral

neutralization studies and immune mediator testing where larger volume, moderately diluted samples are required. CVL sampling will be done through a speculum, with 5 mL of phosphate buffered saline dispensed three times through a syringe to wash the vaginal walls and ectocervix, then recollected from the pool accumulating in the posterior fossa.

Analysis of Antibody Content in CVL: Aliquots of lavage samples will be tested for VRC01-N and HSV8-N antibody levels (in validated assays using Intertek Pharmaceutical Services SOPs to GLP standards, Intertek Pharmaceutical Services 3985 Sorrento Valley Blvd., Suite C, San Diego, CA 92121).

Anti-viral Activity in CVL: As an exploratory endpoint to examine protection against infection, cervicovaginal lavage (CVL) fluid will be tested ex vivo for viral neutralization potency (against HIV-1 and HSV-2). (The low level of mucosal and systemic absorption of antibodies (molecular weight 150 kD) in primates, coupled with an extensive literature demonstrating that topically applied antibodies can protect against SHIV and HSV viral challenge (Sherwood *et al.*, 1996; Zeitlin *et al.*, 1997; Zeitlin *et al.*, 1998; Veazy *et al.*, 2003; Moog *et al.*, 2014) indicates that neutralizing MAbs bind to virus in the vaginal lumen. For this reason this exploratory efficacy endpoint is being done with CVL rather with tissue explant challenge models that have become common for antiviral small molecule drugs.) Aliquots of lavage samples will be tested to determine endpoint viral neutralization activity according to SOPs in the Anderson laboratory. HIV neutralization will be tested by TZM-bl HIV neutralization assay, using transmitter-founder (HIV23-17) and lab adapted (HIVBal) viruses. HSV neutralization will be tested by Vero cell plaque reduction assay, using Strain G HSV-2, and Strain KOS HSV-1.

Immune Modulators: Since HIV transmission is enhanced by genital tract inflammatory states (Mayer et al., 2011, Mauck et al., 2013), additional exploratory endpoint assays will be performed on CVL fluid to measure inflammatory cytokines (see below) by Luminex assay. Additionally, certain other immune mediators such as SLPI will be assayed (secretory leukocyte protease inhibitor) that appear to be protective against viral transmission (Mauck et al., 2013), and that may be suppressed by some prior candidate microbicides. Cytokines and other innate immune mediators will be handled and measured in the Anderson Laboratory according to procedures outlined in SOPs for this study. Cytokines to be measured via the Luminex 100TM Instrument (Luminex Co., Austin, TX) may include: IL-1beta, IL-6, TNF-alpha, MIP-1alpha, INF-gamma, GM-CSF, Il-12p40, C3b, RANTES, IP-10, Innate immune mediators including secretory leukocyte protease inhibitor (SLPI), lactoferrin, human β defensins 1-3, lysozyme, LL-37, and PMN elastase will be measured by commercial ELISA.

Vaginal microbiome indicators

- Gram stain of vaginal smears and Nugent scoring
- Vaginal microbiome assessment by bacterial ribosomal DNA polymerase chain reaction (BD eSwab stored frozen for later analysis at a TBN laboratory)

11.5.3 Specimen Preparation, Handling and Shipping

The site will adhere to the standards of good clinical laboratory practice and site standard operating procedures for proper collection, processing, labeling, handling, transport, and storage of specimens, in a manner consistent with institutional and OSHA guidelines. In cases where laboratory results are not available due to administrative or laboratory error, sites are permitted to make a single re-draw and/or re-collect specimens before counting as a missing test.

Specimens will be batch shipped at study completion from Miriam Hospital to collaborating outside laboratories on dry ice using express carriers (Federal Express or equivalent) according to a sample shipping SOP. Addresses of outside laboratoris are listed in Section 3.

11.5.4 Biohazard Containment

Appropriate blood, secretion, and respiratory precautions will be employed by all personnel in the collection of clinical samples and the shipping and handling of all clinical samples and isolates for this study, as currently recommended by the Centers for Disease Control and Prevention in the United States, the WHO internationally and the National Institutes of Health.

11.5.5 Total Blood Volume

Total blood volume drawn will be appoximately 100 mL for each participant in Segment A and approximately 180 mL for each participant in Segment B.

11.6 Quality Control and Quality Assurance Procedures

The Miriam Hospital Laboratory is CLIA-inspected and maintains a CLIA license. Because all of the proposed studies to be conducted in this project will be using an investigational drug, the studies will be conducted under IND and additional measures will be undertaken to assure that all protocols will be conducted under acceptable and established standard laboratory procedures.

12. ASSESSMENT OF SAFETY

12.1 Adverse Event Procedures and Reporting Requirements

12.1.1 Adverse Events Definitions and Documentation

An AE is defined as any untoward medical occurrence in a clinical research participant administered an investigational product and which does not necessarily have a causal relationship with the investigational product. As such, an AE can be an unfavorable or unintended sign (including an abnormal laboratory finding, for example), symptom or disease temporally associated with the use of an investigational product, whether or not considered related to the product. This definition is applied to all groups beginning from the time of first product use. The term “investigational product” for this study refers to both the MB66 film and placebo film.

Study participants will be provided instructions for contacting the study site to report any untoward medical occurrences they may experience, except for possible life-threatening events, for which they are instructed to seek immediate emergency care. Where feasible and medically appropriate, participants will be encouraged to seek evaluation at Miriam Hospital, where the study clinicians are based, and to request that a study clinician be contacted upon their arrival. With appropriate permission of the participant, whenever possible, records from all non-study medical providers related to untoward medical occurrences will be obtained for review. All participants reporting an untoward medical occurrence will be followed clinically until the occurrence resolves (returns to baseline) or stabilizes over a four-week period.

Study site staff will document in source documents all AEs reported by or observed in enrolled study participants regardless of severity and presumed relationship to study product.
For each study participant, AE documentation and reporting will be undertaken throughout the scheduled duration of follow-up.

Complete descriptions of all AEs will be entered in source documents, and will include:

1. AE term (i.e., the one term that best describes what occurred)
2. AE start and stop dates
3. Severity grade of the AE (see grading table information below)
4. Study product(s) and/or intervention(s) administered
5. Relationship of the AE to the study product(s) and/or intervention(s)
6. Action taken regarding the study product(s) and/or intervention(s)
7. AE outcome
8. What seriousness criteria, if any, were met

The PI/designee will grade the severity of each AE and the relationship of the AE to study product:

- AE severity will be graded per the Division of AIDS (DAIDS) Table for Grading the Severity of Adult and Pediatric Adverse Events, Version 2.0. [November 2014], and the Female Genital Grading Table for Use in Microbicide Studies (Addendum 1 to the DAIDS Table for Grading Adult and Pediatric Adverse Events, Version 1.0, November 2007 [Clarification dated August 2009]), except that asymptomatic BV will not be considered an AE. AEs not included in the Female Genital Grading Table for Use in Microbicide Studies mentioned above will be graded by the Division of AIDS (DAIDS) Table for Grading the Severity of Adult and Pediatric Adverse Events, Version 2.0. [November 2014]. In cases where a genital AE is covered in both tables, the Female Genital Grading Table for Use in Microbicide Studies will be the grading scale utilized.
- The relationship of all AEs reported on CRFs will be assessed based on the Manual for Expedited Reporting of Adverse Events to DAIDS, and the clinical judgment of the PI/designee. The study products that must be considered when AE relationships are assigned are MB66 film and placebo film.

The DAIDS Table for Grading Adult and Pediatric Adverse Events, the Female Genital Grading Table for Use in Microbicide Studies, and Version 2.0 of the Manual for Expedited Reporting of Adverse Events to DAIDS are available on the DAIDS Regulatory Support Center (RSC) web site: http://rsc.tech-res.com/.

All AEs will be captured on an AE log form. The form will be reviewed at each study visit and updated as needed. For any serious or expedited AEs (SAEs/EAEs) that are continuing at a participant's study exit visit, the PI/designee must establish a clinically appropriate follow-up plan for the AE and review with the DAIDS Medical Officers. At a minimum, the AE must be re-assessed by study staff at least 2 weeks after the participant's study exit visit; additional evaluations also may take place at the discretion of the PI/designee. The same approach must be taken for any AEs that are found to have increased in severity at the study exit visit. For those AEs requiring re-assessment, if the AE has not resolved or stabilized at the time of re-assessment, study staff will continue to re-assess the participant at least once per month while the study is ongoing. After the study has ended, all AEs requiring re-assessment will be re-assessed at least once within the 30-60 days after the study end date, and referrals for additional care will be provided if appropriate.

12.1.2 Expedited Adverse Event Reporting

Expedited Adverse Event Reporting to DAIDS

Requirements, definitions and methods for expedited reporting of Adverse Events (AEs) are outlined in Version 2.0 of the DAIDS EAE Manual, dated January 2010, which is available on the RSC website (http://rsc.tech-res.com/safetyandpharmacovigilance/). The DAIDS Adverse Experience Reporting System (DAERS), an internet-based reporting system, must be used for expedited AE reporting to DAIDS. In the event of system outages or technical difficulties, expedited AEs may be submitted via the DAIDS EAE Form.

The current DAIDS EAE Manual (Version 2.0), which is also available on the RSC website at: http://rsc.tech-res.com/safetyandpharmacovigilance/. The DAIDS Adverse Experience Reporting System (DAERS), an internet-based reporting system, will be used for expedited AE reporting to DAIDS. In the event of system outages or technical difficulties, expedited AEs will be submitted via the DAIDS EAE Form. This form is available on the RSC website: http://rsc.tech-res.com/safetyandpharmacovigilance/.

Reporting Requirements for this Study

The SAE Reporting Category, as defined in Version 2.0 of the DAIDS EAE Manual, will be used for this study. The study agents that must be considered in determining relationships of AEs requiring expedited reporting to DAIDS are: MB66 film, and placebo film.

Reporting Period

AEs must be reported on an expedited basis during the entire study duration for an individual subject (from study enrollment until study completion or discontinuation of the subject from

study participation for any reason). After the protocol-defined AE reporting period, unless otherwise noted, only Suspected, Unexpected Serious Adverse Reactions (SUSARs) as defined in Version 2.0 of the EAE Manual will be reported to DAIDS if the study staff become aware of the events on a passive basis (from publicly available information).

13. CLINICAL MANAGEMENT

13.1 Clinical Management of Adverse Events

By definition, an adverse event can be either a new finding or symptom or a worsening of a pre-existing condition. In order to accurately capture adverse events in follow-up, a thorough baseline history will be obtained at Visit 1 and Visit 2. For example, for participants who endorse a history of headache, site staff will probe for and record details surrounding the condition such as frequency, location, duration, medication use, triggers, etc. Only by eliciting a full description will study staff be equipped to determine whether a subsequent event in follow-up is a clinically distinct event. Adverse events will be elicited during the safety contact telephone call and in-clinic follow-up visits. Referral to appropriate care will be offered to participants as needed. Vaginally applied medications as management for AEs should be avoided if possible (instead using systemic medication for treatment where appropriate). Criteria for product hold for AEs thought related to the study film, and for discontinuation of an individual's participation in the study are described below.

13.2 Pregnancy

Pregnancy during study participation is unlikely due to the requirement for effective contraception. However, urine pregnancy tests will be performed during the investigational protocol. Participants who become pregnant in follow-up will be referred for obstetric care. The participant will be permanently discontinued and the following procedures will be performed at discontinuation: complete metabolic panel and complete blood count. Pelvic exams will only be performed to evaluate a participant's reported symptom. No genital specimens will be collected in a pregnant participant. Staff will continue follow-up contact with the participant to obtain pregnancy outcome data. Of note, the participant will be encouraged to continue in the study so that safety data might be collected. See further detail in Section 11.2.3.

13.3 Acquisition of HIV Infection on study

See Section 11.2.2.

13.4 Unexpected Menstrual Like Bleeding

In instances when unexpected menstrual like bleeding occurs, participants will be instructed to come to the study site for an evaluation. This unexpected bleeding will be considered an adverse event and appropriate clinical management will be based on exam findings.

Because every attempt will be made to enroll a participant into the study during the first two weeks of her menstrual cycle (Segment A) or first week of her menstrual cycle (Segment B), it is unlikely that a participant will menstruate during the period of product use. In the setting of menses or menses like bleeding that occurs during the week of product use, product use will be held and the PSRT consulted.

13.5 Criteria for Discontinuation

Participants may voluntarily withdraw from the study for any reason at any time. The Site PI/designee also may withdraw participants from the study to protect their safety and/or if they are unwilling or unable to comply with required study procedures. Participants also may be withdrawn if the study sponsors, government or regulatory authorities, including the Office of Human Research Protections (OHRP), or site IRBs/ECs terminate the study prior to its planned end date. Every reasonable effort will be made to complete a final evaluation of participants who withdraw or are withdrawn from the study prior to completing the follow-up Visit. Study staff members will record the reason(s) for all withdrawals in participants' study records.

13.6 Product Hold

Because of the short duration of both Segments of the study (single dose in Segment A, 7 daily doses in Segment B), product holds are not anticipated to be useful. Instead, participants with Grade 1 or 2 adverse events will be allowed to complete the exposure period for Segment B. For Grade 3 or 4 AEs, rather than product hold, product use will be discontinued permanently. In the unlikely event that a participant is intolerant of the study product immediately after placement, the site clinician will perform a pelvic exam and cervicovaginal lavage to remove all visible product.

13.7 Criteria for Discontinuation

13.7.1 Permanent Intervention Discontinuation,

The criteria for permanent discontinuation of further study product(s)/ intervention(s) for an individual participant are:

a) Grade 3 or 4 Adverse Event (per Section 13.6

b) Requirement for prohibited concomitant medications

c) Pregnancy

d) Completion of study product course as defined in the protocol

e) Request by participant to terminate study product

f) Clinical conditions, which in the best judgment of the investigator, are believed to be harmful or potentially life-threatening to the participant, even if not addressed in the AE Management section of the protocol

g) Recommended by the EC/IRB, PSRT, DAIDS Medical Officer, OHRP, or FDA

The participant will continue to be followed with her permission if study product is discontinued. No subsequent modifications to the visit schedule and duration of continued follow-up will be made, except for discontinuation of the study product.

13.7.2 Permanent Study Discontinuation,

The criteria for premature discontinuation from the study for an individual participant are:

a) Lost to follow up as evidenced by failure by the participant to attend 2 consecutive clinic visits, at the discretion of the site investigator

b) Participant repeatedly non-compliant (has not inserted any home doses of film by day 4 when contacted by telephone safety call)

c) Pregnancy or breastfeeding (if applicable)

d) Request by participant to withdraw

e) Has any other condition that, in the opinion of the Investigator or designee, would preclude informed consent, make study participation unsafe, complicate interpretation of study outcome data, or otherwise interfere with achieving study objectives.

13.8 SAFETY MONITORING

The study site investigators are responsible for continuous close safety monitoring of all study participants, and for alerting the Protocol Team and IRB if unexpected concerns arise. A sub-group of the Protocol Team, including the Protocol Chair or designee, the DAIDS Medical Officer, and an IPCP Co-PI will serve as the Protocol Safety Review Team (PSRT). If necessary, external experts representing expertise in the fields of microbicides, biostatistics, HIV transmission, and medical ethics may be invited to review the events. The PSRT and the study site will cooperate closely to monitor participant safety and respond to occurrences of toxicity in a timely manner. Study staff will seek to maintain close and effective communication with study participants and communication with and cooperation among study staff, investigators, the PSRT, and the DAIDS Medical Officer. The following findings will serve as the minimum criteria to pause or stop enrollment in Segment A, prevent the initiation of Segment B, and pause or stop enrollment in Segment B. However DAIDS and/or the PSRT can stop the study at any time for any concerning findings of lesser severity than those listed here:

1. If any participant manifests a grade 3 or higher AE that is judged to be related to study product

2. If any participant develops deep epithelial disruption or ulceration of the genital epithelium.

3. If any participant develops erythema or edema of more than 50% of the combined vaginal and cervical surface (grade 2) or more than 50% of the vulvar surface (grade 2).

If enrollment is paused, its reinstitution will require the unanimous agreement of all members of the PSRT.

13.9 CLINICAL DATA SAFETY REVIEW

The Data Management Team will generate data summaries on a monthly basis for the PSRT. These data summaries will include adverse event, accrual and retention data. The Protocol Chair (or designee) will evaluate adverse event data independently and present those to the PSRT on the monthly calls, and present adverse event data to the PSRT via telephone for review. If more urgent safety matters arise, these calls can occur more frequently. The PSRT will determine whether or not the study protocol should continue as originally designed, should be changed, or should be terminated.

The IRB will be notified of any serious and unexpected adverse events according to the policies outlined in the Miriam Hospital IRB Policy and Manual of Operations.

The following information will be submitted to the Miriam Hospital, and Boston University Medical Center IRB at the time of renewal of a research protocol, as required by IRB guidelines:

- The frequency of monitoring during the renewal interval, including the dates of data and safety monitoring;
- A summary of any assessment performed to evaluate external factors or other relevant information that may have an impact on the safety of study participants or the ethics of the research study;
- A summary of the outcome of procedural reviews conducted to ensure subject privacy and research data confidentiality;
- Any conclusions regarding changes to the anticipated benefit-to-risk ratio of study participation and final recommendations related to continuing, changing, or terminating the study, with accompanying rationales as appropriate.

14. STATISTICAL METHODS

14.1 REVIEW OF STUDY DESIGN

The primary aim of this study is to assess the local and systemic safety of MB66, a vaginal anti-microbial consisting of two monoclonal antibodies (MAbs) formulated in a polyvinyl alcohol film. One of the MAbs in MB66 neutralizes herpes simplex virus type 2 (HSV-2), and the other neutralizes human immunodeficiency virus, type 1 (HIV-1). The first segment of the study is a single dose study of MB66 film administered to the vagina in adult healthy female study participants. The second segment of the study is a repeated dose study with seven daily vaginal administrations of MB66 film or placebo film in adult healthy female participants.

Screening for sexually transmitted infections (HIV, *C. trachomatis, N. gonorrhoeae, T. vaginalis, syphilis, mucopurulent cervicitis and active genital ulcers*), pregnancy, and a routine pelvic exam will be conducted at Visit 1, and urogenital, systemic and menstrual symptoms will be collected via symptom review with participants to exclude study candidates with pre-existent

reproductive tract inflammation and epithelial disruption. This testing will be repeated during subsequent examinations if clinically indicated. Data on epithelial inflammation and disruption will be collected during visual examination of the reproductive tract. Pharmacokinetic measures will assess the rate of film dissolution and the local concentrations of and systemic absorption of MB66 MAbs following a single and a seven-day exposure. Assessments of immune mediator concentration and various measures of vaginal ecology will be made before and after MB66 film use.

14.2 Sample Size and Accrual

The primary aim of the study is to assess the local and systemic safety of one and seven day exposures to MB66. The primary endpoint is the proportion of participants who experience a Grade 2 or higher adverse event (AE) deemed related to study product. (The proposed total sample size is N=38 evaluable participants divided into Segment A (with n = 8 evaluable), and Segment B (with two arms, a MB66 arm and a placebo film arm, each with n = 15 evaluable). Evaluable participants (evaluable for the primary endpoint) in Segment A will be defined as participants who complete the Screening and Enrollment Visits and return for Visit 3. Evaluable participants in Segment B will be defined as participants who complete the Screening and Enrollment Visits and return for Visit 4 and use study product for at least 5 of the previous 7 days including the day before presentation (as assessed with MEMS cap data). Non-evaluable participants will be replaced, however, their data relevant to endpoints other than the primary endpoint will be preserved and used in tabulation and analysis of secondary and exploratory endpoints. In addition, if a participant becomes non-evaluable prior to the predefined visits described above, but experiences an AE during the time they were on study, their data will be included in the primary analyses as well. Based on previous studies of vaginal products conducted at the study site, the accrual of 38 evaluable subjects with normal reproductive tracts is expected to require the screening of approximately 70 participants and the enrollment of approximately 43 participants. The target for retention will be approximately 90% of enrolled participants over the 7-14-day follow-up period. Therefore, it is anticipated that 23 women exposed to active study drug in all arms (8 during Segment A and 15 during Segment B) will be evaluable. Table 16 calculates the probability of events in Segment A participants (n=8) and Table 17 calculates the probability of events in Segment B participants (n=15).

Table 16: Exact Binomial probabilities for different scenarios for Segment A (n=8) for Study MB66-01

Event Rate	P (No events | n=8)	P (1 event | n=8)	P (2 or more events | n=8)
1%	0.92	0.07	0.000054
5%	0.66	0.28	0.057
10%	0.43	0.38	0.19
15%	0.27	0.38	0.34
20%	0.17	0.34	0.50
25%	0.10	0.27	0.63
30%	0.058	0.20	0.74

35%	0.032	0.14	0.83
40%	0.017	0.09	0.89
45%	0.0084	0.055	0.94
50%	0.0039	0.031	0.96

Table 17: Exact Binomial probabilities for different scenarios for Segment B (n=15) for_Study MB66-01

Event Rate	P (No events | n=15)	P (1 event | n=15)	P (2 or more events | n=15)
1%	0.86	0.13	0.01
5%	0.46	0.37	0.17
10%	0.21	0.34	0.45
15%	0.09	0.23	0.62
20%	0.04	0.13	0.83
25%	0.0134	0.067	0.92
30%	0.005	0.03	0.965

The sample size for Segment A is large enough to rule out 50% Grade 2 or higher adverse rate with n=8 patients. With 8 participants in this segment a one-sided 95% Clopper-Pearson (exact) binomial confidence interval will have an upper limit of 47% if one out of eight women has a Grade 2 (or higher) adverse event (AE2) related to the study product. Furthermore, the sample size for Segment B was chosen to have an adequate sample size to rule out 30% grade 2 or higher adverse event rate for each of the groups. With 15 participants in an arm a one-sided 95% Clopper-Pearson (exact) binomial confidence interval will have an upper limit of 27.9% if one out of 15 women has an AE2 related to the study product.

In addition to estimating the rate of Grade 2 (or higher) AE's in Segment B alone, estimates of combined AE2 rates can be derived by pooling the participants from Segment A and Segment B. Segment A participants (n=8) can be pooled with the participants from Segment B, allowing for AE2 estimates to be made using n=23 participants. One-sided 95% Clopper-Pearson (exact) binomial confidence intervals will be estimated for these AE2 rates. For the analyses with n=23 participants, the confidence intervals will have an upper bound of 29.4% if 2 (or fewer) participants have an AE2. These analyses will provide supportive data to the primary endpoint analysis that is based on the Segment B (n=15) participants only.

14.3 JUSTIFICATION FOR PLACEBO

This study will utilize a placebo film in Segment B. The placebo control will be a vehicle film containing the same excipients as the active antibody film, and will be administered as one full film in Segment B. Inclusion of a placebo film in this trial will allow investigators to establish the prevalence of adverse events that may be associated with exposure to the film vehicle excipients versus the effect of the MAbs comprising MB66.

The inclusion of a Placebo Group in Segment B also will allow us to rule out large differences in AE2 rates between the Active and Placebo groups. Table 18 below depicts detectable differences and statistical power when comparing Segment B Placebo and Active arms (n=15 per group) for AE2 rates using a Fisher's exact test with alpha=0.05 (2-sided) for comparisons.

Table 18: Detectable differences and statistical power

AE2 Rate in Active Arm	AE2 Rate in Placebo Arm	Power
0.001	0.46	88%
0.067	0.60	83%
0.13	0.73	88%
0.2	0.8	87%

So for instance, if 1/15 (6.7%) of participants in the Active Arm has an AE2 whereas 9/15 (60%) or more have an AE2 in the Placebo Arm there will be 83% power to detect this difference. It should be noted that this calculation also suggests that if 6.7% of participants in the Placebo Arm had an AE2 and 60% (or more) participants in the Active Arm had an AE2 there would be 83% power to detect that difference as well.

Conversely, our rationale for *not* using a placebo in Segment A (single dose, open label, active film only) is that this preliminary Segment is very small, and is included only to provide a very limited exposure with this first in human use of the MB66 film before going on to the larger Segment B, with a repeated dosing regimen. We acknowledge that there is a risk that without a concurrent vehicle control placebo arm in Segment A, AEs that are due to the film vehicle rather than the mAbs. However, we judge this risk to be very low based on the widespread use of a commercial vaginal film (VCF, Vaginal Contraceptive Film) a PVA-based film with very similar excipients, and the low toxicity of the MB66 vehicle placebo in preclinical animal studies (see Section 6.1.3). For this reason, we believe more will be gained by increasing the number of participants in Segment A receiving active MB66 film than from including a vehicle control placebo arm.

14.4 STUDY ENDPOINTS

14.4.1 Primary Endpoint: Grade 2 Adverse events

The primary safety endpoint is the clinical or laboratory evidence of a Grade 2 or higher Adverse Event that is judged to be related to study product. Adverse events will be graded according to the following reference: *Division of AIDS (DAIDS) Table for Grading the Severity of Adult and Pediatric Adverse Events, Version 2.0, Nov 2014*, and the Female Genital Grading Table for Use in Microbicide Studies (Addendum 1 to the DAIDS Table for Grading Adult and Pediatric Adverse Events, Version 1.0, November 2007 [Clarification dated August 2009]).

14.4.2 Secondary Endpoints: Pharmacokinetics (PK)

Distribution and Persistence of MB66 MAbs in CVF

The vaginal distribution and persistence of MB66 MAbs, VRC01-N and HSV8-N, will be determined by ELISAs carried out on vaginal fluid collected via Tear Flo strips. The fluid will be collected from different regions of the vagina and at different times after administration.

Systemic Absorption of VRC01 and HSV8

Serum samples will be obtained for testing of systemic absorption by ELISA for the VRC01-N Drug Substances. The expected ~50% probability of seropositivity to HSV-1 and/or HSV-2 precludes unambiguous assays for the absorption of the anti herpes Mab (HSV8-N) in participants with preexisting seropositivity, though informative data will be available from HSV seronegatives.

Dissolution of Study Film

Visual assessments of the degree of dissolution of study film will be made during speculum exams at intervals after vaginal insertion. The degree of film solubilization (conversion from a solid film to a hydrated gel-like consistency) will be assessed as follows: 100%, between 75 and 100%; between 50 and 75%; between 25 and 50%; between none and 25%; and no evident dissolution.

14.4.3 Exploratory Endpoints

Ex vivo antiviral effect

Assays will be done to determine the ex vivo viral neutralization of HIV-1 and HSV-2 in cervicovaginal lavage fluid after MB66 dosing. The endpoint of viral neutralization activity will be determined with ex vivo challenge with HIV-1 and HSV-2 addition to serially diluted CVL fluid. *In vitro* anti-HIV activity will be determined by the TZM-bl assay, and the *in vitro* anti-HSV-2 activity will be assayed by ELVIS HSV Test System, both in Dr. Anderson’s laboratory at Boston University School of Medicine.

Cervicovaginal microbial environment

Vaginal pH

Vaginal pH will be measured with pH paper. A change from normal (< 4.5) at baseline to $\geq$ 4.5 at Visit 4 will be considered a clinically significant change in vaginal pH.

Nugent score

The morphotypes of vaginal bacteria will be assessed using Nugent scoring of Gram stained vaginal smears. The Nugent score is graded 1 to 10 as follows:

1) Normal, 0-3
2) Intermediate, 4-6
3) Bacterial Vaginosis, 7-10

Vaginal microbiome assessment

Becton Dickenson eSwabs will be stored frozen for characterization of the vaginal microbiome before and after study film use by polymerase chain reaction (PCR) to determine the relative proportions of resident vaginal bacteria. Meaningful changes will be defined as a shift from a lactobacillus-dominated community to a mixed community dominated by BV associated bacteria.

Levels of Immune mediators in Cervicovaginal Lavage

Concentrations of cytokines and other immune mediators will be measured before and after study film exposure.

Acceptability

The acceptability of MB66 film will be assessed by computer administered structured interview (CASI) after 7 days of MB66 or placebo film.

14.5 Blinding

Segment A will be open label since all participants will receive active film. Segment B participants will be blinded to assignment of active drug versus placebo. Because the active and placebo films are not identical in appearance, study staff cannot reliably be blinded to assignment, and hence we characterize Segment B as “single blind”. However, we will attempt to maintain blinding to assignment during laboratory testing, and among data recording and data analysis teams.

14.6 Random Assignment

The randomization scheme will be generated and maintained by Dr. Politch at Boston University. Women will be randomized to one of the two arms of Segment B in a 1:1 ratio using block randomization (5 blocks of 6 women). Since some participants may become non-evaluable prior to the adequate outcome or safety assessments (see Section 14.2) these participants will need to be replaced in the study. This could potentially lead to an imbalance in the treatment allocation. To handle this situation, after each block of women is randomized, it

will be noted when any are removed from the study because of being non-evaluable. If equal numbers of placebo and active drug recipients are being removed then the randomization plan as described above will retain balance in group allocation, and an additional block of women (with size equal to the total number of non-evaluable women) will be randomized. However, if an unequal number of participants from one group are removed because they become non-evaluable (e.g., 2 women in the placebo group vs. 1 woman in the active film group), then one of the remaining blocks of non-randomized women will be altered to include additional participants from the group had more participants removed (i.e., the last block will be constructed to have 9 women (rather than 6) with 5 to be randomized to the film only group and 4 to be randomized to the film plus drug group in this example).

14.7 DATA MONITORING AND ANALYSIS

14.7.1 Data Monitoring

This clinical trial will be conducted in compliance with the protocol, GCP guidelines, and applicable regulatory requirements. All research charts are maintained in locked files in a locked room.

Some case report forms will be used as source documents, first point of data entry for this protocol. The case report forms will be photocopied and mailed to the data management group after all identifying information other than the participant ID number has been removed. Study data management staff will manually review the forms for completeness and accuracy. If there are any responses that are incomplete or unclear, the staff person will speak or email with the clinician in question as soon as possible to resolve the problem. If necessary, the study staff member who collected the data will mark out the previous response (not erase or obliterate it), fill in the new response, and date and initial the change. Once the source documents are ready for computer entry, data management staff will enter data into a data entry system.

14.7.2 Primary Endpoint Analysis

The primary endpoint of the study is to determine the incidences of Grade 2 or higher Adverse Events deemed related to study product. Adverse Events will be measured using the definitions provided in the Division of AIDS (DAIDS) Table for Grading the Severity of Adult and Pediatric Adverse Events, Version 2.0. [November 2014], and the Female Genital Grading Table for Use in Microbicide Studies (Addendum 1 to the DAIDS Table for Grading Adult and Pediatric Adverse Events, Version 1.0, November 2007 [Clarification dated August 2009]). For Segment A, the primary analysis will be to determine whether any Grade 2 or higher Adverse events deemed related to study product occur. If Grade 2 or higher AEs do occur, the PSRT will determine whether advancement from the first to second group of Segment A participants should be allowed, and whether advancement from Segment A to Segment B should be allowed. This determination will be made based on the number, type, and product relatedness of the AEs observed.

For Segment B, the primary analysis of the primary endpoint will be conducted as follows. First, the incidence rate and one-sided 95% Clopper-Pearson (exact) binomial confidence intervals of

AE2's will be estimated for each group. The focus of these analyses will be to determine whether a 30% AE2 rate can be ruled out based on the data collected in the study. This will be shown if the upper bound of the 1-sided 95% Clopper Pearson (exact) binomial confidence interval is less than 30% which will occur if zero or one participant out of the 15 enrolled (in each arm) have an AE2. In addition, an exploratory comparison between the two groups in Segment B (active and vehicle) will be performed using a Fisher's Exact test to determine whether the number of participants with Grade 2 or higher AEs are different between groups. In addition to estimating the rate of Grade 2 (or higher) AE's in Segment B alone, estimates of combined Grade 2 or higher AEs rates can be derived by pooling the participants from Segment A and Segment B. In addition, Segment A participants (n=8) can be pooled with the participants from Segment B, allowing for Grade 2 or higher AEs estimates to be made using n=23 participants. One-sided 95% Clopper-Pearson (exact) binomial confidence intervals will be estimated for these AE2 rates. For the analyses with n=23 participants, the confidence intervals will have an upper bound of 29.4% if 2 (or fewer) participants have an Grade 2 or higher AEs. These analyses will provide supportive data to the primary endpoint analysis that is based on the Segment B (n=15) participants only. Additional detail of the statistical analyses will be provided in a separate statistical analysis plan (SAP), which will be prepared and signed off prior to completion of the study.

14.7.3 Secondary Endpoint Analysis

Summary statistics of frequencies, percentages, and 95% confidence intervals will be provided for categorical data. Means, standard deviations, medians, ranges and 95% confidence intervals will be provided for continuous data. Methods for the analyses of the secondary endpoints are described below. This Pre-Phase I study may not have an adequate sample size to detect significant differences within or between treatment arms.

In general, for continuous measures that are collected at multiple time points (vaginal and systemic antibody levels), repeated measures mixed models will be fit that allow the individual women to be considered as random effects in the models. Whenever possible the actual time of the measurement will be recorded during the study and the actual time interval between measures (i.e. number of hours between measures) can be considered in the repeated measures mixed models for accounting for time. This will allow for potentially a more sensitive analysis to the impact of time between measures to take place since there may be some variability among participants in the timing of the assessments. These models will be exploratory, but will allow longitudinal comparisons to be made both within treatment group and between treatment groups. For categorical or binary outcomes measured at multiple time points (film dissolution) a generalized estimating equations (GEE) approach will be used to compare participants within and between groups. As stated above, these analyses will be considered as primarily exploratory since there will be a limited sample size. If measures are taken only at one post randomization time point then we can compare groups using 2-sample t-tests (for continuous variables) and Fisher's exact tests (for categorical variables).

If the continuous data collected does not follow a normal distribution then appropriate transformations (i.e. log transformation) will be considered and if these do not fix the problem then non-parametric approaches will be considered (i.e. Kruskal Wallis tests).

14.7.4 Exploratory Endpoint Analysis

Introduction

Given the small sample size (n~30), and the preclinical nature of this research, comparisons between the active and placebo products are not the primary aim of this study. However, we will compute the effect sizes for all of the comparisons between formulations to provide guidance for outcomes interpretation and for future studies. While we do not expect many comparisons between active and placebo products to be statistically significant in this study, we do anticipate that some comparisons may be statistically significant, and in either case provide useful data for planning follow-up efficacy studies.

Ex vivo antiviral effect

One-way analysis of variance and Kruskal Wallis tests will be used, where appropriate, to evaluate differences in the antiviral activity in CVL between MB66 and placebo arm. In addition, analysis of covariance (ANCOVA) models will be considered when comparing groups. These models would include the baseline HSV seropositivity as a covariate in the models since there may be an imbalance between groups on this pre-treatment characteristic.

Cervicovaginal microbial environment

Vaginal pH: Vaginal pH levels will be compared between groups using two-sample t-tests. Pre- to post-exposure shifts will be analyzed with a longitudinal mixed model.

Nugent score: Differences in the categorized Nugent score at each visit and between the MB66 and placebo arms will be evaluated using chi-square tests, while McNemar's tests will be used to evaluate pre- to post-exposure shifts between normal (score 0-3) and abnormal (score 4-10) vaginal microflora within each treatment arm and from baseline (Visit 2, pre-exposure) to Visit 4 (one week post initiation of dosing). Any shift from normal at baseline to intermediate or Bacterial Vaginosis at Visit 4 will be considered a meaningful change in vaginal flora.

Vaginal microbiome assessment: Samples will be stored frozen for later analysis for the prevalence of microorganisms determined by polymerase chain reaction (PCR) for bacterial ribosomal DNA. The prevalent microorganisms will be compared between the MB66 and placebo arms using Fisher's exact tests, while pre- to post-exposure shifts will be evaluated within each group using McNemar's tests. Prevalence of microorganisms determined by polymerase chain reaction (PCR) for bacterial ribosomal DNA (as the percent of total identified organisms) will be compared between the MB66 and placebo arms using 2 sample t-test if distribution is normal (and after log transformation if not normal). Pre- to post-exposure shifts will be analyzed with a longitudinal mixed model (log transformed if not normally distributed).

Levels of Immune mediators in Cervicovaginal Lavage

These measures will be compared between groups using two-sample t-tests. Based on previous experience with these outcomes, it is anticipated that log-transformations of the outcome measures will need to be performed prior to performing group comparisons to control for the skewed nature of the data. Pre- to post-exposure shifts will be analyzed after log transformation with a longitudinal mixed model. In addition to these models, adjusted models (i.e. ANCOVA instead of two-sample t-tests) will be considered when comparing groups. These models would include the baseline HSV seropositivity as a covariate in the models.

Acceptability via CASI Interview

Groups will be compared using two-sample t-tests if the outcome data follows a normal distribution. Despite the relatively small sample size (n=30 total, 15 per group) based on our past research into scale score comparisons conducted on a number of different products, we anticipate that meaningful differences in some scales may be detected in this study. For example, with respect to daily use activity scales in our previous study among a sample of 24 heterosexual couples, effect sizes resulting in significant differences in pair-wise comparisons of 3 different formulation conditions ranged from 0.60 (considered a medium effect size) to 2.67 (considered a very large effect size).

14.7.5 Analysis Cohort

The cohort will be defined as participants who are enrolled. It will be used for analyses of baseline characteristics, protocol deviations and violations, and trial conduct. The intent-to-treat cohort (ITT) will be defined as those who were enrolled, regardless of how many doses of drug were administered. Since the primary focus for this study is to estimate accurately the true rate of AE2 events, a modified ITT (mITT) group will be used for the primary analyses. This mITT group is defined as evaluable participants for the primary endpoint are described in Section 14.2 for both Segment A and Segment B. The per protocol (PP) cohort is a subset of the evaluable cohort with subjects who meet all protocol specifications.

15. DATA HANDLING AND RECORD KEEPING

15.1 DATA QUALITY ASSURANCE

Site visits will be conducted to verify the qualifications of each Investigator, inspect the study center facilities, and inform the Investigators of their responsibilities and the procedures for ensuring adequate and correct documentation.

Investigator training will be held to introduce Investigators and their study staff to the clinical protocol, CRFs, study procedures, regulatory requirements, and use of the study assessments.

The Investigator is required to prepare and maintain adequate and accurate case histories designed to record all observations and other data pertinent to the study for each study participant. All information recorded on the CRFs for this study must be consistent with the participant's source documentation (i.e., medical records).

15.2 Direct Access to Source Data and Study Audits

Site monitors will perform onsite visits to review protocol compliance, compare CRFs and individual participant's source records, assess drug accountability, check for CRF completeness and clarity, and ensure that the study is being conducted according to pertinent regulatory requirements. CRF entries will be verified with source documentation. The review of medical records will be performed in a manner to ensure that participant confidentiality is maintained.

Representatives of the IRB, FDA, DAIDS, and/or the Sponsor may also wish to carry out such data checks during onsite audit inspections, including direct access to source data.

15.3 Archiving Study Records

Study documentation must be retained for a minimum of 2 years after the last approval of a marketing application and until there are no pending or contemplated marketing applications or until at least 2 years have elapsed since the formal discontinuation of clinical development of the investigational product. However, these documents may be retained for a longer period if required by the applicable legal requirements.

The investigator will maintain, and store securely, complete, accurate and current study records throughout the study. Study records will not be destroyed prior to receiving approval for record destruction from DAIDS. Applicable records include source documents, site registration documents and reports, correspondence, informed consent forms, and notations of all contacts with the participant.

16. CLINICAL SITE MONITORING

Study monitoring will be carried out by Pharmaceutical Product Development Inc., which will perform this function under contract to DAIDS (PPD, Wilmington, NC). Site monitoring visits will be conducted to assess overall study compliance, as required per Requirements for On-Site Monitoring of DAIDS Funded and/or Sponsored Clinical Trials, GCP, and FDA regulations 21 CFR Part 312:
http://www.niaid.nih.gov/LabsAndResources/resources/DAIDSClinRsrch/Documents/onsitemonitor_reqs.pdf

Study monitors will visit the site to complete the following:

- Assess compliance with the study protocol, Good Clinical Practices (GCP) guidelines, and applicable regulatory requirements, including US CFR Title 45 Part 46 and Title 21 Parts 50, 56, and 312
- Review informed consent forms, procedures, and documentation
- Perform source document verification to ensure the accuracy and completeness of study data
- Verify proper collection and storage of biological specimens

- Verify proper storage, dispensing, and accountability for investigational study products
- Assess implementation and documentation of internal site quality management procedures
- Assess site staff training needs

Site investigators will allow study monitors to inspect study facilities and documentation (e.g., informed consent forms, clinic and laboratory records, other source documents, case report forms), as well as observe the performance of study procedures. Investigators also will allow inspection of all study-related documentation by authorized representatives of the DAIDS, Sponsor and US regulatory authorities. A site visit log will be maintained at the study sites to document all visits. The outcomes of the monitoring visits and the subsequent reports of resolutions of any identified problems will be provided to the Sponsor of the IND application, and also submitted to DAIDS Clinical Operations.

17. HUMAN SUBJECTS PROTECTION

The investigators will make efforts to minimize risks of these products to human subjects. Participants will take part in a thorough informed consent process throughout their participation in the study. Before beginning the study, the investigators will have obtained IRB approval and the protocol will have been submitted to the FDA. The investigators will permit audits by the NIH or the FDA or any of their appointed agents.

17.1 INSTITUTIONAL REVIEW BOARD/ETHICS COMMITTEE

IRB continuing review and approval will be obtained from the reviewing EC/IRB at least once per year. If IRB approval expires (e.g., lapse in continuing review), all ongoing research activities will stop, unless the investigator determines that it is in the best interested of already-enrolled participants to continue their study-related activities. New participants will not be enrolled on the study until IRB approval to continue the research is obtained.

17.2 SPECIAL POPULATIONS

Study staff will offer screening to eligible women of all ethnic and racial groups. Members of the study staff are not seeking the screening or enrollment of women in special or vulnerable populations. The following Section also discusses special considerations for male partners of participants.

17.2.1 Men

Men are not included as subjects in the study because the study is testing a vaginal application of the study product. The male sexual partners of women participating in this study will not be consented or monitored for several reasons. Protocol-specified guidelines for abstinence and condom use are expected to protect male partners from exposure to the study product.

17.2.2 Children

The NIH has mandated that children be included in research trials when appropriate. This study will enroll women aged from 18 up to 21 who are able to give informed consent. This study meets "Justifications for Exclusion" criteria for younger children as set forth by the NIH. Specifically, "the research topic to be studied is irrelevant to (young) children" and "a separate, age-specific study in (adolescent) children is warranted and preferable" at a later time.

17.2.3 Prisoners

Prisoners will not be included in this study (for screening or enrollment). Any participants incarcerated during the course of participation in the trial will not be followed during their incarceration, and will be discontinued from the study. Participants who have been released from incarceration will be permitted to return for any protocol specified follow-up or safety visits per the guidelines of the local IRB.

17.2.4 Pregnant women

Pregnancy is an exclusion criterion because there is no current knowledge on the safety of the product during pregnancy; At the Screening Visit, a urine pregnancy test will be performed on all women. During the informed consent process, women will be informed that the study film is not known and not expected to prevent pregnancy and that the effect of the study film on a developing human fetus is not known. All enrolled participants will be required to be using a reliable method of contraception, such as hormonal contraception, intrauterine device or sterilization. Women who become pregnant during the study period following randomization and exposure to study product will not be excluded from analysis. Every effort will be made to maintain periodic contact with the participant after study termination until the completion of the pregnancy in order to determine the pregnancy outcome, and to obtain records from the facility at which the outcome occurred.

17.3 GOOD CLINICAL PRACTICE

The procedures set out in this study protocol are designed to ensure that all relevant parties abide by the principles of the Good Clinical Practice (GCP) guidelines of the ICH, and will adhere to DAIDS policies. The study will also be carried out in keeping with local legal requirements.

17.4 INFORMED CONSENT

Written informed consent will be obtained from all potential study participants at Visit 1, prior to the initiation of any study-related procedures. The informed consent process will give individuals all of the relevant information they need in order to decide whether to participate, or to continue participation, in this study. Potential research participants will be permitted to ask questions and to exchange information freely with the study investigators. Only listed study investigators may obtain informed consent from potential study participants. The investigators will keep research participants fully informed of any new information that could affect their willingness to continue study participation.

17.5 STORED SAMPLES

17.6 SPECIMEN STORAGE AND POSSIBLE FUTURE RESEARCH TESTING

Participants will be consented for future use of cervicovaginal lavage (CVL) specimens and blood samples. Any leftover CVL samples will be stored at the Anderson Laboratory, and serum at LeafBio, Inc. for an indefinite period of time (addresses given in Section 3).

Any results from research done on leftover specimens will not be placed in health records and will be kept confidential. Informed consent will give participants the option to withdraw their consent for use of their specimens for future research. The language and format employed in the screening and enrollment consents for these purposes are an IRB-approved means commonly employed in studies performed at this and other study sites within our institution to obtain permission for use of stored samples. All primary study endpoints, protocol-specified testing, and QA/QC testing will be ascertained prior to any additional testing of stored specimens. When all laboratory assays have been completed and the study has been closed, the PI for the study will notify the Anderson Laboratory and Mapp Laboratory Managers to discard all samples from participants in the study who chose to have their samples destroyed at the end of the study. These samples will be discarded in the appropriate manner and the disposition will be documented. The applicable NIH Data Sharing Policy (http://gds.nih.gov/03policy2.html) will be followed for data obtained from stored specimens.

17.7 RISK/BENEFIT STATEMENT

17.7.1 Risks

It is not expected that this trial will expose human subjects to unreasonable risk. The intervention used in this study is unlikely to cause uncomfortable side effects and is only given daily for one day for Segment A participants, and daily for seven days for Segment B participants. However, since there is no prior experience using the study film in humans, unanticipated side effects including irritation, allergic reactions, or other toxicities may occur.

There is no risk of false positive results with the OraQuick Rapid HIV 1/2 test used in this study due to absorption of the anti-HIV MAb in the MB66 film, because the test does not include an HIV gp120 target. There is a theoretical risk of false positive results with HIV antibody tests that do contain gp120. However, this risk is judged to be extremely low: First, the systemic bioavailability of previously tested intravaginal anti-HIV MAbs is very low, (undetectable in plasma with assays sensitive to the low mcg/mL level, (Morris et al 2010 and Morris et al 2012)). Second, The University of Washington Virology Specialty Laboratory (UW-VSL, Dr. Robert Coombs), in conjunction with the HVTN and the VRC/NIH, tested HIV uninfected human plasma samples spiked with VRC01 at 200 mcg/mL, 50 mcg/mL, 12.5 mcg/mL, 3.1 mcg/mL and 0.8 mcg/mL, and did not cause a positive test result in several standard antibody-based HIV-1/2 diagnostic tests used in the US (personal communication Dr. John Hural, HVTN/Fred Hutchinson Cancer Research Center, Seattle, WA, USA). Moreover, if a participant were to be tested with a gp120-based HIV test outside the study within a few months of using the study film, in the remote chance of a false positive result, standard follow-up testing done as part

of that HIV test would show that the result was a false positive. Such follow up testing is done before reporting back the screening test findings, thus avoiding the concerns that would be raised by receiving an initial positive screening test result.

Speculum exams may cause mild discomfort. Cervicovaginal lavage has been associated with mild discomfort secondary to the introduction of sterile fluid into the vagina and its removal. The Tear Flo collection has been widely used in vaginal research studies, and is well tolerated. Phlebotomy may lead to discomfort, feelings of dizziness or faintness, and/or bruising, swelling and/or infection. Approximately two and a half teaspoons of blood will be collected at the Screening Visit and less than two teaspoons at subsequent visits.

Disclosure of sexually transmitted infection (STI) may cause sadness or depression in participants. Disclosure of HIV-positive status has been associated with depression, suicidal ideation, and denial as well as social isolation. Participation in clinical research includes the risks of loss of confidentiality and discomfort with personal nature of questions.

17.7.2 Social Impact Events

Individuals enrolled in this study may experience personal problems resulting from the study participation. Such problems are termed social impact events. Although study sites will make every effort to protect participant privacy and confidentiality, it is possible that participants' involvement in the study could become known to others, and that participants may experience stigmatization or discrimination as a result of being perceived as being HIV-infected or at risk for HIV infection. For example, participants could be treated unfairly, or could have problems being accepted by their families and/or communities. Problems may also occur in circumstances in which study participation is not disclosed, such as impact on employment related to time taken for study visits.

In the event that a participant reports a social impact event, every effort will be made by study staff to provide appropriate assistance, and/or referrals to appropriate resources. Social impact events are documented and reviewed on a scheduled basis by the protocol team leadership with the goal of reducing their incidence and enhancing the ability of study staff to mitigate them when possible.

Social impact events that are judged by the IoR/designee to be serious, unexpected, or more severe or frequent than anticipated, will be reported to the responsible site’s EC/IRB promptly, or otherwise in accordance with the EC/IRB’s requirements.

17.7.3 Benefits

It is unlikely that participants will directly benefit from the use of the study film.

Participants and others may benefit in the future from information learned from this study. Specifically, information learned in this study may lead to the development of safe and effective interventions to prevent HIV transmission. Participants also may appreciate the opportunity to contribute to the field of HIV prevention.

Participants will receive HIV/STI risk reduction counseling, HIV and STI testing, physical exam, pelvic exam, and routine laboratory testing related to blood, liver, and kidney function. Participants may be provided or referred for STI treatment in accordance with CDC guidelines. For other medical conditions identified as part of the study screening and/or follow-up procedures, participants will be referred to other sources of care available in their community. Some participants may have the opportunity to access expedient treatment and decreased morbidity due to early diagnosis and treatment of abnormalities in serology, blood count, liver or kidney function tests. Pap smear may offer the opportunity for early detection of a cervical and/or vaginal abnormality with expedient referral if an abnormality is detected. Lastly, the participant may appreciate the opportunity to contribute to the body of knowledge in the field of microbicide research. However, there is no guarantee that participants will receive any of these benefits.

17.8 Compensation

Participants will not be charged for any of the study visits, study supplies or examinations. There are no costs to participants in this study other than their time. Pending IRB approval of these compensation guidelines, women will be compensated for their time and inconvenience and for their travel needs while participating in the protocol. The approved amounts of compensation will be given out at each visit. The visits will be prorated and partial payment given in the event that the participant only completes a portion of the study visits. The following proposed compensation amounts were created based on common institutional practice for studies investigating vaginal products.

Segment A participants

Visit 1	$50
Visit 2	$100
Visit 3	$50
Visit 4	$50

Segment B participants:

Visit 1	$50
Visit 2	$100
Visit 3	$50
Visit 4	$50
Visit 5	$50

In addition a parking pass will be provided to participants as needed.

17.9 Participant Confidentiality

Members of the study staff are all trained in participant confidentiality. All participant-related information including case report forms, laboratory specimens, evaluation forms, reports, etc., will be kept strictly confidential. All records will be kept in a secure, double-locked location and

only research staff will have access to the records. Participants will be identified only by means of a coded number specific to each participant. All computerized databases will identify participants by numeric codes only, and will be password-protected. Upon request, participant records will be made available to the study sponsor, the sponsor's monitoring representative, representatives of a participating pharmaceutical sponsor, and applicable regulatory entities.

17.10 Critical Event Reporting

Critical events include the following classes of events: unanticipated problems involving risks to participants or others, serious noncompliance, continuing noncompliance, suspension or termination of EC/IRB approval, and suspected research misconduct.

Critical Events occurring any time during conduct of the study will be reported to DAIDS, the Miriam Hospital IRB, and the FDA within one week of recognition.

17.11 Communicable Disease Reporting

Study staff members will comply with all local requirements to report communicable diseases including chlamydia, gonorrhea, syphilis, and HIV identified among study participants to the Rhode Island Health Department. Study investigators will include discussion of mandated reporting during the study informed consent process.

17.12 Access to HIV-Related Care

The investigators do not expect a screening population at high risk for HIV infection. However, trained clinical staff will refer subjects who test positive or indeterminate via the HIV antibody screen test to a physician for follow-up testing and care. Participants who have positive or indeterminate results will have standard post-test counseling as well as limited follow-up confirmatory testing provided by the study. Referral for additional counseling related to testing or diagnosis will occur if needed or requested by the participant. Participants who do not have an identified primary physician will be referred to the Rhode Island Health Department for follow-up testing and counseling via the ACHD HIV Program.

17.13 Study Discontinuation

NIAID, LeafBio, Inc., the US FDA, other government or regulatory authorities, or The Miriam Hospital Institutional Review Board may discontinue this study at any time. Ongoing safety monitoring will track the incidence of AEs and SAEs. In the event of an abnormal number of reported AEs and/or SAEs judged to be related to study film, or any other condition deemed as an emergency event by the study staff, the PSRT will direct the Principal Investigator to initiate a temporary hold on further enrollment.

17.14 Approval of Study Protocol

The study protocol and/or other appropriate documents will be submitted to the IRB and the FDA in accordance with local legal requirements prior to study initiation. All required approvals

must be documented and communicated to the Sponsor and the Investigator before the first participant is enrolled in the study at a site.

17.15 AMENDING THE PROTOCOL

Investigators should follow this protocol as written unless the Investigator determines that immediate deviation is required to protect the rights and welfare of a subject. All protocol amendments will be submitted to the DAIDS MO, the DAIDS Regulatory Support Center, and the local IRB prior to the implementation of an amendment.

17.16 CONFIDENTIALITY

All study findings and documents will be regarded as confidential. The Investigator and study staff must not disclose such information without prior written approval from the Sponsor.

18. ADMINISTRATIVE PROCEDURES

The study proposal for funding, this protocol, the informed consent documents, data collection forms, CASI interview questions, and advertising flyers are all reviewed by the Miriam Hospital Institutional Review Board prior to enrollment of participants in the study.

18.1 PROTOCOL REGISTRATION

Prior to implementation of this protocol, and any subsequent full version amendments, the site will have the protocol and the protocol consent form approved, as appropriate, by their local institutional review board (IRB)/ethics committee (EC) and any other applicable regulatory entity (RE). Upon receiving final approval, the site will submit all required protocol registration documents to the DAIDS Protocol Registration Office (DAIDS PRO) at the Regulatory Support Center (RSC). The DAIDS PRO will review the submitted protocol registration packet to ensure that all of the required documents have been received.

Site-specific informed consent forms (ICFs) will be reviewed and approved by the DAIDS PRO, and sites will receive an Initial Registration Notification when the DAIDS PRO receives a complete registration packet. Receipt of an Initial Registration Notification indicates successful completion of the protocol registration process. Sites will not receive any additional notifications from the DAIDS PRO for the initial protocol registration. The study will be initiated only after final approval from DAIDS.A copy of the Initial Registration Notification should be retained in the site's regulatory files.

Upon receiving final IRB/EC and any other applicable RE approvals for an amendment, the site should implement the amendment immediately. The site is required to submit an amendment registration packet to the DAIDS PRO at the RSC. The DAIDS PRO will review the submitted protocol registration packet to ensure that all the required documents have been received. Site-specific ICF(s) WILL NOT be reviewed and approved by the DAIDS PRO, and the site will

receive an Amendment Registration Notification when the DAIDS PRO receives a complete registration packet. A copy of the Amendment Registration Notification should be retained in the site's regulatory files.

For additional information on the protocol registration process and specific documents required for initial and amendment registrations, refer to the current version of the DAIDS Protocol Registration Manual.

18.2 REGULATORY OVERSIGHT

LeafBio, Inc. holds the IND for the MB66 drug product and for this clinical study, and bears overall responsibility for the conduct of this study.

18.3 STUDY COORDINATION

On site data management responsibilities will reside with the study staff at The Miriam Hospital. This will include managing and retaining clinical charts, source documents, and CRFs, and transfering photocopies of CRFs to the data management coordinator at Boston University. Data entry, query generation, database maintenance, will be the primary responsibility of Dr. Joseph Politch at Boston University. Data analysis will be the responsibility of Dr. Politch and study biostatistician Dr. Ralph D'Agostino Jr., the responsibility for completeness and consistency review (query generation) and database entry.

18.4 CLINICALTRIALS.GOV

This protocol is subject to the Food and Drug Administration Amendments Act of 2007 (FDAAA), and will be registered in ClinicalTrials.gov.

18.5 PROTOCOL COMPLIANCE

All protocol amendments will be submitted to and approved by The Miriam Hospital and Boston University Medical Center IRBs, and the DAIDS Medical Officer prior to the implementation of an amendment. At the DAIDS Medical Officer's discretion, submission to and approval by the DAIDS PSRC may also be required.

18.6 TRAINING PROCEDURES

Only study staff trained and experienced in HIV pre-test and post-test counseling and who are investigators on this study will provide these study procedures. Approved written materials consistent with the local clinical standard of care will support pre-test and post-test counseling.

19. PUBLICATION POLICY

Publication of study results will be governed by DAIDS policies. The investigators will submit any presentation, abstract, or manuscript to DAIDS for review prior to submission.

20. REFERENCES

1. Barnabas RV, Celum C. Infection Co-Factors in HIV-1 Transmission Herpes Simplex Virus Type-2 and HIV-1: New Insights and Interventions. *Current HIV Research.* 2012; 10(3): 228-237.

2. Bennetto-Hood C, Johnson VA, King JR, Hoesley CJ, Acosta EP. Novel methodology for antiretroviral quantitation in the female genital tract. *HIV Clin Trials*. 2009 May-Jun; 10(3):193-9. doi: 10.1310/hct1003-193.

3. Bunge KE, Dezzutti CS, Macio I, Hendrix CW, Rohan LC, Marzinke MA, Devlin B, Meyn L, Spiegel H, Hillier SL . FAME-02: A Phase I trial to assess safety, PK, and PD of gel and film formulations of Dapivirine. *CROI.* 2014. Oral Abstract 42LB.

4. Casadevall A, Dadachova E, Pirofski L. Passive Antibody Therapy for Infectious Diseases. *Nature Reviews*. 2004; 2: 695-703.

5. ClinicalTrials.gov. "VRC 601: A Phase I, Open-Label, Dose-Escalation Study of the Safety and Pharmacokinetics of a Human Monoclonal Antibody, VRC HIVMAB060-00-AB (VRC01), With Broad HIV-1 Neutralizing Activity, Administered Intravenously or Subcutaneously to HIV-Infected Adults." Updated June 21, 2014. Accessed June 2014 via: <http://clinicaltrials.gov/ct2/show/NCT01950325?term=vrc601&rank=1>

6. De Logu A, Williamson RA, Rozenshteyn R, Ramiro-Ibañez F, Simpson CD, Burton DR, Sanna PP. Characterization of a type-common human recombinant monoclonal antibody to herpes simplex virus with high therapeutic potential. *J Clin Microbiol*. 1998; 36: 198-204.

7. Diekman AB, Norton EJ, Klotz KL, Westrook VA, Herr JC. Immunol Rev. 1999; 171:203-11.

8. Dereuddre-Bosquet N, Morellato-Castillo L, Brouwers J, Augustijns P, Bouchemal K, Ponchel G *et al.* MiniCD4 microbicide prevents HIV infection of human mucosal explants and vaginal transmission of SHIV(162P3) in cynomolgus macaques. *PLoS Pathog*. 2012; 8(12):e1003071.

9. Fleming DT, Wasserheit JN. From Epidemiological Synergy to Public Health Policy and Practice: The Contribution of Other Sexually Transmitted Diseases to Sexual Transmission of HIV Infection. *Sexually Transmitted Infections*. 1999; 75: 3-17.

10. Freeman EE, Weiss HA, Glynn JR, Cross PL, Whitworth JA, Hayes RJ. Herpes Simplex Virus Type 2 Increases HIV Acquisition in Men and Women: Systematic Review and Meta-Analysis of Longitudinal Studies. *AIDS*. 2006; 20: 73-83.

11. Friend DR, Kiser PF. Assessment of Topical Microbicides to Prevent HIV-1 Transmission: Concepts, Testing, Lessons Learned. *Antiviral Research*. 2013; 99: 391-400.

12. Giritch A, Marillonnet S, Engler C, Van Eldik G *et al.* Rapid High-Yield Expression of Full-Size IgG Antibodies in Plants Coinfected With Noncompeting Viral Vectors. *PNAS*. 2006; 103(40): 14701-14706.

13. Harrison PF, Hemmerling A, Romano J, Whaley KJ, Holt BY. Developing Multipurpose Reproductive Health Technologies: An Integrated Strategy. *AIDS Research and Treatment*. 2013; 790154: 1-15.

14. Hefferon K. Plant-derived pharmaceuticals for the developing world. *Biotechnology Journal*. 2013; 8(10): 1193-1202.

15. Holt BY, Kilbourne-Brook M, Stone A, Harrison P, and Shields W. Multipurpose Prevention Technologies for Sexual and Reproductive Health: Gaining Momentum and Promise. Contraception. 2010, Vol 81: 177-180.

16. Huang Zuo. (2002). "*en bo ke zhi liao yin xie bing de xin ji yuan*". China Prescription Drug, **5**:54-6.

17. Huang J, Ofek G, Laub L, Louder MK *et al.* Connors M. Broad and potent neutralization of HIV-1 by a gp41-specific human antibody. *Nature*. 2012; 491(7424): 406-12.

18. John GC, Sheppard H, Mbori-Ngacha D, Nduati R, Maron D, Reiner M, Kreiss J. Comparison of techniques for HIV-1 RNA detection and quantitation in cervicovaginal secretions. *Acquir Immune Defic Syndr*. 2001 Feb 1; 26(2):170-5.

19. Kouokam JC, Huskens D, Schols D, Johannemann A, Riedell SK, Walter W *et al*. Investigation of griffithsin's interactions with human cells confirms its outstanding safety and efficacy profile as a microbicide candidate. *PLoS One*. 2011; 6(8):e22635.

20. Lagenaur LA, Sanders-Beer BE, Brichacek B, Pal R, Liu X, Liu Y *et al*. Prevention of vaginal SHIV transmission in macaques by a live recombinant Lactobacillus. *Mucosal Immunol*. 2011; 4(6): 648-57.

21. Lagenaur LA, Villarroel VA, Bundoc V, Dey B, Berger EA. sCD4-17b bifunctional protein: extremely broad and potent neutralization of HIV-1 Env pseudotyped viruses from genetically diverse primary isolates. *Retrovirology*. 2010; 16: 7-11.

22. Li Y, O'Dell S, Walker LM, Wu X, Guenaga J, Feng Y et al. Mechanism of Neutralization by the Broadly Neutralizing HIV-1 Monoclonal Antibody VRC01. *Journal of Virology*. 2011; 85(17): 8954-8967.

23. Mauck CK, Lai JJ, Weiner DH, Chandra N, Fichorova RN, Dezzutti CS, Hillier SL, Archer DF, Creinin MD, Schwartz JL, Callahan MM, Doncel GF. Toward early safety alert endpoints: exploring biomarkers suggestive of microbicide failure. *AIDS Res Hum Retroviruses.* 2013 Nov; 29(11):1475-86.

24. Mayer KH, Venkatesh KK. Interactions of HIV, other sexually transmitted diseases, and genital tract inflammation facilitating local pathogen transmission and acquisition. *Am J Reprod Immunol*. 2011 Mar; 65(3):308-16.

25. Mburu N, Obiero JA, Waititu K, Mwaura BN, Orawo JO, Farah IO, Mwethera PG.. Safety studies of a recently developed microbicidal contraceptive gel (UniPron) in female baboons (Papioanubis): original research article. *African journal of reproductive health*. 2009; 13(4): 95-104.

26. Moog C, Dereuddre-Bosquet N, Teillaud JL, Biedma ME *et al*. Protective effect of vaginal application of neutralizing and nonneutralizing inhibitory antibodies against vaginal SHIV challenge in macaques. *Mucosal Immunol*. 2014 Jan; 7(1):46-56.

27. Morris G, Chindove S, Woodhall S, Wiggins R, Vcelar B, Lacey C. A prospective randomized double blind placebo-controlled phase 1 pharmokinetic and safety study of a vaginal microbicide gel containing three potent broadly neutralizing monoclonal antibodies (2F5, 2G12, 4E10) (MabGel). *Microbicides.* 2010 (Pittsburgh, PA) abstract LB1.

28. Morris, GC. Mabgel-1: C2F5, C4E10, C2G12 as a vaginal microbicide. Doctoral Thesis, Hull York Medical School, December 2012.

29. Owen DH, Katz DF. A vaginal fluid simulant. Contraception. 1999 Feb;59(2):91-5.

30. Quesnel A, Cu-Uvin S, Murphy D, Ashley RL, Flanigan R, Neutra MR. Comparative analysis of methods for collection and measurement of immunoglobulins in cervical and vaginal secretions of women. *J Immunol Methods.* 1997 Mar 28; 202(2):153-61.

31. Raymond E, Alvarado G, Ledesma L, *et al*. Acceptability of two spermicides in five countries. *Contraception*. 1999; 60: 45-50.

32. Reichert JM. Monoclonal Antibodies as Innovative Therapeutics. *Curr Pharmaceut Biotechnol.* 2008;9:423-430.

33. Reichert JM. Marketed therapeutic antibodies compendium. *MAbs.* 2012 May; 4(3): 413.

34. Rosen RK, Morrow KM, Carballo-Diéguez A, Mantell JE et al. Acceptability of Tenofovir Gel as a Vaginal Microbicide Among Women in a Phase I Trial: A Mixed-Methods Study. *Journal of Women's Health*. 2008; 17(3): 383-392.

35. Sherlock CH, Lott PM, Moendy DM, Merrik L *et al*. Use of Sno Strip filter-paper wicks for collection of genital-tract samples allows reproducible determination of human immunodeficiency virus type 1 (HIV-1) RNA viral load with a commercial HIV-1 viral load assay. *J Clin Microbiol.* 2006; 44:1115-9.

36. Sherwood JK, Zeitlin L, Whaley KJ, Cone RA, Saltzman M. Controlled release of antibodies for long-term topical passive immunoprotection of female mice against genital herpes. *Nat. Biotech*. 1996; 14: 468-471.

37. Steiner M, Spruyt A, Joanis C, *et al*. Acceptability of Spermicidal Film and Foaming Tablets Among Women in Three Countries. *International Family Planning Perspectives.* 1995; 21: 104-107.

38. Strasser R, Stadlmann J, Schahs M, Steigler G *et al.* Generation of Glyco-Engineered *Nicotiana Benthamiana* for the Production of Monoclonal Antibodies with Homogenous Human-Like N-Glycan Structure. *Plant Biotechnology Journal*. 2008; 6(4): 392-402.

39. U.S. Department of Health and Human Services, National Institutes of Health, National Institute of Allergy and Infectious Diseases, Division of AIDS. Division of AIDS (DAIDS) Table for Grading the Severity of Adult and Pediatric Adverse Events, Version 2.0. [November 2014]. Clarification Documents for the Division of AIDS (DAIDS) Table for Grading the Severity of Adult and Pediatric Adverse Events Version 2.0 - November 2014.

40. Female Genital Grading Table for Use in Microbicide Studies (Addendum 1 to the DAIDS Table for Grading Adult and Pediatric Adverse Events, Version 1.0, November 2007 [Clarification dated August 2009]). http://rsc.techres.com/Document/safetyandpharmacovigilance/Addendum_1_Female_Genital_Grading_Table_v1_Nov_2007.pdf

41. Veazey RS, Shattock RJ, Pope M, Kirijan JC, Jones J, Hu Q, Ketas T, Marx PA, Klasse PJ, Burton DR, Moore JP. Prevention of virus transmission to macaque monkeys by a vaginally applied monoclonal antibody to HIV-1 gp120. *Nat Med*. 2003 Mar; 9(3):343-6.

42. Veselinovic M, Neff CP, Mulder LR, Akkina R. Topical gel formulation of broadly neutralizing anti-HIV-1 monoclonal antibody VRC01 confers protection against HIV-1 vaginal challenge in humanized mouse model. *Virology*. 2012; 432: 505-510.

43. Visness CM, Ulin P, Pfannenschmidt S, Zekeng L. Views of Cameroonian sex workers on a woman-controlled method of contraception and disease protection. *Int J STD AIDS.* 1998; 9: 695-9.

44. Walker LM, Huber M, Doores KJ, Falkowska E, Pejchal R, Juien J-P *et al.* Broadly neutralization coverage of multiple highly potent antibodies. *Nature.* 2011; 477: 466-470.

45. Wang YY, Kannan A, Nunn KL, Murphy MA, Subramani DB, Moench T, Cone R, Lai SK. IgG in cervicovaginal mucus traps HSV and prevents vaginal Herpes infections. *Mucosal Immunol*. 2014 Feb 5.

46. Zeitlin L, Olmsted SS, Moench TR, Co MS, Martinell BJ, Paradkar VM *et al*. A humanized monoclonal antibody produced in transgenic plants for immunoprotection of the vagina against genital herpes. *Nat. Biotechnol*. 1998; 16: 1361–136.

47. Zeitlin L, Palmer C, Whaley KJ. Preventing sexual transmission of HSV and HIV: the challenge for active and passive immunization of mucosal surfaces. *Biotechnol Genet Eng Rev*. 2002; 19:121-38.

48. Zeitlin L, Whaley KJ, Hegarty TA, Moench TR, Cone RA. Tests of vaginal microbicides in the mouse genital herpes model. *Contraception*. 1997; 56: 329-335.

49. Zeitlin L, Whaley KJ, Sanna PP, Moench TR, Bastidas R, De Logu A *et al.* Topically applied human recombinant monoclonal IgG1 antibody and its Fab and F(ab')2 fragments protect mice from vaginal transmission of HSV-2. *Virology.* 1996; 225: 213-215.

50. Zhao C, Connor-Stroud F, Sharma P, Oviedo-Moreno P, Whaley K, Bohorov O, Moench T, Anderson DJ, Villinger F. "Nicotiana-produced broadly neutralizing anti-HIV monoclonal antibodies as a microbicide strategy in Cynomolgus macaques 31st annual Symposium on Nonhuman Primate Models of AIDS." November 3-6. 2013, Atlanta GA.

51. Zhou T, Georgiev I, Wu X, Yang ZY, Dai K, Finzi A, Kwon YD, Scheid JF, Shi W, Xu L, Yang Y, Zhu J, Nussenzweig MC, Sodroski J, Shapiro L, Nabel GJ, Mascola JR, Kwong PD. Structural basis for broad and potent neutralization of HIV-1 by antibody VRC01. Science. 2010;329(5993):811-7.

21. APPENDICES

APPENDIX 1: SCHEDULE OF STUDY VISITS AND PROCEDURES 95
APPENDIX 2: HIV TESTING ALGORITHM 97
APPENDIX 3: INVESTIGATOR'S AGREEMENT 98
APPENDIX 4: INFORMED CONSENTS FOR SEGMENTS A AND B 99

Appendix 1: Schedule of Study Visits and Procedures

Table 19: Segment A Study Visits and Procedures

PROCEDURE	Visit 1 Screening	Visit 2 Enrollment (Day 0)	Visit 3 Follow-up (Day 1)	Safety Phone Call (Day 3-4)	Visit 4 Exit (Day 6-10)	Ad hoc extra safety visit (if unresolved safety issue at Visit 4)
Informed Consent	X					
HIV Pre-test Counseling	X				X	
HIV Post-test Counseling	X				X	
Screening Results	X	X				
Visit Interview	X	X	X	X	X	
Confirm eligibility	X	X				
Enroll		X				
Abstinence Counseling	X	X	X	X	X	
Log concomitant medications	X	X	X		X	X
Condom Counseling (and distribution prn)					X	X
Urine HCG	X	X			X	Δ
Urinalysis	X					
Urine Dipstick		X	X		X	X
Urine NAATS for CT, GC, Trichomonas	X		Δ		Δ	Δ
CBC, Liver/ Renal Panel	X		Δ		X	Δ
Syphilis screen (RPR)	X		Δ		Δ	Δ
Rapid HIV test	X				X	
Confirmatory HIV blood test (if rapid test is positive)	Δ		Δ		Δ	
Vaginal pH	Δ	X	X		X	Δ
Wet Prep	Δ	Δ	Δ		Δ	Δ
Gram stained vaginal smear		X	X		X	
Pap	Δ					
Serum VRC01 & HSV8 ELISAs	X		X		X	
Tear Flo for VRC01 & HSV8 antibodies		X (Pre dose and 1&4 hr post dose)	X		X	
Visual assessment of degree of film dissolution during pelvic exam		X (1&4 hr post dose)	X		X	
CVL for VRC01 & HSV8 levels; neutralizing activity; immune mediators	X		X		X	
Physical Exam	X	X	X		X	Δ
Vital Signs	X	X	X		X	
Gynecologic Exam	X	X	X		X	Δ
Blood	X		X		X	
Assess quantity of undissolved film		X (1&4 hr post dose)	X		X	
Study Product Administration		X				
Adverse Event Collection		X	X	X	X	X

X Procedure done
Δ Procedure done if indicated

Table 20: Segment B Study Visits and Procedures

PROCEDURE	Visit 1 Screen	Visit 2 Enrollment (Day 0)	Visit 3 Follow-up (Day 1)	Safety Phone Call (Day 3-4)	Visit 4 Follow-up (Day 7-8)	Visit 5 Exit Visit (Day 12-16)	Ad hoc extra safety visit (if unresolved safety issue at Visit 5)
Informed Consent	X						
HIV Pre-test Counseling	X					X	
HIV Post-test Counseling	X					X	
Confirm eligibility	X	X					
Enroll		X					
Visit Interview	X	X	X	X	X	X	
Log concomitant medications	X	X	X		X	X	X
Return of unused Study Films					X		
Abstinence Counseling	X	X	X	X	X		
Condom Counseling (and distribution prn)						X	X
Film insertion training or review		X	X				
Acceptability Questionnaire (CASI)					X		
Urine HCG	X	X			X	X	Δ
Urinalysis	X						
Urine Dipstick		X	X		X	X	X
Urine NAATS for CT, GC, Trichomonas	X		Δ		Δ	Δ	Δ
CBC, Liver/ Renal Panel	X		Δ		Δ	X	Δ
Syphilis screen (RPR)	X		Δ		Δ	Δ	Δ
Rapid HIV test	X					X	
Confirmatory HIV blood test (if rapid test is positive)	Δ				Δ	Δ	
Vaginal pH	Δ	X	X		X	X	Δ
Wet Prep	Δ	Δ	Δ		Δ	Δ	Δ
Gram stained vaginal smear		X	X		X	X	
Vaginal swab: Microbiome PCR		X	X		X	X	
Pap	Δ						
Serum VRC01 & HSV8 ELISAs	X		X		X	X	
Tear Flo for VRC01 & HSV8 antibodies		X (Pre dose and 1&4 hr post dose)	X		X	X	
Visual assessment of degree of film dissolution during pelvic exam		X (1&4 hr post dose)	X		X	X	
CVL for VRC01 & HSV8 levels; neutralizing activity; immune mediators	X		X		X	X	
Physical Exam	X	X	X		X	X	Δ
Vital Signs	X	X	X		X	X	
Gynecologic Exam	X	X	X		X	X	Δ
Blood	X		X		X	X	
Randomization		X					
Study Product Administration in clinic		X					
Study product & MEMS container instruction and dispensing for home dosing			X				
Adverse Event Collection		X	X	X	X	X	X

X Procedure done
Δ Procedure done if indicated

APPENDIX 2: HIV TESTING ALGORITHM

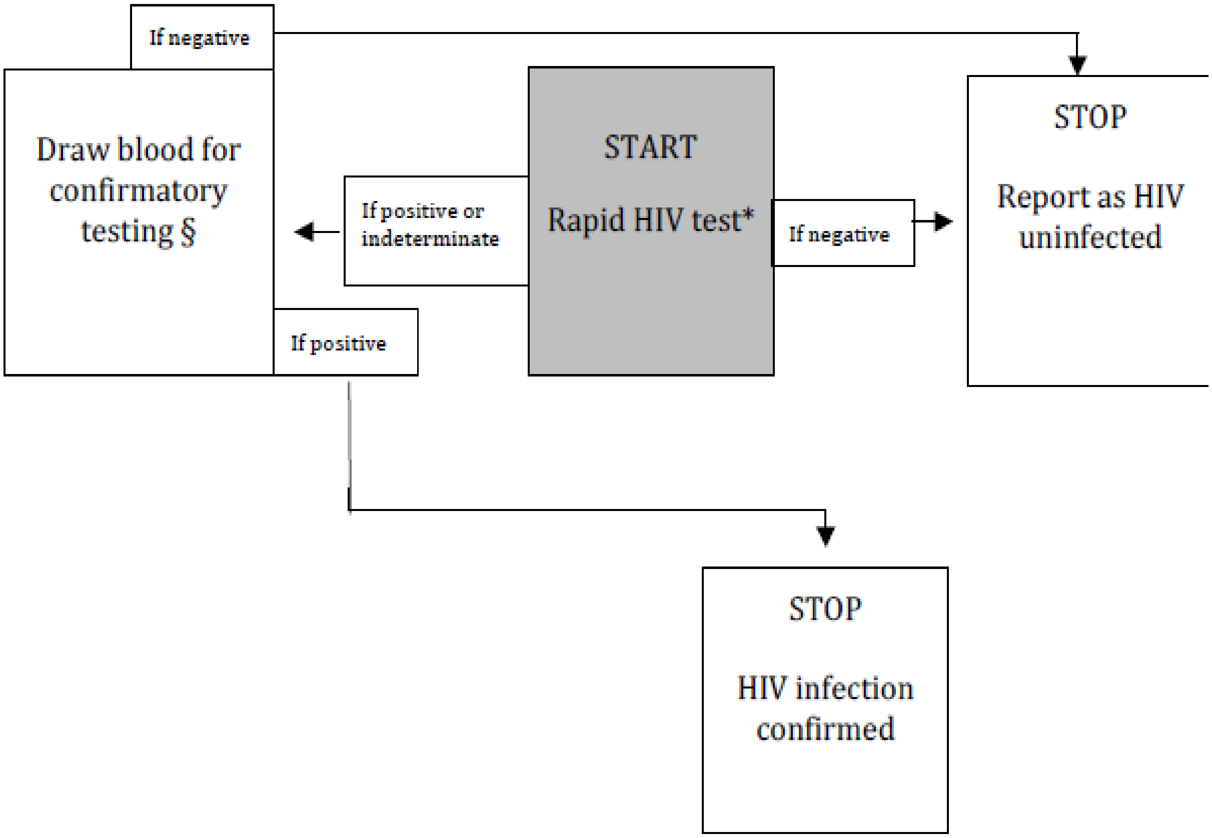

* OraQuick Advance Rapid HIV 1 / 2 antibody test
§ HIV 1/ 2 antigen/antibody combination assay

APPENDIX 3: INVESTIGATOR'S AGREEMENT

I understand that all documentation supplied to me by LeafBio, Inc. concerning this study will be kept in the strictest confidence. This documentation includes the study protocol, Investigator Brochure, Case Report Forms, and other scientific data.

The study will not commence without the prior written approval of a properly constituted IRB. No changes or significant deviations will be made to the study protocol without the prior written approval of LeafBio, Inc., and in some cases, the IRB, except where necessary to eliminate an immediate hazard to the research participant.

I have read, understood, and do agree to abide by all the conditions and instructions contained in this protocol, A ***Pre-Phase 1, Single Center Study to assess the safety of MB66, a Combined Anti-HIV (VRC01-N) and Anti-HSV (HSV8-N) Monoclonal Antibody Film for Vaginal Application as Microbicide*** (Protocol #: MB66-01).

____ ______________

Investigator Signature Date

____

Printed Name

____

Institution Name, Address, and Telephone Number

APPENDIX 4: INFORMED CONSENTS FOR SEGMENTS A AND B

Study Volunteer Initials

Lifespan Affiliate Site where research will be conducted

- ☐ Rhode Island Hospital
- ☐ Bradley Hospital
- ☒ The Miriam Hospital
- ☐ Newport Hospital
- ☐ Gateway Healthcare

Agreement to Participate in a Research Study
And Authorization for Use and Disclosure of Information

Committee # | Name of Study Volunteer

Study Title: A Phase 1, Single Center Study to assess the safety of MB66, a Combined Anti-HIV (VRC01) and Anti-HSV (HSV8) Monoclonal Antibody Film for Vaginal Application as Microbicide (MB66-01)

Study Number: MB66-01 – Segment A

Principal Investigators: Susan Cu-Uvin, M.D., 401-793-7152

Study Nurse: Helen Patterson, 401-793-4771

You are being asked to take part in a research study. All research studies at Lifespan hospitals (including Miriam Hospital) follow the rules of the state of Rhode Island, the United States government and Lifespan. Before you decide whether to be in the study, you and the researcher will engage in the "informed consent" process. During this process, the researcher will explain the purpose of the study, how it will be carried out, and what you will be expected to do if you participate. The researcher will also explain the possible risks and benefits of being in the study, and will provide other information. You should feel free to ask any questions you might have. The purpose of these discussions is for you to decide whether participating in the study is the best decision for you.

If you decide to be in the study, you will be asked to sign and date this form in front of the person who explained the study to you. This form summarizes the information you discussed. You will be given a copy of this form to keep.

1. <u>Nature and Purpose of the Study</u>

You are being asked to take part in a research project because you are a woman between the ages of 18 and 45 and in general good health.

Sex is the most frequent way HIV (Human Immunodeficiency Virus) and HSV-2 (herpes simplex virus) is transmitted. Condoms are a safe and effective way to prevent transmission of these viruses but for a variety of reasons people do not use them consistently. In heterosexual sex, women have to rely on their male partners to agree to use a condom. There is a need for a product that women can

use to prevent the transmission of HIV and HSV-2 that is convenient, easy to use, safe and effective.

This study will explore the safety and effectiveness of an investigational vaginal film as a way to deliver a medication that may prevent the transmission of viruses and other sexually transmitted diseases. The film is relatively small (about 2 inches square), flexible and flat, similar to wax paper. This film will contain a microbicide (a product that may reduce the chance of getting HIV and the herpes virus) that works when it comes in contact with the vagina tissue.

This study will help determine if this investigational vaginal product is safe and could be used to protect women against sexually transmitted infections. An investigational product means that it has not been approved by the Federal Food and Drug Administration (FDA) and is still being studied. We do not know if the product actually will reduce the chance of infection or how long the protection will last.

The study product, a vaginal film, works by dissolving when it comes in contact with vaginal fluid. The film contains human antibodies made by plants, and may be able to reduce a woman’s chance of getting HIV, the virus that causes AIDS (human immunodeficiency virus), and HSV, the virus that causes herpes (herpes simplex virus).

The immune system of humans and animals naturally make antibodies to fight against infection; but it can take a lot of time and money for scientists to get enough animal antibodies to help prevent HIV and herpes infections. Plants do not naturally make antibodies, but scientists have found a way to use plants to produce antibodies that humans can use. It will be quicker and cost less money to make antibodies in plants, and it may be safer because, unlike animals, plants do not carry viruses that can infect humans.

In this study, you will be asked to use a vaginal film that contains antibodies produced by the *Nicotiana benthamiana* plant (a relative of the “tobacco plant,” *Nicotiana tabacum*). While *Nicotiana benthamiana* does contain a small amount of nicotine, the plant goes through a cleaning process leaving only the antibodies (and small fragments of antibodies) that are then used in the vaginal film. The product that you will be using does not contain any nicotine and does not have any of the cancer causing effects of tobacco products. Antibodies made by this plant have already been shown to be effective at preventing HIV and herpes in laboratory studies. As we continue, when we say “drug” we are referring to this antibody, which is the active ingredient in the study product.

We expect to enroll about 8 subjects into this part of the study. The study is sponsored by LeafBio, Inc. and funded by the National Institutes of Health (NIH).

2. <u>Explanation of Procedures</u>: If you take part in this study, you will complete up to 4 study visits (including this screening visit) and one phone interview over the span of about 3 to 8 weeks. You will be provided a food voucher for the six hour enrollment visit (visit 2). The visits are outlined below.

Visit 1 (Screening) – This visit may take up to 2 hours. During this visit you will review and sign this informed consent document. During this visit, the study nurse will give you detailed information

about the study and what you will be required to do, and answer any questions you may have. You will be asked to provide contact information and complete a screening interview.

This study requires a period of abstinence (not having sexual intercourse) that starts 5 days before your enrollment visit and continues until Visit 4, a week after you receive the study film. The importance of abstinence will be discussed and you will need to agree to not have sexual intercourse to take part in the study

After that visit, you can either continue not having sex or use condoms for vaginal intercourse for two weeks after that visit. During this time you cannot use a spermicide, a diaphragm or a NuvaRing as contraception. Acceptable forms of birthcontrol are: condom, hormonal contraception (pills, implants, injection, and patches), IUD and male or female sterilization..

You will also be required to abstain from <u>any</u> vaginal insertions such as douches and sex toys. You are allowed to use tampons during your period.

Procedures during this screening visit will include:

- You will be asked questions about your general health, any symptoms you may be having, and medications you are taking.
- You will have a physical exam that will include your height, weight, temperature, blood pressure and heart rate.
- If you are 21 or older and have NOT had a pap smear in the last 36 months (3 years), you will have a pap smear done.
- You will provide a urine sample to test for pregnancy, urinary infection, and STDs.
- You will have about three tablespoons of blood drawn for routine blood tests, screening for syphilis, and for before-treatment levels of the study drug.
- Pelvic exam and tests for sexually transmitted diseases (STDs).
- During the pelvic exam you will have a procedure called a cervicovaginal lavage (CVL). In this procedure, about 1 tablespoon of a salt solution (which matches the normal salt content in your own body fluids), will be put in the cervicovaginal area (the area at the top of the vagina where the cervix is located) for a few seconds and then collected for testing.
- A test called a "Rapid HIV test" will be done. A padded device (similar to a toothbrush) is placed between your cheek and your gum. After gently swabbing your mouth, the padded end is then inserted into a solution vial: the result appears on the result window within 20 minutes. As part of the testing procedures, the research staff will ask you questions about your sexual behavior and/or treatment history. The questions the research nurse asks will be similar to questions you might normally be asked when being examined by a doctor regarding reproductive and sexual health. The questions and discussion you have with the research staff are part of a pre/post-test counseling procedure required by the Rhode Island Department of Health when testing for HIV and other sexually transmitted diseases. These questions and discussion will include talking about your sexual behavior.

The pregnancy, HIV and STD tests must be negative for you to enter the study.

If you are menstruating at the time of a study visit, the visit will be rescheduled. We will try to schedule all study visits in between the periods of bleeding.

Visit 2 (Enrollment) This visit will be scheduled within 45 days of the screening visit and may last up to 6 hours. During this visit the study staff will ask you about changes to your contact information, medications, and about any symptoms or other health problems you are currently experiencing. The study staff will talk to you about abstinence, review the importance of abstinence during this study, and give you an interview.

Procedures during the enrollment visits will include:

- You will have a short physical exam including temperature, blood pressure and heart rate.
- A urine sample will be collected to test for pregnancy and to test for infection.
- You will have a pelvic exam to look for any signs of bleeding and 3 separate samples of vaginal fluid will be taken using vaginal swabs to measure bacteria in your vagina and to measure the pH (level of acidity) of your vagina. Tear Flo test strips (pieces of absorbent paper) will be held in contact with the vaginal tissue at four places to sample drug level before the vaginal film is inserted. The vaginal film study product will be inserted during this exam.
- At 1 and 4 hours after the insertion of the vaginal film, the Tear Flo sampling procedure will be repeated to test for the drug levels in the vaginal fluid.

Visit 3 (Follow-up) – This visit will occur one day following visit 2 – This visit may last up to 2 hours. During this visit the study staff will talk to you about abstinence and review the importance of abstinence during this study and will ask you about any symptoms or other health problems you have had or are currently experiencing, and about your medications.

Procedures during this visit will include:

- You will have a short physical exam including temperature, blood pressure and heart rate.
- A urine sample will be tested for urinary tract infection.
- Up to about 3 tablespoons of blood will be taken to test for the presence of the study drug. Other tests may be performed on the blood, if indicated.
- You will have a pelvic exam to look for any signs of bleeding and 3 separate samples of vaginal fluid will be taken using vaginal swabs to measure bacteria in your vagina and to measure the pH (level of acidity) of your vagina. Tear Flo test strips will be used to sample drug levels as described for Visit 2.
- A CVL procedure will be done (as described in the Screening visit section).

Phone call 2 to 3 days after Visit 3 – The study staff will call you to check how you are doing and if you are having any vaginal symptoms or other problems. If you are having symptoms that make you or the study staff concerned that you need to be examined, you will be asked to come in to the clinic for an extra visit. You will be reminded of the need to be abstinent.

Visit 4 (Final visit) - This visit will occur 1 week after Visit 2 –The study staff will provide you with condoms and review the need to use condoms (or remain abstinent) an additional 2 weeks (until

Study day 21). They will ask you about any symptoms or other health problems you have had or are currently experiencing, and about your medications.
Procedures during this visit will include:

- You will have a short physical exam including temperature, blood pressure and heart rate.
- A rapid HIV-1 test and counseling before and after the test
- A urine sample will be collected to test for pregnancy and to test for infection.
- About 3 tablespoons of blood will be taken to test for the presence of the study drug and for other routine blood tests. Other tests may be performed on the blood if indicated.
- You will have a pelvic exam to look for any signs of bleeding and 3 separate samples of vaginal fluid will be taken using vaginal swabs to measure bacteria in your vagina and to measure the pH (level of acidity) of your vagina. Tear Flo test strips will be used to sample drug levels as described in Visit 2.
- A CVL procedure will be done (as described in the Screening visit section).

Possible additional visits
If there are any unresolved safety concerns, you may be asked to return to the clinic for a follow-up visit.

Participants who test positive for HIV at screening or during the study will have further evaluation and will be referred to an HIV treatment provider for care. If HIV infection is found, you will not be able to stay in the study.

If you test positive for a sexually transmitted disease, Dr. Cu-Uvin will provide treatment based on the Centers for Disease Control (CDC) guidelines or she will refer you for treatment by another provider.

Participants who have a positive pregnancy test also cannot enter or stay in the study. A referral for obstetrical care will be made and we will ask you to sign a medical release form so that we may inquire about the status of your pregnancy and the health of the newborn.

Procedures for Early Termination or Withdrawal Visits
If for any reason your study participation ends early, you will come back to the clinic for one more visit which will include the following:

- You will have a short physical exam including temperature, blood pressure and heart rate.
- A rapid HIV-1 test and counseling before and after the test
- A urine sample will be collected to test for pregnancy and to test for infection.
- About 3 tablespoons of blood will be taken to test for the presence of the study drug and for other routine blood tests. Other tests may be performed on the blood if indicated.
- You will have a pelvic exam to look for any signs of bleeding and 3 separate samples of vaginal fluid will be taken using vaginal swabs to measure bacteria in your vagina and to measure the pH (level of acidity) of your vagina. Tear Flo test strips will be used to sample drug levels as described in Visit 2.
- A CVL procedure will be done (as described in the Screening visit section).

<u>Costs for participating in this study</u>

There will be no cost to you for participating in this study. Visits, procedures and medication that are part of the study procedures will be provided by the study.

However, if you receive services that are not part of this study and are considered routine clinical services, these services will be billed to your health insurance company. You will be responsible for paying any deductibles, co-payments, or co-insurance that are a normal part of your health insurance plan. If you do not have health insurance, you will be responsible for those costs. These services include treatment for HIV, STDs and pre-natal care.

Compensation

You will be compensated for your time and travel. You will be compensated $50 for each visit with the exception of visit 2 for which you will be compensated with $100. For visits where you will receive $100, payment will be sent to you in the form of a check. You will receive a voucher worth $50 for the other visits which can be redeemed at the Cashier's Office of The Miriam Hospital.

3. Discomforts and Risks

Cervicovaginal lavage and pelvic exams

You may experience slight discomfort or pressure when the speculum is inserted. There is a risk of infection, but it is minimized by using proper clean procedures.

MB66 (vaginal film)

Before human studies, products are tested in laboratory animals. Animals are given higher doses of the drug to help find out the amount of drug to use in humans. Some of the laboratory animals given MB66 had bleeding, inflammation and/or ulceration of the vaginal tissue. Vaginal bleeding or spotting and inflammation are possible risks with the use of MB66. Since this is the first time this drug will be used in humans, it is important to know there may be risks that are not yet known, including irritation, allergic reactions, or other side effects. There is a small chance that use of the study film could temporarily cause certain types of HIV tests to become "false positive", (meaning the test reading was positive, but you were not infected with HIV). This could happen if you absorbed enough of the antibody against HIV in the study film into your body to temporarily make this kind of HIV test falsely positive. Such a false positive result might be present for a few months after use of the study film. During the study we will use an HIV test that cannot be made falsely positive by use of the study film. But, if you were to have a different kind of HIV test outside the study within a few months of using the study film, there is a possibility that that test could give a false positive result. If this were to happen, the standard follow-up testing done along with that HIV test would show that this was a false positive test and that you were not infected with HIV.

Other study risks

Condoms are required for any vaginal intercourse that occurs within the two weeks after the final study visit, to prevent exposure of a partner to any residual MB66 film and to prevent pregnancy. However, condoms are less effective contraceptives than hormonal

contraceptives, IUD, female sterilization, or sexual activity with a partner who has had a vasectomy. The greatest protection against pregnancy would be provided by a condom along with one of the methods listed above.

This investigation film has not been proved to prevent the transmission of HIV or HSV. It is important that you **always** use a condom to reduce your risk.

Interviews and questionnaire
You may feel embarrassed by some of the questions.

HIV and STD (sexually transmitted disease) test results
You may feel some anxiety or concern while waiting for your test results and feel sadness and depressed if you test positive for a sexually transmitted infection, including HIV. A positive HIV result may cause you to feel isolated, have thoughts of suicide or deny the results. You will have access to study personnel and the study clinician to discuss these concerns. If you need psychological services or other emotional support, you will be referred to a mental health provider by study personnel. Although animal testing indicates this is very unlikely, it is possible that the study drug may be absorbed into your blood and cause a temporarily false positive rapid HIV test. Follow up testing will show if it is a false positive test.

Blood draw
There may be bruising or swelling where the blood is drawn and you may experience light-headedness, discomfort and rarely, infection

Loss of confidentiality
While we will do all that we can to protect your privacy (described in section 9), there is a slight risk of loss of confidentiality resulting in social harm when participating in studies.

4. <u>Benefits</u>
It is unlikely that you will receive any benefit from your participation in this study. However, others may benefit from the information learned in this study, by the development of safe and effective ways to prevent HIV and herpes transmission.

5. <u>Alternative Therapies</u>
There are no alternative therapies. You may choose not to be in this study.

6. <u>Refusal/Withdrawal</u>
It is up to you whether you want to be in the study. You are not required to enroll or participate. If you decide to participate, you can always change your mind and quit at any time. If you decide not to be in the study, or if you quit later, you will still be able to get the health care services you

normally get. If you join, but later on the researcher or your doctor feels being in the study is no longer good for you, they may choose to take you out of the study before it is over. If new information becomes available that might change your mind about whether you want to stay in the study the researcher will share this information with you as soon as possible.

The study doctor may withdraw you from the study for your protection, or if you are unable to comply with study procedures. You would also be withdrawn from the study in the event of pregnancy or a positive HIV-1 test. You would also be withdrawn if you require a prohibited medication while on this study.

If your participation is discontinued because the study product is discontinued, we would like your permission to continue to follow you.

You would also be withdrawn if the study is stopped by the NIH, government or institutional agencies. In the case of pregnancy, you will be asked to sign a medical release form as mentioned on page 3. You have the right to withdraw your consent for medical record release by contacting the study doctor or nurse.

7. Medical Treatment/Payment in Case of Injury

A research injury is any physical or mental injury or illness caused by being in the study. If you are injured by a medical treatment or procedure you would have received even if you were not in the study, that is not a research injury. To help avoid research injury and added medical expenses, it is very important to follow all study directions carefully. If you do experience a research injury, Lifespan or the study doctor can arrange medical treatment for you. Such treatment will be paid for as described below.

If you have insurance and have a research injury that is not covered by the study, it is possible that some or all of the cost of treating you could be billed to your insurer. If your health insurance will not cover such costs, it is possible you would have to pay out of pocket. In some cases, Lifespan might be able to help you pay if you qualify for free care under Lifespan policy. However, Lifespan has no policy to cover payment for such things as lost wages, expenses other than medical care, or pain and suffering.

The study sponsor, LeafBio Inc, has no program to compensate or pay for a study related injury.

If you experience a medical injury, please call either the study nurse or doctor. Their phone numbers can be located on the first page of this form.

8. Rights and Complaints

Signing this form does not take away any of your lawful rights. If you have any complaints about this study, or would like more facts about the rules for research studies, or the rights of people who take part in research studies you may contact Janice Muratori in the Lifespan Office of Research Administration, at (401) 444-6246.

9. Confidentiality and Research Authorization for Use and Disclosure of Your Health Care Information

Your research records will be treated as private health care records and will be protected according to Lifespan privacy practices and policies that are based on state and federal law. In particular,

federal law requires us to get your permission to use or disclose (release your information to someone outside of Lifespan) your health information for research purposes. If you sign this form you agree to be in this research study and you permit the use and disclosure of your health information for the purpose of conducting the research, providing treatment, collecting payment and running the business of the hospital. This permission has no expiration date. You may withdraw from the study at any time. However, if you do not want the researchers to use or disclose any further information in this study you must cancel permission in writing and may do so at any time. If you cancel your permission, you will stop taking part in the study and no new information will be collected about you. However, if you cancel your permission, it will not apply to actions already taken or information already collected about you by the hospital or the researchers before you canceled your permission.

Generally, the entire research record and any medical records held by the hospital may be used and released for research purposes. The following people or businesses/companies/ might use, release, or receive such information:

- The researcher and their support staff;
- The study sponsor (LeafBio, Inc.), and the study funder (The National Institutes of Health)
- Doctors, nurses, laboratories and others who provide services to you or the sponsor in connection with this study;
- The company or section of the U.S. government that is paying for the study and others they hire to oversee, administer, or conduct the research;
- The United States Food and Drug Administration, the Department of Health and Human Services, the Office of Inspector General, and the Office of Civil Rights; European Medicines Agency
- People who volunteer to be patient advocates or research volunteer protectors;
- Members of the hospital's administrative staff responsible for reviewing, approving and administering clinical trials and other healthcare or research activities.
- Accrediting Organizations

There are times when the law might require or permit Lifespan to release your health information without your permission. For example, Rhode Island law requires researchers and health care workers to report abuse or neglect of children to the Department of Children, Youth and Families (DCYF) and to report abuse or neglect of people age 60 and older to the Department of Elderly Affairs. Newly diagnosed cases of HIV, chlamydia, gonorrhea, and syphilis will be reported to the RI Department of Health as required by state law.

All researchers and health care providers are required to protect the privacy of your health care information. Other people and businesses/organizations that are not health care providers are not required by law to do that so it is possible they might re-release your information.

You have the right to refuse to sign this form and not participate in the research. Your refusal would have no affect on your treatment, charges billed to you, or benefits at any Lifespan health care site. If you do not sign, you will not be able to enroll in the research study and will not receive treatment as a study participant.

If you decide to quit the study after signing this form (as described in Section 6) no new information will be collected about you unless you gave us permission to do so. However, the hospital or the researchers may continue to use information that was collected before you quit the study to complete analysis and reports of this research.

A description of this clinical trial will be available on *http://www.ClinicalTrials.gov*, as required by U.S. law. This Web site will not include information that can identify you. At most, the Web site will include a summary of the results. You can search this Web site at any time.

Optional: Participation in Specimen Banking

As a participant in the main study, we will be collecting specimens (samples of vaginal fluid and blood). In addition to being analyzed as part of the Main Study, your Specimen may be useful for future research purposes. You are being asked to agree to this optional component (the "Specimen Banking Component") of the study if you are willing to allow your Specimen to be saved or 'banked' for use in future research studies. If you agree to this optional Specimen Banking Component, you give permission for your Specimen to be stored in a specimen bank. Along with the specimens, portions of your personal health information collected as part of the Main Study will also be stored. Your Specimen and personal health information may be stored and analyzed at Lifespan; or, they may be shared with researchers at other institutions or companies that may store them and use them for their own research. It is very unlikely that any future research performed using your Specimen would benefit you directly. However, the research may provide important medical knowledge that in the future could help patients with different medical problems.

At this time, we do not know what future research studies may be done using your Specimen.
There is a very remote possibility that your Specimen and some associated data may become part of a process or product that ultimately has commercial value. For instance, the Specimen could be used to establish a cell line (a group of cells that are able to reproduce, sometimes indefinitely) that could be patented and licensed. There are no plans to provide financial compensation to you should this occur.

If you agree to this Specimen Banking Component, your Specimen will be stored for an indefinite period of time, until it is no longer usable. The Specimen may also be used to create a cell line, which would also be stored for an indefinite period of time. If you decide at some time in the future that you no longer wish your stored Specimen to be used in future studies, you have the right to request that the Specimen be withdrawn from the specimen bank. However, withdrawal cannot be guaranteed and may be impossible. For example, it is possible that the Specimen might no longer be identifiable as belonging to you, or it may have been used up, or it may already have been shared with other institutions or companies for their own research. To request withdrawal of your Specimen, please write to: Susan Cu-Uvin, MD., The Miriam Hospital, 164 Summit Ave, Bldg 1125 No. Main St.-Rear, Providence, RI 02906.

Refusal to participate in this optional Specimen Banking component will in no way affect your ability to receive any treatment or services offered as part of the Main Study, and will not have any effect on your other health care, the payment for your health care, or your health care benefits. **If you are willing to allow your Specimen to be banked for future research purposes, please indicate your consent by signing below.**

____
Study Volunteer Initials

____ ________
Signature of study volunteer/authorized representative Date

For more detail about your privacy rights see the Lifespan Joint Privacy Notice which has or will be given to you.

SIGNATURE

I have read this informed consent and authorization form. <u>ALL OF MY QUESTIONS HAVE BEEN ANSWERED, AND I WANT TO TAKE PART IN THIS RESEARCH STUDY.</u>

By signing below, I give my permission to participate in this research study and for the described uses and releases of information. *I also confirm that I have been now or previously given a copy of the Lifespan Privacy Notice*

This informed consent document expires on __________________.
DO NOT sign this document after this expiration date

The Researcher is required to provide a copy of this consent to you.

____ ________ ____________
Signature of study volunteer/authorized representative* Date and Time when signed

I was present during the consent PROCESS AND signing of this agreement by the study volunteer or authorized representative

____ ______________
Signature of witness (required if consent is presented orally or at the request of the IRB) Date

____ ______________
Signature of Translator Date

____ ________ __________
Signature of researcher or designate Date and Time when signed

Study Volunteer Initials

* If signed by agent other than study volunteer, please explain below.

____
Study Volunteer Initials

Lifespan Affiliate Site where research will be conducted

☐ Rhode Island Hospital
☐ Bradley Hospital
☒ The Miriam Hospital
☐ Newport Hospital
☐ Gateway Healthcare

Agreement to Participate in a Research Study
And Authorization for Use and Disclosure of Information

_______________ Committee #

_______________________ Name of Study Volunteer

Study Title: A Phase 1, Single Center Study to assess the safety of MB66, a Combined Anti-HIV (VRC01) and Anti-HSV (HSV8) Monoclonal Antibody Film for Vaginal Application as Microbicide (MB66-01)

Study Number: MB66-01 – Segment B

Principal Investigators: Susan Cu-Uvin, M.D. , 401-793-7152

Study Nurse: Helen Patterson, 401-793-4771

You are being asked to take part in a research study. All research studies at Lifespan hospitals follow the rules of the state of Rhode Island, the United States government and Lifespan. Before you decide whether to be in the study, you and the researcher will engage in the "informed consent" process. During this process, the researcher will explain the purpose of the study, how it will be carried out, and what you will be expected to do if you participate. The researcher will also explain the possible risks and benefits of being in the study, and will provide other information. You should feel free to ask any questions you might have. The purpose of these discussions is for you to decide whether participating in the study is the best decision for you.

If you decide to be in the study, you will be asked to sign and date this form in front of the person who explained the study to you. This form summarizes the information you discussed. You will be given a copy of this form to keep.

3. <u>Nature and Purpose of the Study</u>
You are being asked to take part in a research project because you are a woman between the ages of 18 and 45 and in general good health.

Sex is the most frequent way HIV (Human Immunodeficiency Virus) and HSV-2 (herpes simplex virus) is transmitted. Condoms are a safe and effective way to prevent transmission of these viruses but for a variety of reasons, people do not use them consistently. In heterosexual sex, women have to rely on their male partners to agree to use a condom. There is a need for a product that women can use to prevent the transmission of HIV and HSV-2 that is convenient, easy to use, safe and effective.

This study will explore the safety and effectiveness of an investigational vaginal film as a way to deliver a medication that may prevent the transmission of viruses and other sexually transmitted diseases. The film is relatively small (about 2 inches square), flexible and flat, similar to wax paper. This film will contain a microbicide (a product that may reduce the chance of getting HIV and the herpes virus) that works when it comes in contact with the vagina tissue.

This study will help determine if this investigational vaginal product is safe and could be used to protect women against sexually transmitted infections. An investigational product means that it has not been approved by the Federal Food and Drug Administration (FDA) and is still being studied. At this time, we do not know if the product actually reduces the chance of infection or how long the protection will last.

The study product, a vaginal film, works by dissolving when it comes in contact with vaginal fluid. The film is relatively small (about 2 inches square), flexible and flat, similar to wax paper. The film contains antibodies made by plants that may be able to reduce a woman's chance of getting HIV, the virus that causes AIDS (human immunodeficiency virus), and HSV, the virus that causes herpes (herpes simplex virus).

The immune system of humans and animals naturally make antibodies to fight against infection; but it can take a lot of time and money for scientists to get enough animal antibodies to help prevent HIV and herpes infections. Plants do not naturally make antibodies, but scientists have found a way to use plants to produce antibodies that humans can use. It will be quicker and costs less money to make antibodies in plants, and it may be safer because, unlike animals, plants do not carry viruses that can infect humans.

In this study, you will be asked to use a vaginal film that contains antibodies produced by the *Nicotiana benthamiana* plant (a relative of the "tobacco plant," *Nicotiana tabacum*). While *Nicotiana benthamiana* does contain a small amount of nicotine, the plant goes through a cleaning process leaving only the antibodies (and small fragments of antibodies) that are then used in the vaginal film. The product that you will be using does not contain any nicotine and does not have any of the cancer causing effects of tobacco products. Antibodies made by this plant have already been shown to be effective at preventing HIV and herpes in laboratory studies. As we continue, when we say "drug" we are referring to these antibodies, which are the active ingredients in the study product.

We expect to enroll about 30 subjects into this Segment of the study. The study is sponsored by LeafBio, Inc., and funded by the National Institutes of Health (NIH).

4. <u>Explanation of Procedures</u>: If you take part in this study, you will have 5 study visits (including today's screening visit) and one phone interview over the span of approximately 4 to 9 weeks. You will be provided with vouchers for food during some of your visits. The visits are outlined below.

Visit 1 (Screening) – This visit will take up to 2 hours. During this visit you will review and sign this informed consent document. During this visit, the study nurse will give you detailed

information about the study and what you will be required to do and answer any questions you may have. You will be asked to provide contact information and answer screening questions to find out if you are eligible for the study.

This study requires a period of abstinence (not having sexual intercourse). You must agree to be abstinent from 5 days before Visit 2 through Visit 5 (which will typically be approximately 20 total days) and to use condoms for vaginal intercourse from Visit 5 until 28 days after your enrollment visit (Visit 2). During this time (between Visit 5 and 28 days after Visit 2) you cannot use a spermicide, a diaphragm or a NuvaRing as contraception. Acceptable forms of birthcontrol are: condom, hormonal contraception (pills, implants, injection, and patches), IUD and male or female sterilization.

The importance of abstinence will be discussed and you will need to agree to not have sexual intercourse to take part in the study.

You will also be required to abstain from <u>any</u> vaginal insertions such as douches and sex toys. You are allowed to use tampons during your period.

Procedures during this screening visit will include:

- You will be asked questions about your general health, any symptoms you may be having, and medications you are taking.
- If you are 21 or older and have NOT had a Pap smear in the last 36 months (3 years), you will have a Pap smear done
- You will have a physical exam, which will include your height, weight, temperature, blood pressure and heart rate.
- You will provide a urine sample to test for pregnancy, urinary infection, and STDs.
- You will have about three tablespoons of blood drawn for routine blood tests, screening for syphilis, and for before-treatment levels of the study drug.
- You will have a pelvic exam and tests for sexually transmitted diseases (STDs).
- During the pelvic exam you will have a procedure called a cervicovaginal lavage (CVL). In this procedure, about 1 teaspoon of a salt solution (which matches the normal salt content in your own body fluids), will be put in the cervicovaginal area (the area at the top of the vagina where the cervix is located) for a few seconds and then collected for testing.
- A test called a "Rapid HIV test" will be done. A padded device (similar to a toothbrush) is placed between your cheek and outer gum. After gently swabbing your mouth, the padded end is then inserted into a solution vial: the result appears on the result window within 20 minutes. As part of the testing procedures, the research staff will ask you questions about your sexual behavior and/or treatment history. The questions the research nurse asks will be similar to questions you might normally be asked when being examined by a doctor regarding reproductive and sexual health. The questions and discussion you have with the research staff are part of a pre/post-test counseling procedure required by the Rhode Island Department of Health when testing for HIV and other sexually transmitted diseases. These questions and discussion will include talking about your sexual behavior.

The pregnancy, HIV and STD tests must be negative for you to enter the study.

If you are menstruating at the time of a study visit, the visit will be rescheduled. We will try to schedule all study visits in between the periods of bleeding.

Visit 2 (Enrollment) This visit will be scheduled within 45 days of the screening visit and may last up to 6 hours. During this visit the study staff will ask you about changes to your contact information and about any symptoms or other health problems you are currently experiencing. Abstinence will be reviewed. You will be assigned to one of two groups: 1 group will receive the vaginal film with the study medication and one group will receive a placebo. This will be a vaginal film that does not have active ingredient. You will not know which group you have been assigned to.

Procedures during the enrollment visits will include:

- You will have a short physical exam including temperature, blood pressure and heart rate.
- Collection of urine sample to test for pregnancy and to test for infection
- You will have a pelvic exam to look for any signs of bleeding and 3 separate samples of vaginal fluid will be taken using vaginal swabs to measure bacteria in your vagina and to measure the pH (level of acidity) of your vagina. Tear Flo test strips (pieces of absorbent paper) will be held in contact with the vaginal tissue at four places to sample drug level before the vaginal film is inserted. The vaginal film study product will be inserted during this exam.
- At 1 and 4 hours after the insertion of the vaginal film, the Tear Flo sampling procedure will be repeated to test for the drug levels in the vaginal fluid.
- You will be asked about any vaginal or other symptoms or discomforts before leaving the clinic.

Visit 3 (Follow-up) – This visit will occur one day following visit 2 – This visit may last up to 2 hours. During this visit the study staff will again discuss abstinence and ask you how you are doing with the vaginal film. Procedures during this visit will include:

- You will have a short physical exam including temperature, blood pressure and heart rate.
- A urine sample will be tested for urinary tract infection
- Up to about 3 tablespoons of blood will be taken to test for the presence of the study drug. Other tests may be performed on the blood if indicated.
- You will have a pelvic exam to look for any signs of bleeding and 2 separate samples of vaginal fluid will be taken using vaginal swabs to measure bacteria in your vagina and to test the acidity of the vagina. Tear Flo test strips will be used to sample drug level as described above
- A CVL procedure will be done (as described in the Screening visit section).
- You will be a given instructions, including a short video on how to insert the next 8 films yourself at home and you will be given a supply of 8 films to take with you in a container that records when you remove each film for insertion.

Phone call 2 to 3 days after Visit 3 – The study staff will call you to see how you are doing and if you are having any vaginal or other problems. If you are having symptoms that make you or the

study staff concerned that you need to be examined, you will be asked to come in to the clinic for an extra visit. You will be reminded of the need to be abstinent.

Visit 4 (Follow-up)- This visit will occur 1 week after visit 2 and 24 hours after you inserted the last film – The study staff will ask if you have noticed any vaginal or other problems, and talk to you about continued abstinence.
Procedures during this visit will include:

- You will have a short physical exam including temperature, blood pressure and heart rate.
- A urine sample will be taken to test for pregnancy and to test for infection.
- About 3 tablespoons of blood will be taken to test for the presence of the study drug and for other routine blood tests. Other tests may be performed on the blood if indicated.
- You will have a pelvic exam to look for any signs of bleeding and 2 separate samples of vaginal fluid will be taken using vaginal swabs to measure bacteria and the PH (level of acidity) of your vagina. Tear Flo test strips will be used to sample drug levels as described in Visit 2.
- A CVL procedure will be done (as described in the Screening visit section).
- You will fill out a computer based questionnaire on your experience using the study film

Visit 5 (Follow-up) – Final visit, one week after 7th daily film dose
The study staff will ask if you have noticed any vaginal or other problems, and discuss the importance of condom use and provide you with a supply of condoms.
Procedures during this visit will include:

- You will have a short physical exam including temperature, blood pressure and heart rate.
- A rapid HIV-1 test with counseling before and after the test
- A urine sample will be collected to test for pregnancy and to test for infection.
- About 3 tablespoons of blood will be taken to test for the presence of the study drug and for other routine blood tests. Other tests may be performed on the blood if indicated.
- You will have a pelvic exam to look for any signs of bleeding and 2 separate samples of vaginal fluid will be taken using vaginal swabs to measure bacteria and the PH (level of acidity) in your vagina. Tear Flo test strips will be used to sample drug level
- A CVL procedure will be done (as described in the Screening Visit section).

Possible additional visits
If there are any unresolved safety concerns, you may be asked to return to the clinic for a follow-up visit after Visit 5.

Participants who test positive for HIV at screening or during the study will have further evaluation and will be referred to an HIV provider for care. If you are found to be infected with HIV, you will not be able to stay in the study. If you test positive for a sexually transmitted disease at screening, Dr. Cu-Uvin will provide treatment based on the Centers for Disease Control (CDC) guidelines or she will refer you for treatment by another provider.

Participants who have a positive pregnancy test also cannot enter or stay in the study. A referral for obstetrical care will be made and we will ask you to sign a medical release form so that we may inquire about the status of your pregnancy and the health of the newborn.

Procedures for Early Termination or Withdrawal Visits
If for any reason your study participation ends early, you will come back to the clinic for one more visit which will include the following:

- You will have a short physical exam including temperature, blood pressure and heart rate.
- A rapid HIV-1 test and counseling before and after the test
- A urine sample will be collected to test for pregnancy and to test for infection.
- About 3 tablespoons of blood will be taken to test for the presence of the study drug and for other routine blood tests. Other tests may be performed on the blood if indicated.
- You will have a pelvic exam to look for any signs of bleeding and 3 separate samples of vaginal fluid will be taken using vaginal swabs to measure bacteria in your vagina and to measure the pH (level of acidity) of your vagina. Tear Flo test strips will be used to sample drug levels as described in Visit 2.
- A CVL procedure will be done (as described in the Screening visit section).

<u>Cost for participating in this study</u>

There will be no cost to you for your participation in this study. Visits, procedures and medication that are part of the study procedures will be provided by the study.

However, if you receive services that are not part of this study and are considered routine clinical services, these services will be billed to your health insurance company, but you will be responsible for paying any deductibles, co-payments, or co-insurance that are a normal part of your health insurance plan. If you do not have health insurance, you will be responsible for those costs. These services include treatment for HIV, STD's and pre-natal care.

<u>Compensation</u>

You will be compensated for your time and travel. You will be compensated $50 for each visit with the exception of visit 2 for which you will be compensated $100. For visits where you will receive $100, payment will be sent to you in the form of a check. You will receive a voucher worth $50 for the other visits which can be redeemed at the Cashier's Office of The Miriam Hospital.

3. <u>Discomforts and Risks</u>

Cervicovaginal lavage and pelvic exam
There may be slight discomfort or pressure when the speculum is inserted. There is a risk of infection, but it is minimized by using proper sterile procedures.

MB66 (vaginal film)
Before human studies, products are tested in laboratory animals. Animals are given higher doses of the drug to help find out the amount of drug to use in humans. Some of the

laboratory animals given MB66 had bleeding, inflammation, and/or ulceration of the vaginal tissue. Vaginal bleeding or spotting and inflammation are possible risks with the use of MB66. Since this is the first time this drug will be used in humans, it is important to know there may be risks that are not yet known, including irritation, allergic reactions, or other side effects. There is a small chance that use of the study film could temporarily cause certain types of HIV tests to become "false positive", (meaning the test reading was positive, but you were not infected with HIV). This could happen if you absorbed enough of the antibody against HIV in the study film into your body to temporarily make this kind of HIV test falsely positive. Such a false positive result might be present for a few months after use of the study film. During the study we will use an HIV test that cannot be made falsely positive by use of the study film. But, if you were to have a different kind of HIV test outside the study within a few months of using the study film, there is a possibility that that test could give a false positive result. If this were to happen, the standard follow-up testing done along with that HIV test would show that this was a false positive test and that you were not infected with HIV.

Other study risks

Condoms are required for any vaginal intercourse that occurs within the two weeks after the final study visit, to prevent exposure of a partner to any residual MB66 film and to prevent pregnancy. However, condoms are less effective contraceptives than hormonal contraceptives, IUD, female sterilization, or sexual activity with a partner who has had a vasectomy. The greatest protection against pregnancy would be provided by a condom along with one of the methods listed above.

This investigation film has not been proved to prevent the transmission of HIV or HSV. It is important that you **always** use a condom to reduce your risk.

Interviews and Questionnaire

You may feel embarrassed by some of the questions

HIV and STD (sexually transmitted disease) test results

You may feel some anxiety or concern while waiting for your test results and feel sadness and depressed if you test positive for a sexually transmitted infection, including HIV. A positive HIV result may cause you to feel isolated, have thoughts of suicide or deny the results. You will have access to study personnel and the study clinician to discuss these concerns. If you need psychological services or other emotional support, you will be referred to a mental health provider by study personnel. Although animal testing indicates this is very unlikely, it is possible that the study drug may be absorbed into your blood and cause a temporarily false positive rapid HIV test. Follow up testing will show if it is a false positive test.

Blood draw

There may be bruising or swelling where the blood is drawn and you may experience light-headedness, discomfort and rarely, infection.

Loss of confidentiality

While we will do all that we can to protect your privacy (described in section 9), there is a slight risk of loss of confidentiality resulting in social harm when participating in studies.

4. Benefits
It is unlikely that you will receive any benefit from your participation in this study. However, others may benefit from the information learned in this study, by the development of safe and effective ways to prevent HIV and herpes virus transmission.

5. Alternative Therapies
There are no alternative therapies. You may choose not to be in this study.

6. Refusal/Withdrawal
It is up to you whether you want to be in the study. You are not required to enroll or participate. If you decide to participate, you can always change your mind and quit at any time. If you decide not to be in the study, or if you quit later, you will still be able to get the health care services you normally get. If you join, but later on the researcher or your doctor feels being in the study is no longer good for you, they may choose to take you out of the study before it is over. If new information becomes available that might change your mind about whether you want to stay in the study, the researcher will share this information with you as soon as possible.

The study doctor may withdraw you from the study for your protection if you are unable to comply with study procedures. You would also be withdrawn from the study in the event of pregnancy or a positive HIV-1 test. You would also be withdrawn if you require a prohibited medication while on this study.

If your participation is discontinued because the study product is discontinued, we would like your permission to continue to follow you.

You would also be withdrawn if the study is stopped by the NIH, government and institutional agencies. In the case of pregnancy, you will be asked to sign a medical release form as mentioned on page 5. You have the right to withdrawn your consent for medical record release by contacting the study doctor or nurse.

7. Medical Treatment/Payment in Case of Injury
A research injury is any physical or mental injury or illness caused by being in the study. If you are injured by a medical treatment or procedure you would have received even if you were not in the study,that is not a research injury. To help avoid research injury and added medical expenses, it is very important to follow all study directions carefully. If you do experience a research injury, Lifespan or the study doctor can arrange medical treatment for you. Such treatment will be paid for as described below.

If you have insurance and have a research injury that is not covered by the study, it is possible that some or all of the cost of treating you could be billed to your insurer. If your health insurance will not cover such costs, it is possible you would have to pay out of pocket. In some cases, Lifespan might be able to help you pay if you qualify for free care under Lifespan policy. However, Lifespan

has no policy to cover payment for such things as lost wages, expenses other than medical care, or pain and suffering.

The study sponsor, LeafBio, Inc, has no program to compensate or pay for a study related injury.

If you experience a medical injury, please call either the study nurse or doctor. Their phone numbers can be located on the first page of this form.

8. Rights and Complaints

Signing this form does not take away any of your lawful rights. If you have any complaints about this study, or would like more facts about the rules for research studies, or the rights of people who take part in research studies you may contact Janice Muratori in the Lifespan Office of Research Administration, at (401) 444-6246.

9. Confidentiality and Research Authorization for Use and Disclosure of Your Health Care Information

Your research records will be treated as private health care records and will be protected according to Lifespan privacy practices and policies that are based on state and federal law. In particular, federal law requires us to get your permission to use or disclose (release your information to someone outside of Lifespan) your health information for research purposes. If you sign this form you agree to be in this research study and you permit the use and disclosure of your health information for the purpose of conducting the research, providing treatment, collecting payment and running the business of the hospital. This permission has no expiration date. You may withdraw from the study at any time. However, if you do not want the researchers to use or disclose any further information in this study you must cancel permission in writing and may do so at any time. If you cancel your permission, you will stop taking part in the study and no new information will be collected about you. However, if you cancel your permission, it will not apply to actions already taken or information already collected about you by the hospital or the researchers before you canceled your permission.

Generally, the entire research record and any medical records held by the hospital may be used and released for research purposes. The following people or businesses/companies/ might use, release, or receive such information:

- The researcher and their support staff;
- The study sponsor LeafBio, Inc., and the study funder, the National Institutes of Health (NIH);
- Doctors, nurses, laboratories and others who provide services to you or the sponsor in connection with this study;
- The company or section of the U.S. government that is paying for the study and others they hire to oversee, administer, or conduct the research;
- The United States Food and Drug Administration, the Department of Health and Human Services, the Office of Inspector General, and the Office of Civil Rights; European Medicines Agency
- People who volunteer to be patient advocates or research volunteer protectors;

- Members of the hospital's administrative staff responsible for reviewing, approving and administering clinical trials and other healthcare or research activities.
- Accrediting Organizations

There are times when the law might require or permit Lifespan to release your health information without your permission. For example, Rhode Island law requires researchers and health care workers to report abuse or neglect of children to the Department of Children, Youth and Families (DCYF) and to report abuse or neglect of people age 60 and older to the Department of Elderly Affairs. Newly diagnosed cases of HIV will be reported to the RI Department of Health (RIDOH) as required by state law. Other STDs such as gonorrhea, chlamydia and syphilis must also be reported to the RIDOH as required by state law.

All researchers and health care providers are required to protect the privacy of your health care information. Other people and businesses/organizations that are not health care providers are not required by law to do that so it is possible they might release your information.

You have the right to refuse to sign this form and not participate in the research. Your refusal would have no effect on your treatment, charges billed to you, or benefits at any Lifespan health care site. If you do not sign, you will not be able to enroll in the research study and will not receive treatment as a study participant.

If you decide to quit the study after signing this form (as described in Section 6) no new information will be collected about you unless you gave us permission to do so. However, the hospital or the researchers may continue to use information that was collected before you quit the study to complete analysis and reports of this research.

A description of this clinical trial will be available on *http://www.ClinicalTrials.gov*, as required by U.S. law. This Web site will not include information that can identify you. At most, the Web site will include a summary of the results. You can search this Web site at any time.

Optional: Participation in Specimen Banking

As a participant in the main study, we will be collecting specimens (samples of vaginal fluid and blood). In addition to being analyzed as part of the Main Study, your Specimen may be useful for future research purposes. You are being asked to agree to this optional component (the "Specimen Banking Component") of the study if you are willing to allow your specimen to be saved or 'banked' for use in future research studies. If you agree to this optional Specimen Banking Component, you give permission for your specimen to be stored in a specimen bank. Along with the specimens, portions of your personal health information collected as part of the Main Study will also be stored. Your Specimen and personal health information may be stored and analyzed at Lifespan; or, they may be shared with researchers at other institutions or companies that may store them and use them for their own research. It is very unlikely that any future research performed using your Specimen would benefit you directly. However, the research may provide important medical knowledge that in the future could help patients with different medical problems.

At this time, we do not know what future research studies may be done using your Specimen.

____
Study Volunteer Initials

There is a very remote possibility that your Specimen and some associated data may become part of a process or product that ultimately has commercial value. For instance, the Specimen could be used to establish a cell line (a group of cells that are able to reproduce, sometimes indefinitely) that could be patented and licensed. There are no plans to provide financial compensation to you should this occur.

If you agree to this Specimen Banking Component, your Specimen will be stored for an indefinite period of time, until it is no longer usable. The Specimen may also be used to create a cell line, which would also be stored for an indefinite period of time. If you decide at some time in the future that you no longer wish your stored Specimen to be used in future studies, you have the right to request that the Specimen be withdrawn from the specimen bank. However, withdrawal cannot be guaranteed and may be impossible. For example, it is possible that the Specimen might no longer be identifiable as belonging to you, or it may have been used up, or it may already have been shared with other institutions or companies for their own research. To request withdrawal of your Specimen, please write to: Susan Cu-Uvin, MD., The Miriam Hospital, 164 Summit Ave, Bldg 1125 No. Main St.-Rear, Providence, RI 02906.

Refusal to participate in this optional Specimen Banking component will in no way affect your ability to receive any treatment or services offered as part of the Main Study, and will not have any effect on your other health care, the payment for your health care, or your health care benefits. **If you are willing to allow your Specimen to be banked for future research purposes, please indicate your consent by signing below.**

____ ________
Signature of study volunteer/authorized representative Date

For more detail about your privacy rights see the Lifespan Joint Privacy Notice which has or will be given to you.

SIGNATURE

I have read this informed consent and authorization form. ALL OF MY QUESTIONS HAVE BEEN ANSWERED, AND I WANT TO TAKE PART IN THIS RESEARCH STUDY.

By signing below, I give my permission to participate in this research study and for the described uses and releases of information. *I also confirm that I have been now or previously given a copy of the Lifespan Privacy Notice*

____
Study Volunteer Initials

This informed consent document expires on _________________.
DO NOT sign this document after this expiration date

The Researcher is required to provide a copy of this consent to you.

____ ________ ____________
Signature of study volunteer/authorized representative* Date and Time when signed

I was present during the consent PROCESS AND signing of this agreement by the study volunteer or authorized representative

____ ______________
Signature of witness (required if consent is presented orally or at the request of the IRB) Date

____ ______________
Signature of Translator Date

____ ________ __________
Signature of researcher or designate Date and Time when signed

* If signed by agent other than study volunteer, please explain below.
____

____
